# Beyond borders: A systematic review and meta-analysis of human-specific faecal markers across geographical settings

**DOI:** 10.1080/10643389.2025.2455031

**Published:** 2025-02-06

**Authors:** Leah R. Barrett, Paris Beasy, Yussi M. Palacios Delgado, John D. Boyce, Karin Leder, David T. McCarthy, Rebekah Henry

**Affiliations:** a Department of Civil Engineering, Monash University, Melbourne, Victoria, Australia; b Biomedicine Discovery Institute, Department of Microbiology, Melbourne, Victoria, Australia; c School of Public Health and Preventive Medicine, Monash University, Melbourne, Victoria, Australia; d Victorian Infectious Disease Service, Royal Melbourne Hospital, Melbourne, Victoria, Australia; e School of Environmental Sciences, University of Guelph, Guelph, Ontario, Canada

**Keywords:** Fecal pollution, human-specific fecal marker, marker performance, marker validation, microbial source tracking, Hyunjung Nick Kim

## Abstract

Human fecal waste is a global health risk associated with diarrheal diseases, responsible for approximately 1.2 million deaths annually. Microbial Source Tracking (MST) is a molecular method that evaluates environmental sources of fecal contamination, aiding quantification of this contamination and associated health risks. However, reported variations in global human gut microbiomes and geographic performance of human-specific fecal markers suggest that current MST targets may not have broad applicability across populations. This systematic review quantified the performance of human-specific fecal markers to identify those suitable for use across various geographic regions. We evaluated data from primary research articles, published before 18^th^ October 2023, identified through PubMed, Scopus, and Web of Science using PRISMA guidelines. 103 studies published between 1995 and 2023, spanning 34 countries, 6 continents, and 4 climate zones met inclusion criteria, with quantifiable performance metrics (sensitivity, specificity or accuracy) and a geographic testing location. Extracted data was analyzed to establish marker performance across geographic locations, climate zones, and development status. Over 80% were conducted in High-Income Countries (HICs) and >50% in temperate zones, primarily in the USA (43%), Australia (24%), and Spain (19%). *Bacteroides HF183* was the most commonly tested (*n* = 45 studies). However, no target consistently demonstrated sensitivity, specificity, and/or accuracy >80% across different settings. Consequently, a decision tree is presented supporting selection of appropriate human-specific markers for regional-specific baseline studies. This provides critical information to support new MST research, particularly in Low- and Middle-Income Countries (LMICs), assisting with informed decision and method selection for assessing risks of faecal derived pathogens.

## Graphical Abstract

**Figure:**
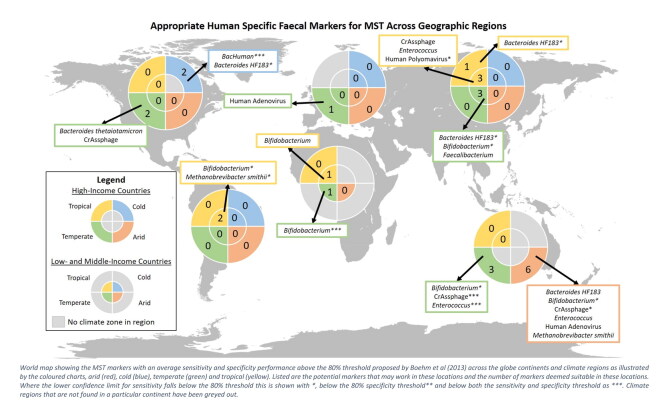

## Introduction

Every year, it is estimated that over 1.2 million people, including 440,000 children, die from preventable and treatable diarrheal diseases, primarily stemming from exposure to enteric fecal pathogens (World Health Organization 2024a). Contamination typically arises from contact either with fecal matter from an infected host animal or with feces from human sources, with the latter generally considered to have the highest potential risk for human infections (Mertens et al., 2024). Diarrheal-related mortality rates are therefore disproportionately high in Low- and Middle-Income Countries (LMICs), where communities frequently lack access to clean drinking water and adequate sewage and sanitation infrastructure.

Monitoring and defining the source and prevalence of fecal pollution is an important component of understanding the potential human health risks and is an important starting point for implementing targeted mitigation and management strategies to reduce disease acquisition and transmission. Measurement of Fecal Indicator Organisms (FIOs) such as *Escherichia coli* (*E. coli*) and enterococci is the standard method recommended by the World Health Organization (2024b) for risk assessment of environmental fecal contamination. Fecal indicators are a naturally occurring part of the mammalian gut microbiome. The detection of culturable FIOs should therefore be indicative of recent fecal contamination (Horan, 2003). However, FIOs are not indicative of source, often do not correlate with the presence of enteric pathogens and have been increasingly found to naturalize in environments outside of the host; thereby not providing an accurate measure of risk (Devane et al., 2020; Ferguson & Signoretto, 2011; Gerba, 2009; Holcomb & Stewart, 2020).

Microbial source tracking (MST) offers a potential alternative to measurement of FIOs. Quantitative MST is an increasingly popular suite of molecular methods that support source-specific quantification of fecal contamination (Harwood et al., 2014). This allows evaluation of potential human health risks, while providing evidence of the source of pollution (Santo Domingo et al., 2007; Zhang et al., 2019). Broadly, MST can be divided into library-dependent and library-independent methods (Hagedorn et al., 2011; Stoeckel & Harwood, 2007). In brief, library-dependent methods involve isolate identification of bacterial cultures from fecal and environmental samples, which are then compared against a “library” or “community profile” of bacterial strains from known fecal sources. This comparative database, often constructed using amplicon sequencing approaches, is typically region-specific, resulting in high accuracy (Field et al., 2003; Hagedorn et al., 2011). However, the major limitation of library-dependent methods is the need to create a new reference library for each new study location, coupled with sanitary surveys to identify new or relevant fecal sources. This process can be both time-consuming and costly (Ahmed, 2007).

In contrast, library-independent methods use the presence of a single organism or sequence, often termed a marker, to identify the source of fecal pollution. The use of a single marker makes library-independent methods cheaper and faster than the library-dependent alternatives. Consequently, library-independent methods are often more feasible in low-resource settings (Meals et al., 2013). However, their predictive power relies on the chosen marker accurately differentiating human from nonhuman fecal contamination. As such, reduced accuracy is a concern due to the potential for marker detection in nonhuman fecal samples and regionally specific changes in gut microbiome composition, which can result in false-positives, false-negatives, and off-target amplification (Hagedorn et al., 2011; Oliveira & Pamer, 2023). As previous studies have demonstrated that an individual’s microbiome is influenced by genetics, age, diet, and environmental conditions (Oliveira & Pamer, 2023). Thus, people who share similar lifestyles, cultures or environments tend to have more similar gut microbiomes than those living in different regions with distinct cultural, dietary, genetic, and environmental influences (Deschasaux et al., 2018; Parizadeh & Arrieta, 2023; Yatsunenko et al., 2012). Therefore, geographic differences, including those between countries, continents, climate zones and development status, are likely to impact gut microbiome makeup. Consequently, although marker performance is generally assumed to be globally relevant (Hagedorn et al., 2011) many markers have not been tested across geographic regions and these differences likely influence presence and prevalence of specific marker organisms in the gut microbiome (García-Aljaro et al., 2019).

This systematic review and meta-analysis aims to investigate the performance of different MST markers across diverse geographic, climatic, and socio-economic settings. We focus specifically on human-specific library-independent MST methods for assessment of health risk posed by human fecal pollution. Importantly, identified markers were assessed for factors including sensitivity, specificity and accuracy across these different geographic regions. This information was applied to create a decision tree and matrix, enabling targeted selection of MST markers to specific geographic regions. This approach aims to enhance risk assessments and mitigation strategies in a cost-effective manner.

## Methods

### Search strategy and selection criteria

A preliminary search and meta-analysis of PubMed, Scopus and Web of Science databases was conducted on 23^rd^ May 2023 to identify which human-specific markers to include in the systematic review (Supplementary Material 1.0). The systematic search strategy followed PRISMA guidelines (Page., 2021a; Page., 2021b) and was conducted using PubMed, Scopus, and Web of Science on the 18^th^ October 2023. Markers were grouped by broad name (and their common abbreviations shown in parentheses) and not by specific primer identifiers;

- *BacHuman*
- *Bacteroides HF183 (HF183)*
- *Bacteroides stercoris F1 (BsteriF1)*
- *Bacteroides thetaiotamicron (B. theta)*
- *Bifidobacterium*
- *Bifidobacterium catenulatum (B. catenulatum)*
- CrAssphage
- *Enterococcus*
- *Faecalibacterium*
- Human Adenovirus (HAdV)
- Human Polyomavirus (HPyV)
- *HumM2*
- *Methanobrevibacter smithii (M. smithii)*

As such search terms encompassed variations of; marker name, MST and performance metrics. Where a marker has the potential to be known by multiple names, all names were included within the search. Examples of full search terms, including database-specific syntax for HPyV are outlined in Supplementary Material 2.0. No date range, language restrictions or publication limitations were imposed so that all relevant studies on the 13 pre-selected markers were identified. When reviews were identified, the original research articles, if not already identified *via* the primary search, were included in place of the review.

Inclusion and exclusion criteria were developed (Supplementary Material 2.0). Where a paper met the inclusion criteria for having a quantifiable MST marker performance metric, but had no specific mention of geographic location, the authors were contacted for clarification. All requests for information were made on or before 1^st^ March 2024 and all responses received by 1^st^ June 2024 were taken into consideration.

Covidence (Veritas Health Innovation, 2024) and Endnote Version 20 (The EndNote Team, 2023) were used to identify and remove any duplicate publications. Two independent reviewers (LB and PB) processed articles using Covidence. Each unique article underwent title and abstract screening against inclusion/exclusion criteria. Where it was unclear if the article met the selection criteria, this was marked as “maybe” and passed through to the next stage of screening for clarification. Once an article had passed initial screening, the full texts were reviewed for relevance. References from the articles remaining after full text screening were checked for relevance, by utilizing the tool Research Rabbit (The ResearchRabbit Team, 2024). Any referenced article, not previously identified, that met the inclusion criteria was included in the next stage of analysis. Any difference of opinion between reviewers was resolved through discussion until a unanimous decision was made.

### Data analysis and statistics

A data extraction and risk of bias assessment form was created in Microsoft Office Excel 2019. The risk of bias assessment was based on guidelines provided by Hong et al. (2018) for quantitative descriptive studies. This assessment consisted of five criteria related to the sampling strategy, sample population, and statistical analysis.

The data extraction portion of the form was designed to target all key information needed for meta-analysis. This included information of the study location, sample type (e.g., human feces, wastewater, and animal species) and quantitative values for sensitivity, specificity and/or accuracy. A full example of the data extraction and risk of bias form is available in Supplementary Material 2.0.

Data analysis was conducted using Microsoft Office Excel 2019 and RStudio 4.3.0 to generate figures such as heat maps and bar charts. For analysis the following definitions were applied; Target Sample: Human feces or wastewater samples. Nontarget sample: animal feces or any nonhuman fecal samples. Sensitivity: the ability for a marker to detect a source when it is present (true positive). Specificity: the ability for a marker to correctly identify the fecal source (true negative) Accuracy: the proportion of true results, either true positive or true negative that were correctly identified. The following equations were used to calculate these performance measures (Schiaffino et al., 2020).

$$\text{Sensitivity}=\frac{\text{Number of Known Target Samples Positive for Marker}}{\text{Number of Known Target Samples Tested}}$$

$$\text{Specificity}=\frac{\text{Number of Known Non Target Samples Negative for Marker}}{\text{Number of Known Non Target Samples Tested}}$$

$$\text{Accuracy}=\frac{\text{True Positive Samples}+\text{True Negative Samples}}{\text{All Samples Tested}}$$

To address variability in marker performance, 95% confidence intervals were calculated for sensitivity, specificity, and accuracy to illustrate the precision of these performance estimates. Additionally, Pearson’s correlation analysis was performed to create a correlation matrix, which is included in the Supplementary Material for further reference.

## Results

### Data overview

**Figure 1.**
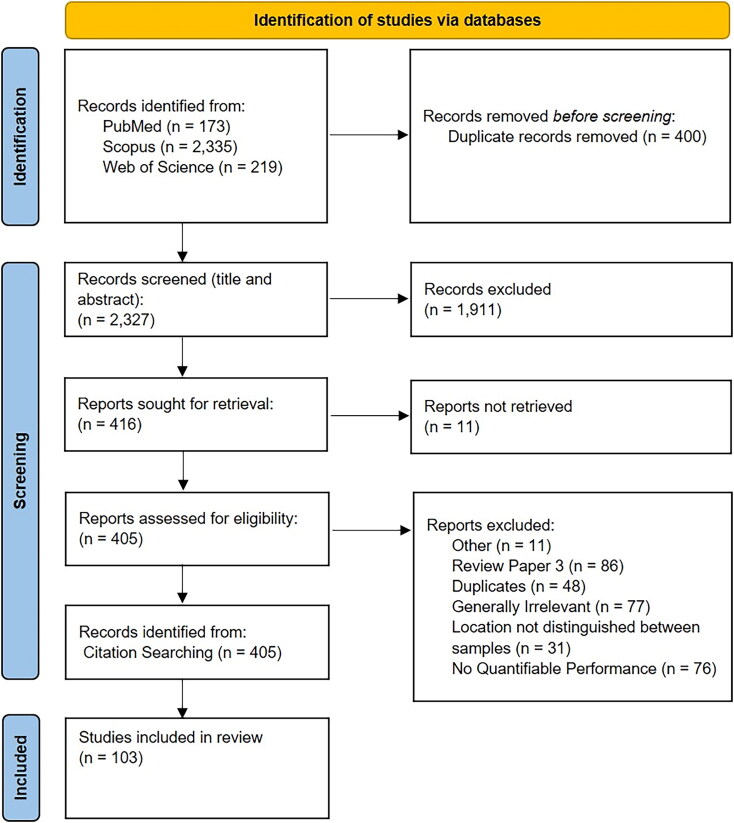
Summary of the screening process that lead to the identification of 103 papers for inclusion in the systematic review and meta-analysis using PRISMA 2020 flow diagram template (Page., 2021a; Page., 2021b).

Of 2,727 papers found through searchers in PubMed, Scopus, and Web of Science, 400 duplicates were removed (Figure 1). A further 1,911 were excluded based on title and abstract screening, resulting in 416 for secondary review. After further screening and manual reference checks, there were 103 papers that met the inclusion criteria.

Thirteen human MST markers were investigated within this review. In total these had been tested against 8,422 target samples (human derived) and 16,174 nontarget samples (nonhuman derived) for inclusion and analysis within the study (Table 1). *HF183* was the most frequently applied human marker appearing in 45 papers, while HPyV, HAdV, and CrAssphage were reported in 20, 17, and 16 papers, respectively. In contrast BacHuman and *Faecalibacterium*, were only reported in one paper each.

**Table 1.** Number of papers identified for each marker and the total amount of target and nontarget samples used for analysis of the total 103 papers identified.

Organisms	Number of papers	Target samples	Nontarget samples
*BacHuman*	1	16	70
*Bacteroides HF183*	45	2370	6689
*Bacteroides stercoris F1*	2	192	362
*Bacteroides thetaiotamicron*	12	532	1410
*Bifidobacterium*	12	585	833
*Bifidobacterium catenulatum*	2	71	126
CrAssphage	16	2129	3857
*Enterococcus*	11	597	2669
*Faecalibacterium*	1	110	540
Human Adenovirus	17	485	967
Human Polyomavirus	20	319	658
*HumM2*	3	36	249
*Methanobrevibacter smithii*	11	2277	1094
Total		8,422	16,174

### Data distribution

**Figure 2.**
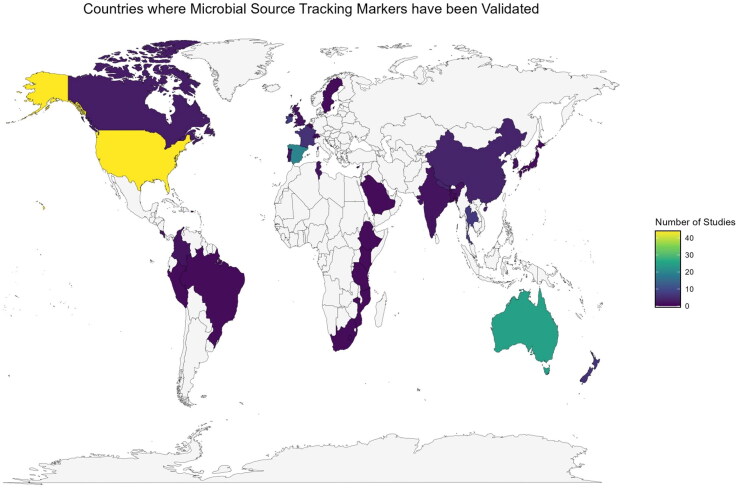
A heat map of the world showing regions where quantifiable MST research on at least one of the 13 markers has been conducted. Number of studies are colored from; purple (low) to yellow (high) with regions where no quantifiable MST research has been conducted shown in grey. Created in RStudio version 4.3.0.

**Figure 3.**
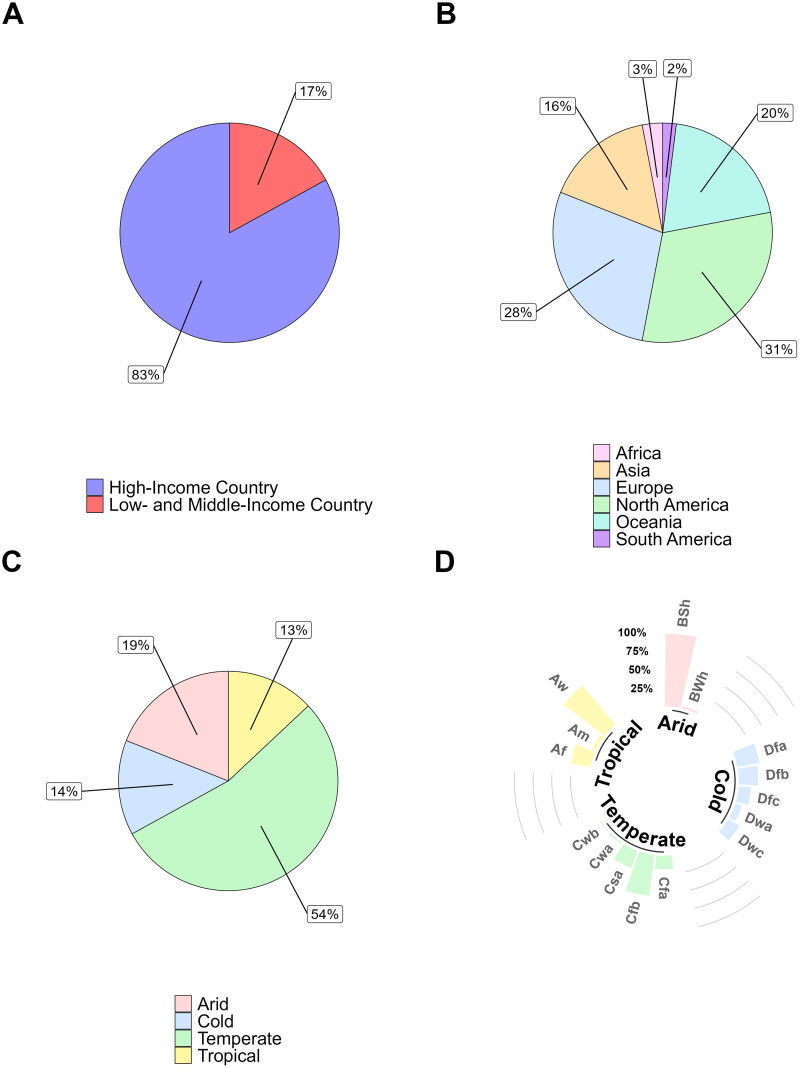
Contribution of research identified from the systematic review categorized by: A) Development status; B) Continent; C) Climate zones and D) Sub-climate zone. Sub-climate zones were defined as: arid including BSh (Hot semi-arid (steppe)) and BWh (Hot deserts) regions; cold including Dfa (Hot-summer humid continental), Dfb (Warm-summer humid continental climate), Dfc (Subarctic), Dwc (Monsoon-influenced subarctic) and Dwa (Monsoon-influenced hot-summer humid continental) regions; temperate including Cfb (Temperate oceanic), Csa (Hot-summer Mediterranean), Cfa (Humid subtropical), Cwa (Monsoon-influenced humid subtropical) and Cwb (Subtropical highland or temperate oceanic with dry winters) regions; and tropical including Aw (Tropical savanna wet), Af (Tropical Rainforest) and Am (Tropical Monsoon) regions.

The United States (*n* = 44), Australia (*n* = 25), and Spain (*n* = 20) were identified as conducting the largest number of total studies assessing performance of these human-specific markers (Figure 2). This was further reflected in data distribution across climatic zones, continents, and development statuses (Figure 3). The results revealed a clear bias toward marker application in High-Income Countries (HICs) (83%), temperate climates (54%), and regions including North America (31%), Europe (28%), and Oceania (20%). It was noted that the ‘Oceania’ proportion of studies was predominantly driven by data from Australia (81%) and New Zealand (19%). Overall, Asia (16%), Africa (3%), and South America (2%) had the lowest reported assessment of the 13 target MST markers.

Climate-based analysis demonstrated that the majority of arid regions in which MST markers had been applied were classified as “hot semi-arid” (97%) (Figure 3D). Temperate regions were predominately “temperate oceanic” (55%) and tropical regions were mainly “tropical savanna wet” (68%). Cold climate zone data was split between “hot-summer humid continental” (30%), “warm-summer humid continental climate” (26%), “subarctic” (17%), “monsoon-influenced hot-summer humid continental” (9%) and “monsoon-influenced subarctic” (17%).

### Performance of MST markers

#### Global performance

**Figure 4.**
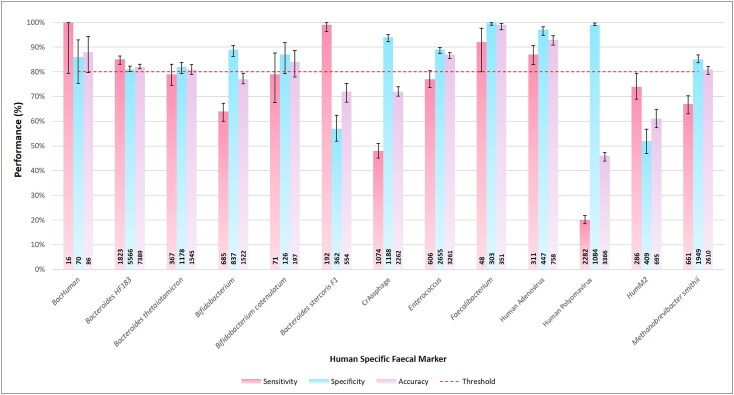
Sensitivity (Pink), Specificity (Blue) and Accuracy (Purple) of all 13 human-specific fecal markers at a global scale with a dotted red line indicating the 80% performance threshold set out by Boehm et al. (2013). The number of samples for each marker analysis are shown in each bar. Error bars represent the 95th percentile upper and lower confidence interval of each performance statistic.

The global sensitivity, specificity, and accuracy of each of the 13 markers was calculated and compared to the 80% sensitivity and specificity threshold established by Boehm et al. (2013) (Figure 4). Four markers met or exceeded this threshold at a global scale. These were: *BacHuman* (100, 86, and 88%), *HF183* (85, 81, and 82%)*, Faecalibacterium* (92, 100, and 99%) and HAdV (87, 97, and 93%). However, it was noted that despite the median sensitivity and specificity being >80%, the lower confidence limit for *BacHuman* (79, 75, and 80%) did not meet the 80% threshold for all three-performance metrics based on this 95% confidence interval.

#### Performance across geographic regions

**Figure 5.**
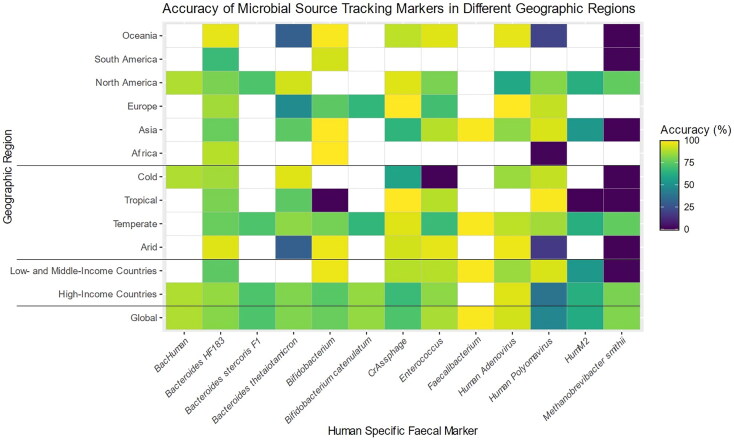
A heat map showing the percentage accuracy of each of the 13 human-specific fecal markers across all geographic groupings from low accuracy (purple) to high accuracy (yellow) with regions with no data available for a specific marker shown in white. Created in RStudio version 4.3.0.

Geographic assessment demonstrated that accuracy varied by individual marker and region (Figure 5, Supplementary Material 4.0). As such, the total number of publications that report on the performance of these markers and the location of these studies influences the potential for each marker to be validated in certain geographic regions. It was observed that *HF183, Bifidobacterium*, CrAssphage, Enterococcus, HPyV, and *M. smithii* had been tested across diverse geographic regions. In contrast, *BacHuman*, *BsteriF1*, *B. catenulatum*, *Faecalibacterium*, and *HumM2* had only been tested in a limited number of regions, specifically HIC’s, North America and temperate climate zones.

#### Performance across development status

Although the majority of research has been conducted in HICs, only three of the 13 markers assessed within this review (*BacHuman*, *HF183*, and HAdV) met the 80% sensitivity and specificity threshold in HIC contexts, whereas in LMICs, five of the 13 markers were sufficient for use (*Bifidobacterium,* CrAssphage, Enterococcus, *Faecalibacterium*, and *M. smithii*). HICs in tropical regions or within Africa, Asia, or South America had reported no marker that is validated to perform at sufficient levels. No marker could be identified in LMICs in arid/cold regions and/or within Europe, North America, or Oceania.

#### Performance across climate zone

Climate and sub-climatic correlative analysis did not show uniformity (Supplementary Material S4.14). Further investigation highlighted that significant climate relationships were driven by the number of studies conducted within the region. Consequently, evaluation based on sub-climate zones was not pursued further and represents a significant limitation of currently available datasets.

Arid regions had the largest range of potential markers meeting the performance thresholds (*n* = 6). However, when examining the relationship between climate zones, we see a lack of marker validation in arid regions, except in Oceania, where six potential markers are indicated—the highest of any climate/continent combination (*HF183, Bifidobacterium*, CrAssphage, *Enterococcus,* HAdV, and *M. smithii*).

Cold regions show a scarcity of available markers, with only two potential markers, *BacHuman and HF183*. Temperate zones only have three markers performing sufficiently, CrAssphage, *Faecalibacterium*, and HAdV, but do have potential markers for every continent except South America, which only has potential markers for its tropical regions.

While tropical regions have the second highest number of potential markers (*n* = 5, *Bifidobacterium*, CrAssphage *Enterococcus*, HPyV, and *M. smithii*), they are only found to perform at or above the 80% performance threshold in tropical LMICs, with the exception of *HF183* in tropical HICs in Asia.

#### Performance across continent

 All six continents had at least one marker that met the 80% sensitivity and specificity threshold. Oceania and Asia had the greatest number of human specific markers with five and four respectively. North America follows this with three markers (*BacHuman*, *B. theta* and CrAssphage) followed by Europe and South America with two each (CrAssphage and HAdV in Europe and *Bifidobacterium* and *M. smithii* in South America) and finally Africa with only one (*Bifidobacterium*). It is important to note that Oceania, North America and Europe markers were equivalent to those previously identified as acceptable for HIC application. In contrast, Africa and South America human-specific markers were LMIC specific. Asia was the only continent with a marker, *HF183*, performing across the performance threshold for both HICs and LMICs.

#### Inclusive approach to MST marker selection

The results of this study emphasize the critical need for validation of human-specific MST markers across varying developmental statuses, climate zones, and geographic locations. Notably, no single marker was validated to perform consistently across all regions, highlighting a significant limitation in the current body of research. Practitioners should carefully consider these limitations before undertaking region-specific analyses.

**Figure 6.**
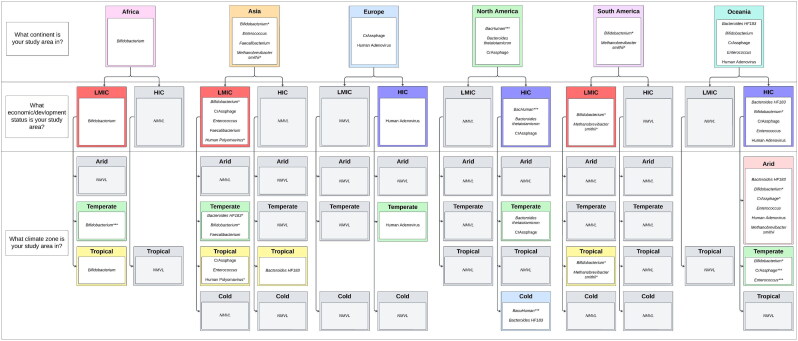
A decision tree of human-specific fecal markers that perform at or above the 80% threshold (Boehm et al. 2013) in a combination of continent, development/economic status and climate zones. Data with a lower confidence limit below the 80% threshold is indicated by * for sensitivity, ** for specificity and *** for both. Where no marker met the threshold or is validated in the location this is shown with NMVL (No Marker Validated in Location). Begin by identifying the continent where your study area is located. Next, the developmental status of the region and finally climate zone relevant to the study. If, at any point in this process, you receive a result of NMVL, this indicates that no marker has been validated to meet the 80% performance threshold for the given combination of continent, development status, and/or climate, where this happens please refer to Figure 7 and/or Supplementary Material S4.16.

To address this gap, we developed a decision tree (Figure 6) as a practical tool to assist researchers in selecting appropriate MST markers. This decision tree incorporates three core factors: the continent of the study area, the region’s developmental status (HIC or LMIC), and the climate zone. Researchers should begin by identifying the continent of their study, followed by the developmental status and climate zone. This structured approach aims to simplify the selection process for MST markers tailored to specific geographic and environmental contexts.

**Figure 7.**
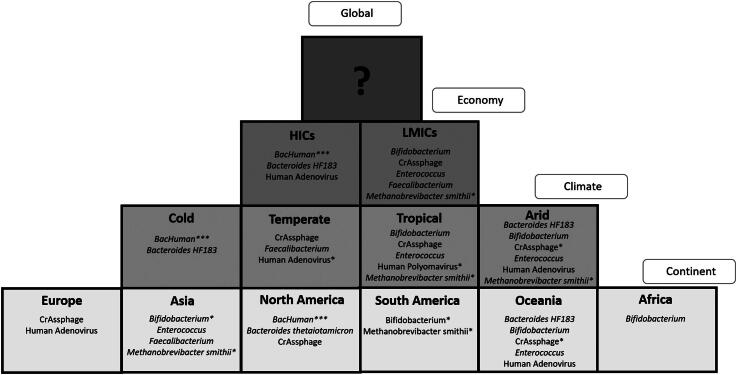
A decision pyramid matrix showing the markers that meet the 80% threshold (Boehm et al. 2013) for both sensitivity and specificity in individual geographic regions, split via global, development status, climate region and continent. Markers that meet the threshold, but for which the lower confidence limit falls below the 80% threshold, are identified by * for sensitivity, ** for specificity and *** for both.

If the decision tree yields a result of NMVL (No Marker Validated for Location), this indicates that no marker has met the 80% performance threshold for the specified combination of factors. In these cases, the decision matrix (Figure 7) serves as an alternative resource. The matrix provides insights into markers validated under individual conditions, such as specific climate zones, continents, or developmental statuses, without requiring a combination of these factors. While the matrix does not offer a definitive marker for all combinations, it lists promising targets that can be tested further in the relevant context.

This stepwise process, beginning with the decision tree, consulting the decision matrix, and referencing supplementary materials (S4.16), provides a comprehensive framework for marker selection. Notably, 26 out of 37 possible combinations of developmental, climatic, and geographic factors returned an NMVL result. This finding underscores the limited coverage of existing validated markers and the need for continued research efforts to expand the applicability of MST markers.

Importantly, this approach highlights that no single marker performs universally across all combinations of climate zones, continents, and developmental statuses. This reinforces the necessity of region-specific validation efforts and the development of markers tailored to diverse conditions. By leveraging the decision tree, decision matrix, and supplementary materials, researchers can make informed, evidence-based decisions about the most suitable MST markers for their studies, ensuring accuracy and reliability in a variety of global contexts.

## Discussion

MST has the potential to enhance the identification of both human and animal fecal pollution in the environment, though this review focuses on human-specific MST. This could significantly impact the burden of diarrheal disease, which remains high in regions of the Global South, helping to reduce morbidity and mortality (World Health Organization, 2024a). However, the success of this method is dependent on the availability and the applicability of the assays applied when conducting an environmental assessment.

To address this issue, we have developed a decision tree to assist researchers in selecting appropriate human-specific MST markers for their specific study locations. Due to the current data limitations, researchers may encounter a “NMVL” result. To address this, we have also created a secondary decision matrix to expand the range of potential markers for various study locations. Nonetheless, this approach is not a permanent solution. It is crucial to recognize and address these research disparities to develop effective human-specific MST strategies that cater to the unique needs and conditions of different geographic regions.

Observed variations in human-marker sensitivity likely stem from differences in the human gut microbiome across the globe. For instance, HICs, commonly located in the Global North, are typically characterized by Westernized cultures, similar diets, built environments, and cold or temperate climates (Deschasaux et al., 2018; Gupta et al., 2017; Wilson et al., 2020). In contrast, LMICs, usually found in the Global South often have tropical and arid climates, with cultural practices and diets more dependent on the environment (Brewster et al., 2019). As these factors have been found to directly impact the gut microbiome (Oliveira & Pamer, 2023), it is predicted that these differences may influence marker presence and prevalence in the gut microbiome of different populations (García-Aljaro et al., 2019).

However, the observed differences in marker specificity are a result of marker presence and amplification in nonhuman species. Similarities between human and animal microbiomes are often attributed to close contact between the two. A systematic review by Abdolghanizadeh et al. (2024) shows that having a pet, specifically a cat or dog can influence the human gut microbiota and vice versa. Furthermore, hygiene practices significantly impact gut microbiome composition, with both positive and negative effects. In LMICs, where Water, Sanitation, and Hygiene (WASH) resources are often limited and variable, and where environmental pollution can be considerable, these factors likely contribute to increased horizontal transmission of microbiota between species (Trinh et al., 2018). Research in Kenya further illustrates this phenomenon, revealing that children and local cattle share similar gut microbiota (Mosites et al., 2017). This cross-species microbiome interference makes it challenging to accurately identify the source of fecal contamination in the environment and, consequently, to design and implement targeted management and mitigation strategies (Ahmed et al., 2019). Therefore, for a marker to be effective, it needs to demonstrate not only high sensitivity but also sufficient specificity to its target species to minimize the risk of misclassification. These findings underscore the importance of conducting regional studies that account for environmental and cultural differences, which can impact microbiome composition and, consequently, marker carriage in both humans and nontarget species.

While this paper provides a valuable starting point for marker selection, it does not validate every human-specific MST marker. In fact, our preliminary search for this review identified >50 human specific markers (Supplementary Material 1.0) As such, we recommend that researchers consult existing literature, especially if the decision tree results in “NMVL,” to identify additional markers used in similar geographic contexts and verify their validation status. If researchers encounter markers that have not been validated or are not validated in specific locations, including those discussed in this review, they should, when possible, undertake validation across diverse geographic contexts, before use. Additionally, as the field of research is continually evolving, with new markers being identified and developed it is crucial to validate these emerging markers to ensure their reliability and effectiveness. This approach will help ensure that markers are used appropriately and effectively in various field settings.

### Limitations

Our review highlights a critical limitation in the current state of MST research; no single human-specific marker has been validated to consistently achieve >80% sensitivity and specificity across all geographic and climatic contexts. This finding highlights the inherent complexity of MST application, due to variation in gut microbiome composition, influenced by a multitude of factors, including diet, climate, and cultural practices (Dill-McFarland et al., 2019; Gacesa et al., 2022; Martinez-Guryn et al., 2019; Oliveira & Pamer, 2023). While a global marker may be ideal, it is increasingly clear that achieving such a goal may be unrealistic. Instead, research efforts must focus on validating regionally specific markers to ensure MST methods are reliable and effective for use across diverse contexts.

We also identified a substantial bias in study locations, with the majority of MST validation studies concentrated in HICs, temperate zones, and regions of North America and Europe. This skewed distribution leaves critical gaps in data coverage, particularly in LMICs and underrepresented regions such as Africa (3% of studies) and South America (2% of studies). These disparities limit the global applicability of existing MST methodologies and underscore the urgent need for validation studies in LMICs and less-studied climates. This finding aligns with previous reports (Bauza et al., 2019; Hagedorn et al., 2011; Somnark et al., 2018) that emphasize the lack of MST marker validation in the Global South.

Although MST represents a significant advancement over traditional FIOs, such as *E. coli*, it remains an imperfect solution for accurately assessing human and environmental health risks. Traditional FIOs are limited to indicating the presence of fecal contamination but fail to provide information about its source (Henry et al., 2016; Horan, 2003). MST addresses this gap by identifying the origin of contamination, making it a valuable starting point. However, it does not provide direct insights into exposure pathways, microbial activity, infectivity, or pathogenicity. As such, to truly assess risks, there is a need for complementary culture-based strategies that can detect the presence of viable pathogens (Ahmed et al., 2021; Harwood et al., 2014; Zhang et al., 2019).

To address these limitations, it is essential to develop an integrated MST framework that combines molecular methods with culture-based approaches that more accurately reflect health risks. In the interim, MST remains an important step forward compared with traditional FIOs, while highlighting the need for continued research and methodological advancements.

### Future directions

Artificial intelligence (AI) and machine learning (ML) are increasingly being utilized in MST, offering exciting opportunities to advance the field. These technologies first gained traction with Bayesian approaches, exemplified by the development of SourceTracker software, which has been widely applied for probabilistic source attribution (Knights et al., 2011). As the field evolves, random forest models are emerging as the next prominent approach, providing enhanced capabilities for analyzing large and complex datasets (Belanche-Muñoz & Blanch, 2008; Dubinsky et al., 2016; Roguet et al., 2018; Smith et al., 2010; Wu et al., 2020). Despite these advancements, there is still no standard method or comprehensive comparison between these tools, reflecting the infancy of ML and AI applications in MST.

Nevertheless, the potential of these technologies is undeniable, particularly for handling very large genomic data sets, which are often vast and intricate. As the field progresses, AI and ML are expected to play a pivotal role in improving marker selection, performance evaluation, and data interpretation. While current applications remain exploratory, the rapid pace of development signals a promising future for integrating these cutting-edge tools into MST methodologies (Mathai et al., 2020).

Additionally, future genomic methods may enhance MST capabilities by detecting virulence genes, offering critical insights into pathogenicity. However, without complementary culture-based approaches, challenges remain in determining viable pathogen prevalence (dose) and therefore the corresponding health risks. Investigating an integrated framework that combines molecular methods, genomic data, and culture-based techniques should be a priority to ensure comprehensive and accurate risk assessments (Raza et al., 2021).

While this review focuses on human-specific MST markers, there is a growing body of research exploring animal-specific fecal markers (Bernhard & Field, 2000; Harwood et al., 2014; Hussein et al., 2014; Lu et al., 2008; Mieszkin et al., 2009; Shanks et al., 2008). However, these markers often exhibit similar limitations in sensitivity and specificity as those observed with human markers (Harwood et al., 2014). Variability in marker performance across regions and environmental conditions underscores the need for rigorous validation efforts. Therefore, the recommendations made in this paper for human-specific markers—such as regionally tailored validation, standardization of methodologies, and improvements in sensitivity and specificity—should also be applied to animal-specific markers (McLellan & Eren, 2014). Ensuring robust marker performance across species and geographic contexts will enhance the global applicability of MST methods,

contributing to more effective and comprehensive risk assessments for both human and animal fecal contamination.This is particularly important given the current lack of standardized MST protocols, with the notable exception of HF183, which has recently benefited from the publication of standard protocols by the EPA (U.S. EPA, 2010, 2019). The absence of standardization hinders the comparability of results across studies, regions, and species, potentially exacerbating disparities in data quality between HICs and LMICs. Such inconsistencies further complicate global MST efforts. Therefore, the development and adoption of standardized MST methods are critical for ensuring consistency and facilitating meaningful cross-regional and cross-species comparisons (Hagedorn et al., 2011).

To further address disparities between HICs and LMICs, we suggest prioritizing library-independent MST approaches as an initial step. These approaches strike a middle ground between the expensive and resource-intensive library-dependent MST methods and traditional FIOs, such as *E. coli* (Ahmed, 2007; Holcomb & Stewart, 2020). While FIOs are low-cost, they lack the specificity to identify the source of fecal contamination, making them less effective for targeted interventions. Library-independent MST, by contrast, offers a more accessible and cost-effective option while providing greater accuracy and specificity than traditional FIOs (Field et al., 2003; Hagedorn et al., 2011; Meals et al., 2013).

These constraints underscore the urgent need for regionally tailored MST solutions that balance accessibility with accuracy. MST methods must be developed, validated, and standardized for use in diverse settings, particularly in LMICs and other underrepresented regions, ensuring that they are effective in the contexts where they are most needed. Addressing these challenges is essential to unlock the full potential of MST as a tool for improving water quality and public health globally.

## Conclusion

This review underscores the critical finding that no single marker is globally sufficient, with all 13 markers exhibiting varying performance across different geographic regions. Given the profound influence of local cultures, climates, and environmental conditions on the human gut microbiome, it is imperative that marker efficacy be verified within specific geographic contexts before substantial time, money, and resources are allocated to research.

Our analyses provide a robust foundation for the preliminary selection of MST markers, utilizing comprehensive decision-making tools tailored to diverse regions. However, the global applicability of MST as a tool for enhancing human health risk detection and informing targeted mitigation strategies is severely limited by the current research gaps across various geographic settings. This limitation underscores the necessity for expanded research efforts across a broader spectrum of regions. Where resources allow, the use of library-dependent methods is recommended to identify regionally specific markers, which can then be developed for use with library-independent approaches. Without such efforts, the potential of MST to serve as a powerful tool in global public health remains underutilized.

Furthermore, global recommendations, such as those from the World Health Organization, must account for the stark disparities between regions, particularly between HICs and LMICs. Failure to address this divide perpetuates the use of inappropriate markers, especially in regions burdened by diarrheal diseases, where the correct application of thoroughly researched MST markers could significantly improve health outcomes. To achieve this, standardized MST methods must be established, ensuring consistency and reliability in identifying sources of fecal contamination globally, thereby enhancing the effectiveness of interventions and policies aimed at improving public health globally.

## Supplementary Material

Supplamenatry_Material.docx

## Data Availability

The data that support the findings of this study are available from the corresponding author, Rebekah Henry, upon reasonable request.
