# Supplementary material for: Beyond borders: A systematic review and meta-analysis of human-specific faecal markers across geographical settings
Source: Crit Rev Environ Sci Technol. 2025 Feb 6;55(7):447–64. doi: 10.1080/10643389.2025.2455031 (PMC12051442; doi:10.1080/10643389.2025.2455031)
Supplement: Supplamenatry_Material.docx [file BEST_A_2455031_SM4515.docx]

Contents

[**Supplementary Material 1.0 – Preliminary scoping and umbrella review** 2](#_Toc179477089)

[**Rational** 2](#_Toc179477090)

[**Search Strategy** 3](#_Toc179477091)

[**Results** 6](#_Toc179477092)

[**Supplementary Material 2.0 – Systematic Review Methodology** 12](#_Toc179477093)

[**Eligibility Criteria** 13](#_Toc179477094)

[**Secondary Screening Guidelines** 14](#_Toc179477095)

[**Supplementary Material 3.0 – Systematic Review and Meta-Analysis Search Results** 16](#_Toc179477096)

[**Supplementary Material 4.0 – Systematic Review and Meta-Analysis Analysis Results** 76](#_Toc179477097)

# **Supplementary Material 1.0 – Preliminary scoping and umbrella review**

## **Rational**

To identify those human-specific markers appropriate for inclusion in this review, two preliminary reviews were conducted. An umbrella review; where existing reviews of microbial source tracking markers were targeted using the search terms outlined in Table S1.1, and a scoping review of human-specific microbial source racking methods that have been used in regions of the Global South using search terms outlined in Table S1.2. The inclusion and exclusion criteria for each of these studies can be seen in Table S1.3. Each of the results then underwent a title and abstract screening and if they passed this step a full text screening before data was extracted for meta-analysis. In this meta-analysis average sensitivity and specificity of each marker was calculated based on the paper it was reported in and this was then combined with all other reports from additional papers included, any marker that showed an average sensitivity and specificity of 80% or higher was included within the published systematic review.^1^ The numerical results of each screening stage can be seen in Figure S1.1 and S1.2, while the final papers included are outline in Tables S1.4 and S1.5, with results from the meta-analysis outlines in Tables S1.6 and S1.7. In total 10 relevant papers were identified from the preliminary umbrella review and 22 relevant papers from the preliminary scoping review. From these 80 human-specific markers were identified and of these 14 (this was later reduced to 13 as a result of the lack of geographic location specification for HumM3 in the papers identified within the Systematic Review) met the 80% average sensitivity and specificity criteria, 5 of which were identified through both the preliminary umbrella and scoping review.

## **Search Strategy**

**Table S1.1**: Search terms used for the Umbrella Review preliminary search and the number of results returned from PubMed, Scopus and Web of Science on the 23^rd^ May 2023.

| **Database** | **Search Terms** | **Number of Results** |
| --- | --- | --- |
| **PubMed** | ("microbial source tracking"[Title/Abstract] OR "Source tracking"[Title/Abstract] OR "faecal source tracking" [Title/Abstract] OR "fecal source tracking" [Title/Abstract]) AND ("marker"[Title/Abstract] OR "biomarker"[Title/Abstract] OR "microbial marker"[Title/Abstract] OR "viral marker"[Title/Abstract] OR “faecal marker” [Title/Abstract] OR “fecal marker” [Title/Abstract] OR “human marker”[Title/Abstract]) AND ("human"[Title/Abstract] OR "human specific"[Title/Abstract] OR "sewage"[Title/Abstract] OR "sewerage"[Title/Abstract] OR "wastewater"[Title/Abstract]) OR ("human"[Title/Abstract] AND "Faecal"[Title/Abstract] OR "Fecal"[Title/Abstract] OR "Faeces"[Title/Abstract] OR "Feces"[Title/Abstract]) AND ("Sensitivity"[Title/Abstract] AND "Specificity"[Title/Abstract])  Review OR Systematic Review | 268 |
| **Scopus** | (TITLE-ABS-KEY-AUTH(“microbial source tracking”) OR (“source tracking”) OR (“faecal source tracking”) OR (“fecal source tracking”) AND (marker) OR (biomarker) OR (“microbial marker”) OR (“viral marker”) OR (“faecal marker”) OR (“fecal marker”) OR (“human marker”) AND (human) OR (“human specific”) OR (sewage) OR (sewerage) OR (wastewater) OR (human) AND (faecal) OR (fecal) OR (faeces) OR (feces) AND (sensitivity) AND (specificity)) ( LIMIT-TO ( DOCTYPE,"re" ) ) | 62 |
| **Web of Science** | TS=(("microbial source tracking" OR "source tracking" OR “faecal source tracking” OR “fecal source tracking”) AND ("marker" OR "biomarker" OR "viral marker" OR "microbial marker" OR “faecal marker” OR “fecal marker” OR “human marker”) AND ("human" OR "human specific" OR "sewage" OR "sewerage" OR "wastewater") OR ("human" AND "faecal" OR "fecal" OR "faeces" OR "feces") AND ("sensitivity" AND "specificity"))  Review Article | 264 |

**Table S1.2**: Search terms used for the Scoping Review preliminary search and the number of results returned from PubMed, Scopus and Web of Science on the 22nd May 2023.

| **Database** | **Search Terms** | **Number of Results** |
| --- | --- | --- |
| **PubMed** | ("microbial source tracking"[Title/Abstract] OR "Source tracking"[Title/Abstract] OR "faecal source tracking" [Title/Abstract] OR "fecal source tracking" [Title/Abstract]) AND ("marker"[Title/Abstract] OR "biomarker"[Title/Abstract] OR "microbial marker"[Title/Abstract] OR "viral marker"[Title/Abstract] OR “faecal marker” [Title/Abstract] OR “fecal marker” [Title/Abstract] OR “human marker”[Title/Abstract]) AND ("human"[Title/Abstract] OR "human specific"[Title/Abstract] OR "sewage"[Title/Abstract] OR "sewerage"[Title/Abstract] OR "wastewater"[Title/Abstract]) OR ("human"[Title/Abstract] AND "Faecal"[Title/Abstract] OR "Fecal"[Title/Abstract] OR "Faeces"[Title/Abstract] OR "Feces"[Title/Abstract]) AND ("Sensitivity"[Title/Abstract] AND "Specificity"[Title/Abstract]) AND ("Informal Settlement"[Title/Abstract] OR "Slum"[Title/Abstract] OR "Shanty Town"[Title/Abstract] OR "Third World"[Title/Abstract] OR "Global South"[Title/Abstract] OR "Less Economically Developed Countries"[Title/Abstract] OR "LEDC"[Title/Abstract] OR "Low and Middle Income Countries"[Title/Abstract] OR "LMIC"[Title/Abstract] OR "Africa"[Title/Abstract] OR "Asia"[Title/Abstract] OR "Latin America"[Title/Abstract] OR "South America"[Title/Abstract] OR "Oceania"[Title/Abstract] OR "Pacific Island"[Title/Abstract]) NOT “Chemical” [Title/Abstract] | 55 |
| **Scopus** | (TITLE-ABS-KEY-AUTH(“microbial source tracking”) OR (“source tracking”) OR (“faecal source tracking”) OR (“fecal source tracking”) AND (marker) OR (biomarker) OR (“microbial marker”) OR (“viral marker”) OR (“faecal marker”) OR (“fecal marker”) OR (“human marker”) AND (human) OR (“human specific”) OR (sewage) OR (sewerage) OR (wastewater) OR (human) AND (faecal) OR (fecal) OR (faeces) OR (feces) AND (sensitivity) AND (specificity) AND (“informal settlement”) OR (slum) OR (“shanty town”) OR (“third world”) OR (“global south”) OR (“Less economically developed countries”) OR (ledc) OR (“low- and middle-income countries”) OR (lmic) OR (Africa) OR (Asia) OR (“Latin America”) OR (“South America”) OR (Oceania) OR (“Pacific Island”)) | 94 |
| **Web of Science** | TS=(("microbial source tracking" OR "source tracking" OR “faecal source tracking” OR “fecal source tracking”) AND ("marker" OR "biomarker" OR "viral marker" OR "microbial marker" OR “faecal marker” OR “fecal marker” OR “human marker”) AND ("human" OR "human specific" OR "sewage" OR "sewerage" OR "wastewater") OR ("human" AND "faecal" OR "fecal" OR "faeces" OR "feces") AND ("sensitivity" AND "specificity") AND (“informal settlement” OR “slum” OR “shanty town” OR “third world” OR “global south” OR “Less economically developed countries” OR “LEDC” OR “low- and middle-income countries” OR “LMIC” OR “Africa” OR “Asia” OR “Latin America” OR “South America” OR “Oceania” OR “Pacific Island”)) | 471 |

**Table S1.3**: The inclusion and exclusion criteria used for the preliminary searches used to inform this review.

| **Review** | **Inclusion Criteria** | **Exclusion Criteria** |
| --- | --- | --- |
| **Umbrella Review** | A review of at least 1 human-specific microbial source tracking marker with the reported sensitivity and specificity of studies reviewed included | A review of animal specific microbial source tracking markers  A review not encompassing microbial source tracking  A review of library dependent microbial source tracking methods  A review not in English  A review not reporting sensitivity or specificity |
| **Scoping Review** | A study investigating at least 1 human-specific microbial source tracking marker with the reported sensitivity and specificity included  This study must have taken place in a region of the Global South | A study of animal specific microbial source tracking markers  A study not encompassing microbial source tracking  A study of library dependent microbial source tracking methods  A study not in English  A review Paper  A study taking place in Global North  A study not reporting sensitivity or specificity |

## **Results**

Manual Search

0

**10**

Irrelevant

26

**36**

Irrelevant

398

Duplicates

160

**594**

Web of Science

264

Pub Med

268

Scopus

62

**Figure S1.1**: Breakdown of the screening for the preliminary umbrella review.

Manual Search

1

**22**

Irrelevant

75

**36**

Irrelevant

444

Duplicates

80

**620**

Web of Science

471

Pub Med

55

Scopus

94

**Figure S1.2**: Breakdown of the screening for the preliminary scoping review.

**Table S1.4:** Summary of the 10 papers identified through the preliminary umbrella review.

| **Author** | **Year** | **Title** | **Journal** | **Markers Reviewed** |
| --- | --- | --- | --- | --- |
| Amanda B Herzog | 2010 | Detection and occurrence of indicator organisms and pathogens | Water Environment Research | BacHum-UCD,  Bacteroides HF183,  Bifidobacterium catenulatum,  Faecalibacterium,  Human Polyomavirus, Bacteroidales,  Methanobrevibacter smithii |
| Andri T Rachmadi | 2016 | Human polyomavirus: Advantages and limitations as a human-specific viral marker in aquatic environments | Water Research | Human Polyomavirus - (JCV + BKV),  Human Polyomavirus – BKV,  Human Polyomavirus – JCV,  Human Polyomavirus – MCPYV |
| Enze Li | 2021 | Biological indicators for fecal pollution detection and source tracking: A review | Processes | CrAssphage,  Human Adenovirus,  Human Polyomavirus, |
| Erin M Symonds | 2018 | Pepper mild mottle virus: A plant pathogen with a greater purpose in (waste)water treatment development and public health management. | Water Research | Pepper Mild Mottle Virus |
| Kelvin Wong | 2012 | Application of enteric viruses for fecal pollution source tracking in environmental waters. | Environment International | Hepatitis E Virus,  Human Adenovirus,  Human Adenovirus – C,  Human Adenovirus -F,  Human Polyomavirus,  Norovirus GI,  Norovirus GII,  Norovirus GIII,  Oncolytic Adenovirus, |
| Muhammad Adnan Sabar | 2022 | CrAssphage as an indicator of human-fecal contamination in water environment and virus reduction in wastewater treatment | Water Research | CrAssphage |
| Tatsuya Unno | 2018 | Fecal pollution: new trends and challenges in microbial source tracking using next-generation sequencing | Environmental Microbiology | Bacteroides HF183 |
| Temitope C Ekundayo | 2021 | Prevalence of emerging torque teno virus (TTV) in drinking water, natural waters and wastewater networks (DWNWWS): A systematic review and meta-analysis of the viral pollution marker of faecal and anthropocentric contaminations | Science of the Total Environment | Torque Teno Virus (TTV) |
| Valerie J Harwood | 2014 | Microbial source tracking markers for detection of fecal contamination in environmental waters: Relationships between pathogens and human health outcomes | Microbiology Reviews | BacHuman, BacHum-UCD,  Bacteroidales,  Bacteroides thetaiotamicron,  Bacteroides fragilis F1, Bifidobacterium,  Bacteroides stercoris F1,  Bacteroides uniformis F2,  Bacteroides vulgatus F1,  Enterococcus,  HuBac,  Human Adenovirus – C,  Human Polyomavirus,  Human Polyomavirus JC and BK,  HumM2, HumM3,  Methanobrevibacter smithii,  Norovirus GI,  Norovirus GII,  PcoprilF1,  Pepper Mild Mottle Virus |
| Warish Ahmed | 2016 | Current status of marker genes of bacteroides and related taxa for identifying sewage pollution in environmental waters | Water | BacH  BacHum-UCD  Bacteroides HF134  Bacteroides HF183  Bacteroides thetaiotamicron  HuBac  HumanBac1  HumM2  HumM3 |

**Table S1. 5:** Summary of the 22 papers identified through the preliminary scoping review.

| **Author** | **Date** | **Title** | **Journal** | **Markers** |
| --- | --- | --- | --- | --- |
| Haiyang Chen | 2021 | Integrating Metagenomic and Bayesian Analyses to Evaluate the performance and confidence of CrAssphage as an Indicator for Tracking Human Sewage Contamination in China | Environmental Science & Technology | CrAssphage,  Human Adenovirus F,  Human Polyomavirus BK,  Human Polyomavirus JC,  Human Polyomavirus KI,  Human Polyomavirus MC,  Human Polyomavirus WU,  Torque Teno Virus,  ΦB124-14,  ΦCrAss001 |
| Shin Giek Goh | 2021 | Assessment of Human Health Risks in Tropical Environmental Waters with Microbial Source Tracking Markers | Water Research | Bacteroides thetaiotamicron,  Human Polyomavirus,  Methanobrevibacter smithii |
| Angela R Harris | 2016 | Ruminants Contribute Faecal Contamination to the Urban Household Environment in Dhaka, Bangladesh | Environmental Science & Technology | BacHum,  Bacteroides HF183,  HumM2 |
| David A Holcomb | 2020 | Human fecal contamination of water, soil and surfaces in households sharing poor-quality sanitation facilities in Maputo, Mozambique | International Journal of Hygiene and Environmental Health | BacHum,  Bacteroides HF183,  Human Adenovirus,  mNIF |
| Marion W Jenkins | 2009 | Identifying human and livestock sources of fecal contamination in Kenya with host-specific Bacteroidales assays | Water Research | Bacteroides HF183 |
| Renuka Kapoor | 2021 | Evaluation of Low-Cost Phage-Based Microbial Source Tracking Tools for Elucidating Human Fecal Contamination Pathways in Kolkata, India | Frontiers in Microbiology | ASH-08,  GB-124,  WG-5 |
| Akechai Kongprajug | 2019 | CrAssphage as a Potential Human Sewage Marker for Microbial Source Tracking in Southeast Asia | Environmental Science & Technology | Bacteroides HF183,  CrAssphage |
| Rita Linke | 2020 | Assessing the faecal source sensitivity and specificity of ruminant and human genetic microbial source tracking markers in the central Ethiopian highlands | Letters in Applied Microbiology | BacHum,  Bacteroides HF183 |
| Xiang Li | 2022 | Developing a novel Bifidobacterium phage quantitative polymerase chain reaction-based assay for tracking untreated wastewater | Science of the Total Environment | Bifidobacterium,  CPQ_56,  HF183 SYBR |
| Bikash Malla | 2018 | Validation of host-specific Bacteroidales quantitative PCR assays and their application to microbial source tracking of drinking water sources in the Kathmandu Valley, Nepal | Journal of Applied Microbiology | BacHum,  HF183 SYBR,  HF183 TaqMan |
| Bikash Malla | 2019 | Performance Evaluation of Human‑Specific Viral Markers and Application of Pepper Mild Mottle Virus and CrAssphage to Environmental Water Samples as Fecal Pollution Markers in the Kathmandu Valley, Nepal | Food and Environmental Virology | Aichi Virus,  CrAssphage,  Human Adenovirus,  Human Polyomavirus BK,  Human Polyomavirus JC,  Pepper Mild Mottle Virus |
| Pimchanok Nopprapun | 2020 | Evaluation of a human-associated genetic marker for *Escherichia coli* (H8) for fecal source tracking in Thailand | Water Science & Technology | *E. coli H8* |
| Jean Pierre Nshimyimana | 2017 | Bacteroidales markers for microbial source tracking in Southeast Asia | Water Research | BacH, BacHum,  Bacteroides HF183,  Bacteroides thetaiotamicron,  HF183 SYBR |
| Mitsunori Odagiri | 2015 | Validation of Bacteroidales quantitative PCR assays targeting human and animal fecal contamination in the public and domestic domains in India | Science of the Total Environment | BacH,  BacHum,  HF183 SYBR,  HF183 TaqMan,  HumM2 |
| Watsawan Sangkaewa | 2021 | Performance of viral and bacterial genetic markers for sewage pollution tracking in tropical Thailand | Water Research | BacV6-21,  CrAssphage,  Human Polyomavirus,  Lachno 3 |
| Francesca Schiaffino | 2020 | Validation of microbial source tracking markers for the attribution of fecal contamination in indoor-household environments of the Peruvian Amazon | Science of the Total Environment | BacHum,  Bacteroides HF183 |
| Pornjira Somnark | 2018 | Performance evaluation of Bacteroidales genetic markers for human and animal microbial source tracking in tropical agricultural watersheds | Environmental Pollution | BacHum,  HF183/Bac708R,  HF183/BFDrev EP |
| Erin M Symonds | 2017 | Microbial source tracking in shellfish harvesting waters in the Gulf of Nicoya, Costa Rica | Water Research | Bacteroides HF183,  Human Polyomavirus,  Pepper Mild Mottle Virus |
| Kiran Kumar Vadde | 2019 | Quantification of Microbial Source Tracking and Pathogenic Bacterial Markers in Water and Sediments of Tiaoxi River (Taihu Watershed) | Frontiers in Microbiology | BacHum, HF183 SYBR,  HF183 TaqMan, Hum2 |
| Bencharong Wangkahad | 2016 | Integrated Multivariate Analysis with Nondetects for the Development of Human Sewage Source-Tracking Tools Using Bacteriophages of Enterococcus faecalis | Environmental Science & Technology | Enterococcus |
| Yang Zhang | 2020 | Performance of host-associated genetic markers for microbial source tracking in China | Water Research | BacH, BacHum,  CPQ_056, CPQ_064,  HF183 SYBR,  Hum163, Hum2 |
| Kevin Zhu | 2020 | A novel droplet digital PCR human mtDNA assay for fecal source tracking | Water Research | Bacteroides HF183 |

**Table S1.6**: Average sensitivity and specificity of the human-specific markers that met the 80% threshold identified from the preliminary umbrella review.

| **Marker** | **Average Sensitivity (%)** | **Average Specificity (%)** |
| --- | --- | --- |
| **Bacteroides thetaiotamicron** | 96.14 | 94.00 |
| **BacHuman** | 100.00 | 81.50 |
| ***Bacteroides HF183*** | 87.1 | 97.20 |
| **Bifidobacterium** | 94.40 | 94.50 |
| **Bifidobacterium catenulatum** | 100.00 | 87.00 |
| **Bacteroides stercoris F1** | 100 | 85.20 |
| **CrAssphage** | 97.70 | 81.78 |
| **Enterococcus** | 90.50 | 100.00 |
| **Faecalibacterium** | 80.10 | 100.00 |
| **Human Adenovirus** | 81.60 | 100.00 |
| **HumM2** | 89.30 | 98.80 |
| **HumM3** | 100.00 | 96.90 |
| **Methanobrevibacter smithii** | 100.00 | 100.00 |

**Table S1.7:** Average sensitivity and specificity of the human-specific markers that met the 80% threshold identified from the preliminary scoping review.

| **Marker** | **Average Sensitivity (%)** | **Average Specificity (%)** |
| --- | --- | --- |
| **Bacteroides thetaiotamicron** | 84.50 | 93.00 |
| **Bifidobacterium** | 100.00 | 100.00 |
| **Enterococcus** | 89.15 | 88.65 |
| **Human Polyomavirus** | 95.20 | 100.00 |
| **Methanobrevibacter smithii** | 100.00 | 97.00 |

# **Supplementary Material 2.0 – Systematic Review Methodology**

**Table S2.1**: Search terms used for the Systematic review of Human Polyomavirus (HPyV) in PubMed, Scopus and Web of Science.

| **Database** | **Search Terms** |
| --- | --- |
| **PubMed** | (“Human Polyomavirus” [Text Word] OR “HPyV” [Text Word]) AND ("microbial source tracking"[Title/Abstract] OR "Source tracking"[Title/Abstract] OR "faecal source tracking" [Title/Abstract] OR "fecal source tracking" [Title/Abstract]) AND (“sensitivity” [Text Word] OR “specificity” [Text Word] OR “accuracy” [Text Word]) |
| **Scopus** | (ALL (“Human Polyomavirus”) OR (“HPyV”)) AND (TITLE-ABS-KEY (“microbial source tracking”) OR (“source tracking”) OR (“faecal source tracking”) OR (“fecal source tracking”)) AND (ALL(“sensitivity”) OR (“specificity”) OR (“accuracy”)) |
| **Web of Science** | (ALL= (Human Polyomavirus) OR ALL=(HPyV)) AND (TS= (microbial source tracking) OR TS= (source tracking) OR TS= (faecal source tracking) OR TS= (fecal source tracking)) AND (ALL=(sensitivity) OR ALL=(specificity) OR ALL=(accuracy)) |

## **Eligibility Criteria**

**Inclusion criteria**

- The study must provide a quantifiable value for either sensitivity (tested against human faecal waste), specificity (tested against a non-human species faecal waste) or accuracy of the marker.
- The paper must state the geographic area which human faecal samples that were tested were collected from - if the paper meets the criteria for a quantifiable performance value but not the geographic region then please note this as we will contact the author for clarification.
- Publications in all languages can be included
- The search targets all research types. We only want to target original research so if you come across a review paper please note this and click maybe. Once in full text screening or screen 2 you will read this paper to find the reference for the original research for the relevant marker. You will then manually add this paper in and mark the review as ineligible.

**Exclusion criteria**

- Any studies that use the marker to understand human or environmental health risks but do not comment on the performance of the marker (sensitivity, specificity or accuracy) should be excluded
- Any studies were the human-specific faecal marker is tested for sensitivity against a non-human source should be excluded
- REVIEWS: The search targets all research types. We only want to target original research so if you come across a review paper please note this and click maybe. Once in full text screening or screen 2 you will read this paper to find the reference for the original research for the relevant marker. You will then manually add this paper in and mark the review as ineligible.
- BOOK CHAPTERS: though I have tried my hardest to remove duplicates, some book chapters are published multiple times in different book versions - if you find a duplicate please only consider the most recently published version and mark all others as duplicates.

## **Secondary Screening Guidelines**

**In the second screening you will have the choice to include or exclude if you choose exclude the following options will appear:**

- Generally irrelevant
- Review paper - primary research has been extracted
- Full text unavailable
- No quantifiable sensitivity, specificity or accuracy
- No geographic location
- Geographic location is not distinguished between samples
- Other - Please leave note

**Generally irrelevant**

In this case if the text has no mention of the marker at all then you can mark it as generally irrelevant

**Review paper - primary research has been extracted**

If the review paper has no mention of the specific marker then you can mark it as “Generally irrelevant”. If it has the marker but know validation statistics (specificity, sensitivity or accuracy) then you can mark it as “No quantifiable sensitivity, specificity or accuracy”

If there is a mention of the specific marker and it has the statistics required then in the notes mark that it is a review. Read through the results and data it presents and extract the full reference for the primary data set. Please place these in the notes for later upload.

Once this is done you can mark it as excluded and “Review paper - primary research has been extracted”

**Full text unavailable**

Where text is unavailable online the author will be contacted. If it has reached 01/06/2024 and no text has been provided then you can mark it as full text unavailable.

**No quantifiable sensitivity, specificity or accuracy**

When you read through the paper you are looking for an indication of at least one of these statistical values - sensitivity, specificity or accuracy or equivalent. If none are indicated you may exclude the paper with this reasoning.

**No geographic location**

If the paper mentions the marker and has the sensitivity, specificity and accuracy then consider this criterion. Initially please leave a note saying no geographic location the author will then be contacted to allow for clarification. If we do not get a response by 01/06/2024 it will be excluded with this criterion.

**Geographic location is not distinguished between samples**

If the paper mentions multiple geographic locations that are in different climate zones, continents or classed as differing development status but does not provide performance statics grouped via those differences.

**Other - Please leave note**

Finally, if there is any other reason that you believe this paper doesn’t meet the necessary criteria then leave a note and pick this option

**Figure S2. 1**: Data extraction and risk of bias assessment template.

# **Supplementary Material 3.0 – Systematic Review and Meta-Analysis Search Results**

**Table S3.1:** List of studies excluded at the full text screening stage, which of the markers it was identified for and the reason for exclusion.

| **Title** | **Author** | **Year** | **Marker** | **Reason for Exclusion** | **Note** |
| --- | --- | --- | --- | --- | --- |
| A new microbial source tracking strategy using bacteroides 16S rRNA signatures in water sources | Abbaszadegan, M.; Kabiri, L.; Alum, A. | 2012 | *Bacteroides HF183* | Full text unavailable |  |
| A Novel Microbial Source Tracking Microarray for Pathogen Detection and Fecal Source Identification in Environmental Systems | Li, X.; Harwood, V.J.; Nayak, B.; Staley, C.; Sadowsky, M.J.; Weidhaas, J. | 2015 | *Enterococcus* | No quantifiable sensitivity, specificity or accuracy |  |
| A real-time polymerase chain reaction assay for quantitative detection of the human-specific enterococci surface protein marker in sewage and environmental waters | Ahmed, W.; Stewart, J.; Gardner, T.; Powell, D. | 2008 | *Enterococcus* | Other - Please leave note | Uses the same samples/results from another article |
| A real-time qPCR assay for the detection of the nifH gene of Methanobrevibacter smithii, a potential indicator of sewage pollution | Johnston, C.; Ufnar, J.A.; Griffith, J.F.; Gooch, J.A.; Stewart, J.R. | 2010 | *Methanobrevibacter smithii* | No quantifiable sensitivity, specificity or accuracy |  |
| A review on microbial contaminants in stormwater runoff and outfalls: Potential health risks and mitigation strategies | Ahmed, W.; Hamilton, K.; Toze, S.; Cook, S.; Page, D. | 2019 | *Human Adenovirus* | Review paper - primary research has been extracted |  |
| A tool box strategy using Bacteroides genetic markers to differentiate human from non-human sources of fecal contamination in natural water | Kabiri, L.; Alum, A.; Rock, C.; McLain, J.E.; Abbaszadegan, M. | 2016 | *Bacteroides HF183* | No quantifiable sensitivity, specificity or accuracy |  |
| Advances in microbial source tracking methods | Zheng, Q.-X.; Zhang, Y.; Yu, X.-W.; Wei, S.-Y.; Huang, J.-H.; Wu, R.-R. | 2021 | *CrAssphage, Human Adenovirus, Enterococcus* | Full text unavailable |  |
| Alternative indicators of fecal pollution: Relations with pathogens and conventional indicators, current methodologies for direct pathogen monitoring and future application perspectives | Savichtcheva, O.; Okabe, S. | 2006 | *Enterococcus* | Review paper - primary research has been extracted |  |
| An assessment of three methods for extracting bacterial DNA from beach sand. | Gallard-Gongora J; Lobos A; Conrad JW; Peraud J; Harwood VJ | 2022 | *Bacteroides HF183* | No quantifiable sensitivity, specificity or accuracy |  |
| Analysis of human and animal fecal microbiota for microbial source tracking | Lee, J.E.; Lee, S.; Sung, J.; Ko, G. | 2011 | *Bacteroides HF183, Bifidobacterium* | Generally irrelevant, No quantifiable sensitivity, specificity or accuracy |  |
| Analytical pollution source tracking: Methods and experiences feedback about the identification of pollution causes on bathing waters, in France and UK | Gourmelon, M.; Caprais, M.R.; Kay, D.; Stapleton, C. | 2010 | *Bacteroides HF183* | Full text unavailable |  |
| Applicability of F-specific bacteriophage subgroups, PMMoV and crAssphage as indicators of source specific fecal contamination and viral inactivation in rivers in Japan | Meuchi, Y.; Nakada, M.; Kuroda, K.; Hanamoto, S.; Hata, A. | 2023 | *CrAssphage* | No quantifiable sensitivity, specificity or accuracy |  |
| Application of an integrated community analysis approach for microbial source tracking in a coastal creek. | Cao Y; Van De Werfhorst LC; Sercu B; Murray JL; Holden PA | 2011 | *Enterococcus* | Generally irrelevant |  |
| Application of enteric viruses for fecal pollution source tracking in environmental waters. | Wong K; Fong TT; Bibby K; Molina M | 2012 | *Human Polyomavirus, Human Adenovirus* | Review paper - primary research has been extracted |  |
| Application of Faecalibacterium as index bacteria of feces in water | Duan, C.; Liu, A.; Zheng, G.; Wang, G.; Gao, X.; Guo, J.; Sun, D.; Nie, L. | 2013 | *Faecalibacterium* | Full text unavailable |  |
| Application of human and animal viral microbial source tracking tools in fresh and marine waters from five different geographical areas. | Rusiñol M; Fernandez-Cassi X; Hundesa A; Vieira C; Kern A; Eriksson I; Ziros P; Kay D; Miagostovich M; Vargha M; Allard A; Vantarakis A; Wyn-Jones P; Bofill-Mas S; Girones R | 2014 | *Human Adenovirus* | Generally irrelevant |  |
| Assessment of Human Health Risks in Tropical Environmental Waters with Microbial Source Tracking Markers. | Goh SG; Liang L; Gin KYH | 2021 | *Enterococcus* | No quantifiable sensitivity, specificity or accuracy |  |
| Bacteriophage lysis of Enterococcus host strains: a tool for microbial source tracking? | Purnell SE; Ebdon JE; Taylor HD | 2011 | *Enterococcus* | No quantifiable sensitivity, specificity or accuracy |  |
| Bacteroides spp. as reliable marker of sewage contamination in Hawaii's environmental waters using molecular techniques | Betancourt, W.Q.; Fujioka, R.S. | 2006 | *Bacteroides thetaiotamicron, Bacteroides HF183* | Generally irrelevant, No quantifiable sensitivity, specificity or accuracy |  |
| Bifidobacterial diversity and the development of new microbial source tracking indicators. | Ballesté E; Blanch AR | 2011 | *Bifidobacterium* | No quantifiable sensitivity, specificity or accuracy |  |
| Biological indicators for fecal pollution detection and source tracking: A review | Li, E.; Saleem, F.; Edge, T.A.; Schellhorn, H.E. | 2021 | *Human Polyomavirus,* CrAssphage*, Bacteroides HF183, Human Adenovirus, Enterococcus* | Review paper - primary research has been extracted |  |
| Chapter 11 Indicators of Waterborne Enteric Viruses | Jofre, J. | 2007 | *Human Adenovirus* | Generally irrelevant |  |
| Characterization of Enterococcus spp. from human and animal feces using 16S rRNA sequences, the esp gene, and PFGE for microbial source tracking in Korea. | Kim SY; Lee JE; Lee S; Lee HT; Hur HG; Ko G | 2010 | *Enterococcus* | No quantifiable sensitivity, specificity or accuracy |  |
| Choice of indicator organism and library size considerations for phenotypic microbial source tracking by FAME profiling. | Duran M; Yurtsever D; Dunaev T | 2009 | *Enterococcus* | No quantifiable sensitivity, specificity or accuracy |  |
| Comparative assessment of human and farm animal faecal microbiota using real-time quantitative PCR | Furet, J.-P.; Firmesse, O.; Gourmelon, M.; Bridonneau, C.; Tap, J.; Mondot, S.; Doré, J.; Corthier, G. | 2009 | *Bacteroides HF183* | Generally irrelevant |  |
| Comparative assessment of human and farm animal faecal microbiota using real-time quantitative PCR. | Furet JP; Firmesse O; Gourmelon M; Bridonneau C; Tap J; Mondot S; Doré J; Corthier G | 2009 | *Bifidobacterium* | No quantifiable sensitivity, specificity or accuracy |  |
| Comparison of PCR and quantitative real-time PCR methods for the characterization of ruminant and cattle fecal pollution sources | Raith, M.R.; Kelty, C.A.; Griffith, J.F.; Schriewer, A.; Wuertz, S.; Mieszkin, S.; Gourmelon, M.; Reischer, G.H.; Farnleitner, A.H.; Ervin, J.S.; Holden, P.A.; Ebentier, D.L.; Jay, J.A.; Wang, D.; Boehm, A.B.; Aw, T.G.; Rose, J.B.; Balleste, E.; Meijer, W.G.; Sivaganesan, M.; Shanks, O.C. | 2013 | *Enterococcus* | No quantifiable sensitivity, specificity or accuracy |  |
| Cost-effective method for microbial source tracking using specific human and animal viruses | Bofill-Mas, S.; Hundesa, A.; Calgua, B.; Rusiñol, M.; de Motes, C.M.; Girones, R. | 2011 | *Human Adenovirus* | No quantifiable sensitivity, specificity or accuracy |  |
| CrAssphage as a novel tool to detect human fecal contamination on environmental surfaces and hands | Park, G.W.; Ng, T.F.F.; Freeland, A.L.; Marconi, V.C.; Boom, J.A.; Staat, M.A.; Montmayeur, A.M.; Browne, H.; Narayanan, J.; Payne, D.C.; Cardemil, C.V.; Treffiletti, A.; Vinjé, J. | 2020 | *CrAssphage* | Other - Please leave note | Regarding hand and surface health |
| CrAssphage as an indicator of human-fecal contamination in water environment and virus reduction in wastewater treatment. | Sabar MA; Honda R; Haramoto E | 2022 | *CrAssphage* | Review paper - primary research has been extracted |  |
| CrAssphage for fecal source tracking in Chile: Covariation with norovirus, HF183, and bacterial indicators. | Jennings WC; Gálvez-Arango E; Prieto AL; Boehm AB | 2020 | *CrAssphage* | No quantifiable sensitivity, specificity or accuracy |  |
| Critical issues in application of molecular methods to environmental virology | Hamza, I.A.; Bibby, K. | 2019 | *Human Adenovirus* | Review paper - primary research has been extracted |  |
| Cross-assembly phage and pepper mild mottle virus as viral water quality monitoring tools—potential, research gaps, and way forward | Bivins, A.; Crank, K.; Greaves, J.; North, D.; Wu, Z.; Bibby, K. | 2020 | *CrAssphage* | Review paper - primary research has been extracted |  |
| Current and future trends in fecal source tracking and deployment in the Lake Taihu Region of China | Hagedorn, C.; Liang, X. | 2011 | *Enterococcus* | Generally irrelevant |  |
| Current status of marker genes of bacteroides and related taxa for identifying sewage pollution in environmental waters | Ahmed, W.; Hughes, B.; Harwood, V.J. | 2016 | *Bacteroides thetaiotamicron, Bacteroides HF183, Bifidobacterium, Enterococcus, HumM3* | Review paper - primary research has been extracted, Generally irrelevant |  |
| Data acceptance criteria for standardized human-associated fecal source identification quantitative real-time PCR methods | Shanks, O.C.; Kelty, C.A.; Oshiro, R.; Haugland, R.A.; Madi, T.; Brooks, L.; Field, K.G.; Sivaganesan, M. | 2016 | *HumM2* | No quantifiable sensitivity, specificity or accuracy |  |
| Detection and occurence of indicator organisms and pathogens | Ahmad, F.; Tourlousse, D.M.; Stedtfeld, R.D.; Seyrig, G.; Herzog, A.B.; Bhaduri, P.; Hashsham, S.A. | 2009 | *Enterococcus* | No quantifiable sensitivity, specificity or accuracy |  |
| Detection and occurrence of indicator organisms and pathogens | Bhaduri, P.; Stedtfeld, R.D.; Srinivasan, S.; Kostic, T.; Herzog, A.B.; Kronlein, M.R.; Stedtfeld, T.; Liu, Y.-C.; Hashsham, S.A. | 2012 | *Bacteroides thetaiotamicron, Human Adenovirus, Enterococcus* | Generally irrelevant, Review paper - primary research has been extracted |  |
| Detection and occurrence of indicator organisms and pathogens | Kronlein, M.R.; Stedtfeld, R.D.; Sorensen, J.; Bhaduri, P.; Stedtfeld, T.; Eanes, S.; Harichandran, V.; Haynes, K.; Stevens, M.; Hashsham, S.A. | 2013 | *Bacteroides HF183, Enterococcus* | Review paper - primary research has been extracted |  |
| Detection and occurrence of indicator organisms and pathogens | Hashsham, S.A.; Alm, E.W.; Stedtfeld, R.D.; Traver, R.G.; Duran, M. | 2004 | *Bifidobacterium, Enterococcus* | Generally irrelevant, Review paper - primary research has been extracted |  |
| Detection and occurrence of indicator organisms and pathogens | Herzog, A.B.; Bhaduri, P.; Stedtfeld, R.D.; Seyrig, G.; Ahmad, F.; Dave, P.K.; Hashsham, S.A. | 2010 | *Bifidobacterium, Human Adenovirus, Enterococcus* | Review paper - primary research has been extracted |  |
| Detection and occurrence of indicator organisms and pathogens | Duran, M.; Alm, E.W.; Stedtfeld, R.D.; Haznedaroglu, B.Z.; Hashsham, S.A. | 2005 | *Bifidobacterium, Enterococcus* | Generally irrelevant, Review paper - primary research has been extracted |  |
| Detection and occurrence of indicator organisms and pathogens | Tourlousse, D.M.; Ahmad, F.; Stedtfeld, R.D.; Seyrig, G.; Duran, M.; Alm, E.W.; Hashsham, S.A. | 2008 | *Enterococcus* | Review paper - primary research has been extracted |  |
| Detection and occurrence of indicator organisms and pathogens | Samhan, F.A.; Kronlein, M.R.; Fakher, U.; Kronlein, C.; Stedtfeld, R.D.; Hashsham, S.A. | 2015 | *Enterococcus* | Review paper - primary research has been extracted |  |
| Detection of human-derived fecal pollution in environmental waters by use of a PCR-based human polyomavirus assay. | McQuaig SM; Scott TM; Harwood VJ; Farrah SR; Lukasik JO | 2006 | *Enterococcus* | No quantifiable sensitivity, specificity or accuracy |  |
| Determining sources of fecal bacteria in waterways | Yan, T.; Sadowsky, M.J. | 2007 | *Bifidobacterium, Enterococcus* | Generally irrelevant |  |
| Deterministic transition of enterotypes shapes the infant gut microbiome at an early age | Xiao, L.; Wang, J.; Zheng, J.; Li, X.; Zhao, F. | 2021 | *Bifidobacterium* | Other - Please leave note | Regarding composition of infant guts |
| Development and Integration of Quantitative Real-Time PCR Methods for Detection of Mitochondrial DNA and Methanobrevibacter smithii nifH Gene as Novel Microbial Source Tracking Tools | Dancer, D; Baker-Austin, C; Lowther, JA; Hartnell, RE; Lees, DN; Roberts, LO | 2014 | *Methanobrevibacter smithii* | No quantifiable sensitivity, specificity or accuracy |  |
| Development of a qPCR assay for the detection of naturalized wastewater E. coli strains | Zhi, S.; Banting, G.; Neumann, N.F. | 2022 | *HumM2* | No quantifiable sensitivity, specificity or accuracy |  |
| Development of a quantitative PCR assay for the quantitation of bovine polyomavirus as a microbial source-tracking tool | Hundesa, A.; Bofill-Mas, S.; Maluquer de Motes, C.; Rodriguez-Manzano, J.; Bach, A.; Casas, M.; Girones, R. | 2010 | *Human Adenovirus* | Generally irrelevant |  |
| Development of quantitative PCR assays targeting the 16s rRNA genes of enterococcus spp. and their application to the identification of enterococcus species in environmental samples | Ryu, H.; Henson, M.; Elk, M.; Toledo-Hernandez, C.; Griffith, J.; Blackwood, D.; Noble, R.; Gourmelon, M.; Glassmeyer, S.; Santo Domingo, J.W. | 2013 | *Enterococcus* | Generally irrelevant |  |
| Discovering new indicators of fecal pollution | McLellan, S.L.; Eren, A.M. | 2014 | *Bacteroides HF183* | Generally irrelevant |  |
| Distribution of human fecal marker genes and their association with pathogenic viruses in untreated wastewater determined using quantitative PCR | Ahmed, W.; Bivins, A.; Payyappat, S.; Cassidy, M.; Harrison, N.; Besley, C. | 2022 | *Bacteroides HF183* | No quantifiable sensitivity, specificity or accuracy |  |
| Distributions of fecal markers in wastewater from different climatic zones for human fecal pollution tracking in Australian surface waters | Ahmed, W.; Sidhu, J.P.S.; Smith, K.; Beale, D.J.; Gyawali, P.; Tozea, S. | 2016 | *Human Polyomavirus, Enterococcus* | No quantifiable sensitivity, specificity or accuracy |  |
| Diversity and population structure of sewage-derived microorganisms in wastewater treatment plant influent | McLellan, S.L.; Huse, S.M.; Mueller-Spitz, S.R.; Andreishcheva, E.N.; Sogin, M.L. | 2010 | *Bacteroides thetaiotamicron* | Generally irrelevant |  |
| Droplet digital PCR for simultaneous quantification of general and human-associated fecal indicators for water quality assessment. | Cao Y; Raith MR; Griffith JF | 2015 | *Bacteroides HF183, Enterococcus* | No quantifiable sensitivity, specificity or accuracy |  |
| Ecological and Technical Mechanisms for Cross-Reaction of Human Fecal Indicators with Animal Hosts. | Feng S; Ahmed W; McLellan SL | 2020 | *Enterococcus* | No quantifiable sensitivity, specificity or accuracy |  |
| Effective detection of human adenovirus in Hawaiian waters using enhanced PCR methods | Tong, H.-I.; Lu, Y. | 2011 | *Human Adenovirus* | Generally irrelevant |  |
| Enteric viruses of humans and animals in aquatic environments: Health risks, detection, and potential water quality assessment tools | Fong, T.-T.; Lipp, E.K. | 2005 | *Human Adenovirus* | Review paper - primary research has been extracted |  |
| Enterococci in the environment | Byappanahalli, M.N.; Nevers, M.B.; Korajkic, A.; Staley, Z.R.; Harwood, V.J. | 2012 | *Enterococcus* | Review paper - primary research has been extracted |  |
| Enterococci in water | Signoretto, C.; Canepari, P. | 2012 | *Enterococcus* | Full text unavailable |  |
| Evaluation of enterococcal surface protein genes as markers of sewage contamination in tropical recreational waters | Betancourt, W.Q.; Fujioka, R.S. | 2009 | *Enterococcus* | No quantifiable sensitivity, specificity or accuracy |  |
| Evaluation of Faecalibacterium 16S rDNA genetic markers for accurate identification of swine faecal waste by quantitative PCR | Duan, C.; Cui, Y.; Zhao, Y.; Zhai, J.; Zhang, B.; Zhang, K.; Sun, D.; Chen, H. | 2016 | *Faecalibacterium* | Generally irrelevant |  |
| Evaluation of four cell lines for assay of infectious adenoviruses in water samples | Jiang, S.C.; Han, J.; He, J.-W.; Chu, W. | 2009 | *Human Adenovirus* | No quantifiable sensitivity, specificity or accuracy |  |
| Evaluation of microbial indicators for the determination of bacterial groundwater contamination sources | Cimenti, M.; Biswas, N.; Bewtra, J.K.; Hubberstey, A. | 2005 | *Bifidobacterium* | No quantifiable sensitivity, specificity or accuracy |  |
| Evaluation of microbial source tracking methods using mixed fecal sources in aqueous test samples | Griffith, J.F.; Weisberg, S.B.; McGee, C.D. | 2003 | *Human Adenovirus* | No quantifiable sensitivity, specificity or accuracy |  |
| Evaluation of molecular community analysis methods for discerning fecal sources and human waste. | Cao Y; Van De Werfhorst LC; Dubinsky EA; Badgley BD; Sadowsky MJ; Andersen GL; Griffith JF; Holden PA | 2013 | *Enterococcus* | Generally irrelevant |  |
| Evaluation of quantitative PCR combined with PMA treatment for molecular assessment of microbial water quality | Gensberger, E.T.; Polt, M.; Konrad-Köszler, M.; Kinner, P.; Sessitsch, A.; Kostić, T. | 2014 | *Enterococcus* | Other - Please leave note | Regarding environemntal health |
| Evaluation of the nifH gene marker of methanobrevibacter smithii for the detection of sewage pollution in environmental waters in southeast Queensland, Australia | Ahmed, W.; Sidhu, J.P.S.; Toze, S. | 2012 | *Human Polyomavirus, Bacteroides HF183* | No quantifiable sensitivity, specificity or accuracy |  |
| Faecal pollution loads in the wastewater effluents and receiving water bodies: A potential threat to the health of Sedibeng and Soshanguve communities, South Africa | Teklehaimanot, G.Z.; Coetzee, M.A.A.; Momba, M.N.B. | 2014 | *Enterococcus* | No quantifiable sensitivity, specificity or accuracy |  |
| Faecal source tracking in seq: Case studies | Ahmed, W.; Toze, S.; Gardner, T. | 2010 | *Enterococcus* | Review paper - primary research has been extracted |  |
| Fate of viruses in water systems | Xagoraraki, I.; Yin, Z.; Svambayev, Z. | 2014 | *Human Adenovirus* | Generally irrelevant |  |
| Fecal pollution source tracking in waters intended for human supply based on archaeal and bacterial genetic markers | Bianco, K.; Barreto, C.; Oliveira, S.S.; Pinto, L.H.; Albano, R.M.; Miranda, C.C.; Clementino, M.M. | 2015 | *Bacteroides thetaiotamicron* | Generally irrelevant |  |
| Fecal pollution: new trends and challenges in microbial source tracking using next-generation sequencing | Unno, T.; Staley, C.; Brown, C.M.; Han, D.; Sadowsky, M.J.; Hur, H.-G. | 2018 | *Enterococcus* | Review paper - primary research has been extracted |  |
| Fecal source tracking in water by next-generation sequencing technologies using host-specific escherichia coli genetic markers | Gomi, R.; Matsuda, T.; Matsui, Y.; Yoneda, M. | 2014 | *Faecalibacterium* | Generally irrelevant |  |
| Fecal source tracking, the indicator paradigm, and managing water quality | Field, K.G.; Samadpour, M. | 2007 | *Human Polyomavirus, Bacteroides thetaiotamicron, Bacteroides HF183, Bifidobacterium, Human Adenovirus, Enterococcus* | Generally irrelevant, No quantifiable sensitivity, specificity or accuracy |  |
| Fidelity of bacterial source tracking: Escherichia coli vs Enterococcus spp and minimizing assignment of isolates from nonlibrary sources. | Hassan WM; Ellender RD; Wang SY | 2007 | *Enterococcus* | No quantifiable sensitivity, specificity or accuracy |  |
| Frequency of virulence genes and antibiotic resistances in Enterococcus spp. isolates from wastewater and feces of domesticated mammals and birds, and wildlife | Lanthier, M.; Scott, A.; Lapen, D.R.; Zhang, Y.; Topp, E. | 2010 | *Enterococcus* | No quantifiable sensitivity, specificity or accuracy |  |
| Genome sequencing reveals the environmental origin of enterococci and potential biomarkers for water quality monitoring | Weigand, M.R.; Ashbolt, N.J.; Konstantinidis, K.T.; Santo Domingo, J.W. | 2014 | *Enterococcus* | Generally irrelevant |  |
| Geographic sharing of ribotype patterns in Enterococcus faecalis for bacterial source tracking | Hartel, P.G.; Myoda, S.P.; Ritter, K.J.; Kuntz, R.L.; Rodgers, K.; Entry, J.A.; Ver Wey, S.A.; Schröder, E.C.; Calle, J.; Lacourt, M.; Thies, J.E.; Reilly, J.P.; Fuhrmann, J.J. | 2007 | *Enterococcus* | No quantifiable sensitivity, specificity or accuracy |  |
| Global phylogeography and ancient evolution of the widespread human gut virus crAssphage | Edwards, R.A.; Vega, A.A.; Norman, H.M.; Ohaeri, M.; Levi, K.; Dinsdale, E.A.; Cinek, O.; Aziz, R.K.; McNair, K.; Barr, J.J.; Bibby, K.; Brouns, S.J.J.; Cazares, A.; de Jonge, P.A.; Desnues, C.; Díaz Muñoz, S.L.; Fineran, P.C.; Kurilshikov, A.; Lavigne, R.; Mazankova, K.; McCarthy, D.T.; Nobrega, F.L.; Reyes Muñoz, A.; Tapia, G.; Trefault, N.; Tyakht, A.V.; Vinuesa, P.; Wagemans, J.; Zhernakova, A.; Aarestrup, F.M.; Ahmadov, G.; Alassaf, A.; Anton, J.; Asangba, A.; Billings, E.K.; Cantu, V.A.; Carlton, J.M.; Cazares, D.; Cho, G.-S.; Condeff, T.; Cortés, P.; Cranfield, M.; Cuevas, D.A.; De la Iglesia, R.; Decewicz, P.; Doane, M.P.; Dominy, N.J.; Dziewit, L.; Elwasila, B.M.; Eren, A.M.; Franz, C.; Fu, J.; Garcia-Aljaro, C.; Ghedin, E.; Gulino, K.M.; Haggerty, J.M.; Head, S.R.; Hendriksen, R.S.; Hill, C.; Hyöty, H.; Ilina, E.N.; Irwin, M.T.; Jeffries, T.C.; Jofre, J.; Junge, R.E.; Kelley, S.T.; Khan Mirzaei, M.; Kowalewski, M.; Kumaresan, D.; Leigh, S.R.; Lipson, D.; Lisitsyna, E.S.; Llagostera, M.; Maritz, J.M.; Marr, L.C.; McCann, A.; Molshanski-Mor, S.; Monteiro, S.; Moreira-Grez, B.; Morris, M.; Mugisha, L.; Muniesa, M.; Neve, H.; Nguyen, N.-P.; Nigro, O.D.; Nilsson, A.S.; O’Connell, T.; Odeh, R.; Oliver, A.; Piuri, M.; Prussin II, A.J.; Qimron, U.; Quan, Z.-X.; Rainetova, P.; Ramírez-Rojas, A.; Raya, R.; Reasor, K.; Rice, G.A.O.; Rossi, A.; Santos, R.; Shimashita, J.; Stachler, E.N.; Stene, L.C.; Strain, R.; Stumpf, R.; Torres, P.J.; Twaddle, A.; Ugochi Ibekwe, M.A.; Villagra, N.; Wandro, S.; White, B.; Whiteley, A.; Whiteson, K.L.; Wijmenga, C.; Zambrano, M.M.; Zschach, H.; Dutilh, B.E. | 2019 | *CrAssphage* | No quantifiable sensitivity, specificity or accuracy |  |
| Have genetic targets for faecal pollution diagnostics and source tracking revolutionized water quality analysis yet? | Demeter, K.; Linke, R.; Balleste, E.; Reischer, G.; Mayer, R.E.; Vierheilig, J.; Kolm, C.; Stevenson, M.E.; Derx, J.; Kirschner, A.K.T.; Sommer, R.; Shanks, O.C.; Blanch, A.R.; Rose, J.B.; Ahmed, W.; Farnleitner, A.H. | 2023 | *Bacteroides HF183* | No quantifiable sensitivity, specificity or accuracy |  |
| Highly Specific Sewage-Derived Bacteroides Quantitative PCR Assays Target Sewage-Polluted Waters. | Feng S; McLellan SL | 2019 | *Bacteroides thetaiotamicron* | Generally irrelevant |  |
| Host species-specific metabolic fingerprint database for enterococci and Escherichia coli and its application to identify sources of fecal contamination in surface waters | Ahmed, W.; Neller, R.; Katouli, M. | 2005 | *Enterococcus* | No quantifiable sensitivity, specificity or accuracy |  |
| Human Bacteroides and total coliforms as indicators of recent combined sewer overflows and rain events in urban creeks | McGinnis, S.; Spencer, S.; Firnstahl, A.; Stokdyk, J.; Borchardt, M.; McCarthy, D.T.; Murphy, H.M. | 2018 | *Human Adenovirus* | No quantifiable sensitivity, specificity or accuracy |  |
| Human-Associated Lachnospiraceae Genetic Markers Improve Detection of Fecal Pollution Sources in Urban Waters. | Feng S; Bootsma M; McLellan SL | 2018 | *Bacteroides HF183* | No quantifiable sensitivity, specificity or accuracy |  |
| Identification of Enterococcus faecium and Enterococcus faecalis as vanC-type Vancomycin-Resistant Enterococci (VRE) from sewage and river water in the provincial city of Miyazaki, Japan | Nishiyama, M.; Iguchi, A.; Suzuki, Y. | 2015 | *Enterococcus* | No quantifiable sensitivity, specificity or accuracy |  |
| Implementation and integration of microbial source tracking in a river watershed monitoring plan | Ballesté, E.; Demeter, K.; Masterson, B.; Timoneda, N.; Sala-Comorera, L.; Meijer, W.G. | 2020 | *CrAssphage* | Generally irrelevant |  |
| Improving the identification of the source of faecal pollution in water using a modelling approach: From multi-source to aged and diluted samples | Ballesté, E.; Belanche-Muñoz, L.A.; Farnleitner, A.H.; Linke, R.; Sommer, R.; Santos, R.; Monteiro, S.; Maunula, L.; Oristo, S.; Tiehm A, A.; Stange, C.; Blanch, A.R. | 2020 | *CrAssphage, Bifidobacterium, Human Adenovirus, Bacteroides HF183* | Generally irrelevant |  |
| Improving the identification of the source of faecal pollution in water using a modelling approach: From multi-source to aged and diluted samples. | Ballesté E; Belanche-Muñoz LA; Farnleitner AH; Linke R; Sommer R; Santos R; Monteiro S; Maunula L; Oristo S; Tiehm A; Stange C; Blanch AR | 2020 | *Enterococcus* | No quantifiable sensitivity, specificity or accuracy |  |
| In-field LAMP assay for rapid detection of human faecal contamination in environmental water | Khodaparast, M.; Sharley, D.; Best, N.; Marshall, S.; Beddoe, T. | 2022 | *Bacteroides HF183* | Generally irrelevant |  |
| Integrated analyses of fecal indicator bacteria, microbial source tracking markers, and pathogens for Southeast Asian beach water quality assessment. | Kongprajug A; Chyerochana N; Rattanakul S; Denpetkul T; Sangkaew W; Somnark P; Patarapongsant Y; Tomyim K; Sresung M; Mongkolsuk S; Sirikanchana K | 2021 | *Enterococcus* | No quantifiable sensitivity, specificity or accuracy |  |
| Integrating Bayesian Analysis and Cumulative Probability Generates High Confidence Using a Single Microbial Source Tracking Marker. | Curtis K; Gonzalez RA | 2019 | *HumM2, Bacteroides HF183* | Review paper - primary research has been extracted |  |
| Intraday variability of indicator and pathogenic viruses in 1-h and 24-h composite wastewater samples: Implications for wastewater-based epidemiology | Ahmed, W.; Bivins, A.; Bertsch, P.M.; Bibby, K.; Gyawali, P.; Sherchan, S.P.; Simpson, S.L.; Thomas, K.V.; Verhagen, R.; Kitajima, M.; Mueller, J.F.; Korajkic, A. | 2021 | *CrAssphage* | No quantifiable sensitivity, specificity or accuracy |  |
| Isolating the impact of septic systems on fecal pollution in streams of suburban watersheds in Georgia, United States | Sowah, R.A.; Habteselassie, M.Y.; Radcliffe, D.E.; Bauske, E.; Risse, M. | 2017 | *Bacteroides HF183* | Generally irrelevant |  |
| Isolation and characterization of pseudomonas aeruginosa and enterococcus faecalis lytic bacteriophages from wastewater for controlling multidrug resistant bacterial strains | Mahgoub, S.A.; Muhammad, M.I.S.; Abd-Elsalam, S.T.; Alkhazindar, M.M.; Abdel-Shafy, H.I. | 2020 | *Enterococcus* | No quantifiable sensitivity, specificity or accuracy |  |
| Lachnospiraceae and bacteroidales alternative fecal indicators reveal chronic human sewage contamination in an Urban harbor | Newton, R.J.; VandeWalle, J.L.; Borchardt, M.A.; Gorelick, M.H.; McLellan, S.L. | 2011 | *Bacteroides HF183* | Generally irrelevant |  |
| Lack of specificity for PCR assays targeting human Bacteroides 16S rRNA gene: Cross-amplification with fish feces | McLain, J.E.T.; Ryu, H.; Kabiri-Badr, L.; Rock, C.M.; Abbaszadegan, M. | 2009 | *Bacteroides HF183* | No quantifiable sensitivity, specificity or accuracy |  |
| Longitudinal and quantitative fecal shedding dynamics of SARS-CoV-2, pepper mild mottle virus, and crAssphage | Arts, P.J.; Kelly, J.D.; Midgley, C.M.; Anglin, K.; Lu, S.; Abedi, G.R.; Andino, R.; Bakker, K.M.; Banman, B.; Boehm, A.B.; Briggs-Hagen, M.; Brouwer, A.F.; Davidson, M.C.; Eisenberg, M.C.; Garcia-Knight, M.; Knight, S.; Peluso, M.J.; Pineda-Ramirez, J.; Sanchez, R.D.; Saydah, S.; Tassetto, M.; Martin, J.N.; Wigginton, K.R. | 2023 | *CrAssphage* | No quantifiable sensitivity, specificity or accuracy |  |
| Meta-analysis of microbial source tracking for the identification of fecal contamination in aquatic environments based on data-mining | Liu, Z.; Lin, Y.; Ge, Y.; Zhu, Z.; Yuan, J.; Yin, Q.; Liu, B.; He, K.; Hu, M. | 2023 | *HumM2, CrAssphage, Human Adenovirus, Enterococcus* | Generally irrelevant, Review paper - primary research has been extracted |  |
| Meta-analysis of microbial source tracking for the identification of fecal contamination in aquatic environments based on data-mining. | Liu Z; Lin Y; Ge Y; Zhu Z; Yuan J; Yin Q; Liu B; He K; Hu M | 2023 | *Bacteroides HF183* | Review paper - primary research has been extracted |  |
| Metagenomic Evaluation of the Highly Abundant Human Gut Bacteriophage CrAssphage for Source Tracking of Human Fecal Pollution | Stachler, E.; Bibby, K. | 2014 | *CrAssphage* | No quantifiable sensitivity, specificity or accuracy |  |
| Metagenomics and the development of viral water quality tools | Bibby, K.; Crank, K.; Greaves, J.; Li, X.; Wu, Z.; Hamza, I.A.; Stachler, E. | 2019 | *CrAssphage* | Review paper - primary research has been extracted |  |
| Metagenomics for the study of viruses in urban sewage as a tool for public health surveillance | Fernandez-Cassi, X.; Timoneda, N.; Martínez-Puchol, S.; Rusiñol, M.; Rodriguez-Manzano, J.; Figuerola, N.; Bofill-Mas, S.; Abril, J.F.; Girones, R. | 2018 | *Human Adenovirus* | No quantifiable sensitivity, specificity or accuracy |  |
| Method for rapid and sensitive detection of Enterococcus sp. and Enterococcus faecalis/faecium cells in potable water samples | Maheux, A.F.; Bissonnette, L.; Boissinot, M.; Bernier, J.L.T.; Huppé, V.; Bérubé, E.; Boudreau, D.K.; Picard, F.J.; Huletsky, A.; Bergeron, M.G. | 2011 | *Enterococcus* | Generally irrelevant |  |
| Microbial Indicators of Fecal Pollution: Recent Progress and Challenges in Assessing Water Quality | Holcomb, D.A.; Stewart, J.R. | 2020 | *CrAssphage, Bacteroides HF183, Human Adenovirus, Enterococcus* | Review paper - primary research has been extracted, Generally irrelevant |  |
| Microbial source tracking for identification of fecal pollution | Seurinck, S.; Verstraete, W.; Siciliano, S.D. | 2005 | *Bacteroides HF183, Enterococcus* | Generally irrelevant, Review paper - primary research has been extracted |  |
| Microbial source tracking in highly vulnerable karst drinking water resources. | Diston D; Robbi R; Baumgartner A; Felleisen R | 2018 | *Bacteroides HF183* | Other - Please leave note | Regarding environemntal health |
| Microbial source tracking in impaired watersheds using PhyloChip and machine-learning classification | Dubinsky, E.A.; Butkus, S.R.; Andersen, G.L. | 2016 | *Faecalibacterium* | Generally irrelevant |  |
| Microbial source tracking in shellfish harvesting waters in the Gulf of Nicoya, Costa Rica | Symonds, E.M.; Young, S.; Verbyla, M.E.; McQuaig-Ulrich, S.M.; Ross, E.; Jiménez, J.A.; Harwood, V.J.; Breitbart, M. | 2017 | *Human Adenovirus* | Generally irrelevant |  |
| Microbial source tracking markers for detection of fecal contamination in environmental waters: Relationships between pathogens and human health outcomes | Harwood, V.J.; Staley, C.; Badgley, B.D.; Borges, K.; Korajkic, A. | 2014 | *Human Polyomavirus, Bacteroides thetaiotamicron, Bacteroides HF183, Bifidobacterium, Human Adenovirus, Enterococcus* | Review paper - primary research has been extracted |  |
| Microbial source tracking using molecular and cultivable methods in a tropical mixed-use drinking water source to support water safety plans | Sresung, M.; Paisantham, P.; Ruksakul, P.; Kongprajug, A.; Chyerochana, N.; Gallage, T.P.; Srathongneam, T.; Rattanakul, S.; Maneein, S.; Surasen, C.; Passananon, S.; Mongkolsuk, S.; Sirikanchana, K. | 2023 | *CrAssphage* | No quantifiable sensitivity, specificity or accuracy |  |
| Microbial source tracking using quantitative and digital PCR to identify sources of fecal contamination in stormwater, river water, and beach water in a Great Lakes area of concern | Staley, Z.R.; Boyd, R.J.; Shum, P.; Edge, T.A. | 2018 | *Faecalibacterium, Enterococcus* | Generally irrelevant |  |
| Microbial source tracking:  Characterization of human fecal pollution in environmental waters with HF183 quantitative real-time PCR | Shanks, O.C.; Korajkic, A. | 2019 | *Bacteroides HF183* | Review paper - primary research has been extracted |  |
| Microbial source tracking: State of the science | Simpson, J.M.; Santo Domingo, J.W.; Reasoner, D.J. | 2002 | *Enterococcus* | Generally irrelevant |  |
| Microbiological Water Quality of the Danube River: Status Quo and Future Perspectives | Kirschner, A.K.T.; Kavka, G.; Reischer, G.H.; Sommer, R.; Blaschke, A.P.; Stevenson, M.; Vierheilig, J.; Mach, R.L.; Farnleitner, A.H. | 2015 | *Enterococcus* | Generally irrelevant |  |
| Modeling fate and transport of fecally-derived microorganisms at the watershed scale: State of the science and future opportunities | Cho, K.H.; Pachepsky, Y.A.; Oliver, D.M.; Muirhead, R.W.; Park, Y.; Quilliam, R.S.; Shelton, D.R. | 2016 | *Enterococcus* | No quantifiable sensitivity, specificity or accuracy |  |
| Modeling fecal indicator bacteria concentrations in natural surface waters: A review | De Brauwere, A.; Ouattara, N.K.; Servais, P. | 2014 | *Enterococcus* | Review paper - primary research has been extracted |  |
| Molecular detection of viruses in water and sewage | La Rosa, G.; Muscillo, M. | 2013 | *Human Adenovirus* | Generally irrelevant |  |
| Molecular methods for pathogenic bacteria detection and recent advances in wastewater analysis | Zhang, S.; Li, X.; Wu, J.; Coin, L.; O’brien, J.; Hai, F.; Jiang, G. | 2021 | *Enterococcus* | Generally irrelevant |  |
| Molecular methods for the detection of waterborne pathogens | Gilbride, K. | 2020 | *Enterococcus* | Generally irrelevant |  |
| Molecular methods in biological systems | McMahon, K.D.; Gu, A.Z.; Nerenberg, R.; Sturm, B.M. | 2008 | *Human Adenovirus* | Review paper - primary research has been extracted |  |
| Molecular methods in biological systems | Angenent, L.T.; McMahon, K.D.; Gu, A.Z.; Nerenberg, R. | 2006 | *Human Adenovirus, Enterococcus, Bacteroides thetaiotamicron* | Generally irrelevant |  |
| Molecular methods in biological systems | Gu, A.Z.; Nerenberg, R.; Sturm, B.M.; Chul, P.; Goel, R. | 2010 | *Enterococcus* | Review paper - primary research has been extracted |  |
| Monitoring approaches for faecal indicator bacteria in water: Visioning a remote real-time sensor for e. coli and enterococci | Offenbaume, K.L.; Bertone, E.; Stewart, R.A. | 2020 | *Enterococcus* | No quantifiable sensitivity, specificity or accuracy |  |
| New methods for the concentration of viruses from urban sewage using quantitative PCR | Calgua, B.; Rodriguez-Manzano, J.; Hundesa, A.; Suñen, E.; Calvo, M.; Bofill-Mas, S.; Girones, R. | 2013 | *Human Polyomavirus* | No quantifiable sensitivity, specificity or accuracy |  |
| New Molecular Methods for Detection of Waterborne Pathogens | Cupples, A.M.; Xagoraraki, I.; Rose, J.B. | 2010 | *Human Polyomavirus, Human Adenovirus, Enterococcus* | Generally irrelevant, No quantifiable sensitivity, specificity or accuracy, Review paper - primary research has been extracted |  |
| Pathogens, faecal indicators and human-specific microbial source-tracking markers in sewage | García-Aljaro, C.; Blanch, A.R.; Campos, C.; Jofre, J.; Lucena, F. | 2019 | *CrAssphage, Bacteroides thetaiotamicron, Human Adenovirus, Enterococcus* | Generally irrelevant, Review paper - primary research has been extracted |  |
| Performance evaluation of Bacteroidales genetic markers for human and animal microbial source tracking in tropical agricultural watersheds | Somnark, P.; Chyerochana, N.; Mongkolsuk, S.; Sirikanchana, K. | 2018 | *Bacteroides thetaiotamicron* | Generally irrelevant |  |
| Performance evaluation of Bacteroidales genetic markers for human and animal microbial source tracking in tropical agricultural watersheds. | Somnark P; Chyerochana N; Mongkolsuk S; Sirikanchana K | 2018 | *Bacteroides HF183* | Other - Please leave note | Uses the same samples/results from another article |
| Performance of bacterial and mitochondrial qPCR source tracking methods: A European multi-center study | Monteiro, S.; Machado-Moreira, B.; Linke, R.; Blanch, A.R.; Ballesté, E.; Méndez, J.; Maunula, L.; Oristo, S.; Stange, C.; Tiehm, A.; Farnleitner, A.H.; Santos, R.; García-Aljaro, C. | 2023 | *Bacteroides thetaiotamicron, Human Adenovirus, Enterococcus, Bifidobacterium* | Generally irrelevant |  |
| Performance of forty-one microbial source tracking methods: A twenty-seven lab evaluation study | Boehm, A.B.; Van De Werfhorst, L.C.; Griffith, J.F.; Holden, P.A.; Jay, J.A.; Shanks, O.C.; Wang, D.; Weisberg, S.B. | 2013 | *Human Adenovirus* | Generally irrelevant |  |
| Performance of forty-one microbial source tracking methods: a twenty-seven lab evaluation study. | Boehm AB; Van De Werfhorst LC; Griffith JF; Holden PA; Jay JA; Shanks OC; Wang D; Weisberg SB | 2013 | *Enterococcus* | No quantifiable sensitivity, specificity or accuracy |  |
| Performance of viruses and bacteriophages for fecal source determination in a multi-laboratory, comparative study. | Harwood VJ; Boehm AB; Sassoubre LM; Vijayavel K; Stewart JR; Fong TT; Caprais MP; Converse RR; Diston D; Ebdon J; Fuhrman JA; Gourmelon M; Gentry-Shields J; Griffith JF; Kashian DR; Noble RT; Taylor H; Wicki M | 2013 | *Enterococcus, Human Polyomavirus, Human Adenovirus* | No quantifiable sensitivity, specificity or accuracy |  |
| Performance, design, and analysis in microbial source tracking studies | Stoeckel, D.M.; Harwood, V.J. | 2007 | *Bacteroides thetaiotamicron, Bacteroides HF183, Bifidobacterium, Human Adenovirus, Enterococcus* | Review paper - primary research has been extracted |  |
| Phenotypic library-based microbial source tracking methods: efficacy in the California collaborative study. | Harwood VJ; Wiggins B; Hagedorn C; Ellender RD; Gooch J; Kern J; Samadpour M; Chapman AC; Robinson BJ; Thompson BC | 2003 | *Enterococcus* | No quantifiable sensitivity, specificity or accuracy |  |
| Phenotypic population characteristics of the enterococci in wastewater and animal faeces: implications for the new European directive on the quality of bathing waters. | Wallis JL; Taylor HD | 2003 | *Enterococcus* | Full text unavailable |  |
| Phylogenetic analysis of Bacteroidales 16S rRNA gene sequences from human and animal effluents and assessment of ruminant faecal pollution by real-time PCR | Mieszkin, S.; Yala, J.-F.; Joubrel, R.; Gourmelon, M. | 2010 | *Bacteroides HF183* | Generally irrelevant |  |
| Pollution characteristics and source track of fecal microorganism in the rivers across Chongqing city | Zhang, L.-J.; Wang, G.-G.; Zhang, L.-L.; Ruan, X.-Y.; Zhou, S.-H.; Zhang, D.-J.; Zhao, L.; Wang, P.-F. | 2019 | *Bacteroides HF183* | Full text unavailable |  |
| Polyomaviruses | DeCaprio, J.A.; Imperiale, M.J.; Major, E.O. | 2013 | *Human Polyomavirus* | Generally irrelevant |  |
| Potential of Enterococcus faecalis as a human fecal indicator for microbial source tracking. | Wheeler AL; Hartel PG; Godfrey DG; Hill JL; Segars WI | 2002 | *Enterococcus* | No quantifiable sensitivity, specificity or accuracy |  |
| Quantification of host-specific Bacteroides-Prevotella 16S rRNA genetic markers for assessment of fecal pollution in freshwater | Okabe, S.; Okayama, N.; Savichtcheva, O.; Ito, T. | 2007 | *Bacteroides HF183* | Generally irrelevant |  |
| Quantitative CrAssphage PCR Assays for Human Fecal Pollution Measurement | Stachler, E.; Kelty, C.; Sivaganesan, M.; Li, X.; Bibby, K.; Shanks, O.C. | 2017 | *HumM2* | Review paper - primary research has been extracted |  |
| Quantitative identification of fecal water pollution sources by TaqMan real-time PCR assays using Bacteroidales 16S rRNA genetic markers | Lee, D.-Y.; Weir, S.C.; Lee, H.; Trevors, J.T. | 2010 | *Bacteroides HF183* | Generally irrelevant |  |
| Quantitative PCR for genetic markers of human fecal pollution | Shanks, O.C.; Kelty, C.A.; Sivaganesan, M.; Varma, M.; Haugland, R.A. | 2009 | *Bacteroides thetaiotamicron, Bacteroides HF183, Enterococcus* | Generally irrelevant |  |
| Quo vadis source tracking? Towards a strategic framework for environmental monitoring of fecal pollution | Santo Domingo, J.W.; Bambic, D.G.; Edge, T.A.; Wuertz, S. | 2007 | *Methanobrevibacter smithii, Human Adenovirus, Enterococcus* | Generally irrelevant, Review paper - primary research has been extracted |  |
| Rapid and in-situ detection of fecal indicator bacteria in water using simple DNA extraction and portable loop-mediated isothermal amplification (LAMP)PCR methods | Lee, S.; Khoo, V.S.L.; Medriano, C.A.D.; Lee, T.; Park, S.-Y.; Bae, S. | 2019 | *Enterococcus* | Generally irrelevant |  |
| Rapid QPCR-based assay for fecal Bacteroides spp. as a tool for assessing fecal contamination in recreational waters | Converse, R.R.; Blackwood, A.D.; Kirs, M.; Griffith, J.F.; Noble, R.T. | 2009 | *Bacteroides thetaiotamicron, Bacteroides HF183, Enterococcus* | Generally irrelevant |  |
| Rapid tests for detection and quantitation of Enterococcus contamination in recreational waters. | Morgan R; Morris C; Livzey K; Hogan J; Buttigieg N; Pollner R; Kacian D; Weeks I | 2007 | *Enterococcus* | Generally irrelevant |  |
| Recommendations following a multi-laboratory comparison of microbial source tracking methods. | Stewart JR; Boehm AB; Dubinsky EA; Fong TT; Goodwin KD; Griffith JF; Noble RT; Shanks OC; Vijayavel K; Weisberg SB | 2013 | *Enterococcus* | Generally irrelevant |  |
| Reduction of human fecal markers and enteric viruses in Sydney estuarine waters receiving wet weather overflows | Ahmed, W.; Payyappat, S.; Cassidy, M.; Harrison, N.; Besley, C. | 2023 | *Enterococcus* | No quantifiable sensitivity, specificity or accuracy |  |
| Relationship between Rainfall, fecal pollution, antimicrobial resistance, and microbial diversity in an urbanized subtropical bay | Powers, N.C.; Wallgren, H.R.; Marbach, S.; Turner, J.W. | 2020 | *Enterococcus* | No quantifiable sensitivity, specificity or accuracy |  |
| Relationships between microbial indicators and pathogens in recreational water settings | Korajkic, A.; McMinn, B.R.; Harwood, V.J. | 2018 | *Enterococcus* | No quantifiable sensitivity, specificity or accuracy |  |
| Research progress of microbial source tracking based on Bacteroidales 16S rRNA gene | Liang, H.-X.; Yu, Z.-S.; Liu, R.-Y.; Zhang, H.-X.; Wu, G. | 2018 | *Bacteroides HF183* | Full text unavailable |  |
| Searching for a Reliable Viral Indicator of Faecal Pollution in Aquatic Environments | Andrianjakarivony, F.H.; Bettarel, Y.; Desnues, C. | 2023 | *Human Polyomavirus, Human Adenovirus* | Review paper - primary research has been extracted |  |
| Selection of a diagnostic tool for microbial water quality monitoring and management of faecal contamination of water sources in rural communities | Murei, A.; Kamika, I.; Momba, M.N.B. | 2024 | *Enterococcus* | No quantifiable sensitivity, specificity or accuracy |  |
| Selection of microbial and chemical markers for microbail source tracking. Application to river waters impacted by point and non-point source pollution in France | Pourcher, A.M.; Jardé, E.; Caprais, M.P.; Wéry, N.; Jadas-Hécart, A.; Communal, P.Y.; Jaffrezic, A.; Marti, R.; Mieszkin, S.; Derrien, M.; Solecki, O.; Jeanneau, L.; Gourmelon, M. | 2012 | *Bacteroides HF183* | Full text unavailable |  |
| Sequence-enabled community-based microbial source tracking in surface waters using machine learning classification: A review | Mathai, P.P.; Staley, C.; Sadowsky, M.J. | 2020 | *Enterococcus* | Review paper - primary research has been extracted |  |
| Source Tracking in Australia and New Zealand: Case Studies | Ahmed, W; Kirs, M; Gilpin, B | 2011 | *Enterococcus* | Generally irrelevant |  |
| Source tracking of Enterococcus moraviensis and E. haemoperoxidus. | Taučer-Kapteijn M; Hoogenboezem W; Hoogenboezem R; de Haas S; Medema G | 2017 | *Enterococcus* | No quantifiable sensitivity, specificity or accuracy |  |
| Sourcing faecal pollution: A combination of library-dependent and library-independent methods to identify human faecal pollution in non-sewered catchments | Ahmed, W.; Stewart, J.; Gardner, T.; Powell, D.; Brooks, P.; Sullivan, D.; Tindale, N. | 2007 | *Enterococcus* | No quantifiable sensitivity, specificity or accuracy |  |
| State of the art molecular markers for fecal pollution source tracking in water | Roslev, P.; Bukh, A.S. | 2011 | *Human Polyomavirus, Faecalibacterium, Bacteroides thetaiotamicron, Bacteroides HF183, Enterococcus* | Review paper - primary research has been extracted, Generally irrelevant |  |
| Surface Water Quality in Punjab, India: Tracking Human and Farm Animal-Specific Adenoviral Contamination and Correlation with Microbiological and Physiochemical Parameters | Zehra, A.; Kaur, S.; Singh, R.; Gill, J.P.S. | 2020 | *Human Adenovirus* | Generally irrelevant |  |
| Targeted Sampling Protocol as Prelude to Bacterial Source Tracking with Enterococcus faecalis | Kuntz, R.L.; Hartel, P.G.; Godfrey, D.G.; McDonald, J.L.; Gates, K.W.; Segars, W.I. | 2003 | *Enterococcus* | No quantifiable sensitivity, specificity or accuracy |  |
| Toolbox approaches using molecular markers and 16S rRNA gene amplicon data sets for identification of fecal pollution in surface water | Ahmed, W.; Staley, C.; Sadowsky, M.J.; Gyawali, P.; Sidhu, J.; Palmer, A.; Beale, D.J.; Toze, S. | 2015 | *Bacteroides HF183* | No quantifiable sensitivity, specificity or accuracy |  |
| Tools for interpretation of wastewater SARS-CoV-2 temporal and spatial trends demonstrated with data collected in the San Francisco Bay Area | Greenwald, H.D.; Kennedy, L.C.; Hinkle, A.; Whitney, O.N.; Fan, V.B.; Crits-Christoph, A.; Harris-Lovett, S.; Flamholz, A.I.; Al-Shayeb, B.; Liao, L.D.; Beyers, M.; Brown, D.; Chakrabarti, A.R.; Dow, J.; Frost, D.; Koekemoer, M.; Lynch, C.; Sarkar, P.; White, E.; Kantor, R.; Nelson, K.L. | 2021 | *CrAssphage* | No quantifiable sensitivity, specificity or accuracy |  |
| Tracking the primary sources of fecal pollution in a tropical watershed in a one-year study. | Toledo-Hernandez C; Ryu H; Gonzalez-Nieves J; Huertas E; Toranzos GA; Santo Domingo JW | 2013 | *Enterococcus* | No quantifiable sensitivity, specificity or accuracy |  |
| Traditional and molecular analyses for fecal indicator bacteria in non-point source subtropical recreational marine waters | Sinigalliano, C.D.; Fleisher, J.M.; Gidley, M.L.; Solo-Gabriele, H.M.; Shibata, T.; Plano, L.R.W.; Elmir, S.M.; Wanless, D.; Bartkowiak, J.; Boiteau, R.; Withum, K.; Abdelzaher, A.M.; He, G.; Ortega, C.; Zhu, X.; Wright, M.E.; Kish, J.; Hollenbeck, J.; Scott, T.; Backer, L.C.; Fleming, L.E. | 2010 | *Enterococcus* | Generally irrelevant |  |
| Ultrafiltration and microarray for detection of microbial source tracking marker and pathogen genes in riverine and marine systems | Li, X.; Harwood, V.J.; Nayak, B.; Weidhaas, J.L. | 2016 | *Human Adenovirus* | No quantifiable sensitivity, specificity or accuracy |  |
| Ultrafiltration and Microarray for Detection of Microbial Source Tracking Marker and Pathogen Genes in Riverine and Marine Systems. | Li X; Harwood VJ; Nayak B; Weidhaas JL | 2016 | *Human Polyomavirus* | Generally irrelevant |  |
| Use of Escherichia coli genes associated with human sewage to track fecal contamination source in subtropical waters. | Senkbeil JK; Ahmed W; Conrad J; Harwood VJ | 2019 | *Bacteroides HF183* | No quantifiable sensitivity, specificity or accuracy |  |
| Using an intervening sequence of Faecalibacterium 16S rDNA to identify poultry feces | Shen, Z.; Duan, C.; Zhang, C.; Carson, A.; Xu, D.; Zheng, G. | 2013 | *Faecalibacterium* | No quantifiable sensitivity, specificity or accuracy |  |
| Using DNA microarrays to identify library-independent markers for bacterial source tracking. | Soule M; Kuhn E; Loge F; Gay J; Call DR | 2006 | *Enterococcus* | No quantifiable sensitivity, specificity or accuracy |  |
| Using DNA suspension arrays to identify library-independent markers for bacterial source tracking. | Call DR; Satterwhite DM; Soule M | 2007 | *Enterococcus* | Other - Please leave note | Regarding environemntal health |
| Validation and application of quantitative PCR assays using host-specific Bacteroidales genetic markers for swine fecal pollution tracking | Fan, L.; Shuai, J.; Zeng, R.; Mo, H.; Wang, S.; Zhang, X.; He, Y. | 2017 | *Bacteroides HF183* | Generally irrelevant |  |
| Validation and field testing of library-independent microbial source tracking methods in the Gulf of Mexico | Harwood, V.J.; Brownell, M.; Wang, S.; Lepo, J.; Ellender, R.D.; Ajidahun, A.; Hellein, K.N.; Kennedy, E.; Ye, X.; Flood, C. | 2009 | *Bacteroides HF183* | Generally irrelevant |  |
| Viral and bacterial fecal indicators in untreated wastewater across the contiguous united states exhibit geospatial trends | Korajkic, A.; McMinn, B.; Herrmann, M.P.; Sivaganesan, M.; Kelty, C.A.; Clinton, P.; Nash, M.S.; Shanks, O.C. | 2020 | *Human Polyomavirus, Enterococcus, Bacteroides HF183* | No quantifiable sensitivity, specificity or accuracy |  |
| Viral, bacterial, and protozoan pathogens and fecal markers in wells supplying groundwater to public water systems in Minnesota, USA | Stokdyk, J.P.; Firnstahl, A.D.; Walsh, J.F.; Spencer, S.K.; de Lambert, J.R.; Anderson, A.C.; Rezania, L.-I.W.; Kieke, B.A.; Borchardt, M.A. | 2020 | *HumM2, Bacteroides HF183* | Other - Please leave note | Regarding environmental health |
| Wastewater Microbiology: Fourth Edition | Bitton, G. | 2010 | *Bacteroides HF183, Human Adenovirus* | Generally irrelevant |  |
| Water quality indicators: Bacteria, coliphages, enteric viruses | Lin, J.; Ganesh, A. | 2013 | *Human Polyomavirus, Human Adenovirus* | Review paper - primary research has been extracted |  |
| Waterborne pathogens: Detection methods and challenges | Ramírez-Castillo, F.Y.; Loera-Muro, A.; Jacques, M.; Garneau, P.; Avelar-González, F.J.; Harel, J.; Guerrero-Barrera, A.L. | 2015 | *Human Adenovirus* | Review paper - primary research has been extracted |  |
| Evaluation of the host-specificity and prevalence of enterococci surface protein (esp) marker in sewage and its application for sourcing human fecal pollution | Ahmed, W.; Stewart, J.; Powell, D.; Gardner, T. | 2008 | *Enterococcus* | Other - Please leave note | Uses the same samples/results from another article |
| Global Distribution of Human-Associated Fecal Genetic Markers in Reference Samples from Six Continents | Mayer, R.E.; Reischer, G.H.; Ixenmaier, S.K.; Derx, J.; Blaschke, A.P.; Ebdon, J.E.; Linke, R.; Egle, L.; Ahmed, W.; Blanch, A.R.; Byamukama, D.; Savill, M.; Mushi, D.; Cristóbal, H.A.; Edge, T.A.; Schade, M.A.; Aslan, A.; Brooks, Y.M.; Sommer, R.; Masago, Y.; Sato, M.I.; Taylor, H.D.; Rose, J.B.; Wuertz, S.; Shanks, O.C.; Piringer, H.; Mach, R.L.; Savio, D.; Zessner, M.; Farnleitner, A.H. | 2018 | *Bacteroides HF183* | Other - Please leave note | Geographic location is not distinguished between samples |
| Quantitative PCR for genetic markers of human fecal pollution | Shanks OC; Kelty CA; Sivaganesan M; Varma M; Haugland RA | 2009 | *HumM3, HumM2* | Geographic location is not distinguished between samples |  |
| Development of Faecalibacterium 16S rRNA gene marker for identification of human faeces. | Zheng G; Yampara-Iquise H; Jones JE; Andrew Carson C | 2009 | *Faecalibacterium* | Geographic location is not distinguished between samples |  |
| Quantification of human polyomaviruses JC Virus and BK Virus by TaqMan quantitative PCR and comparison to other water quality indicators in water and fecal samples. | McQuaig SM; Scott TM; Lukasik JO; Paul JH; Harwood VJ | 2009 | *Methanobrevibacter smithii* | Geographic location is not distinguished between samples |  |
| Bacteriophages Are Good Estimators of Human Viruses Present in Water. | Ballesté E; Blanch AR; Mendez J; Sala-Comorera L; Maunula L; Monteiro S; Farnleitner AH; Tiehm A; Jofre J; García-Aljaro C | 2021 | *Bacteroides thetaiotamicron, CrAssphage, Human Adenovirus* | Geographic location is not distinguished between samples |  |
| Evaluation of genetic markers from the 16S rRNA gene V2 region for use in quantitative detection of selected Bacteroidales species and human fecal waste by qPCR | Haugland, R.A.; Varma, M.; Sivaganesan, M.; Kelty, C.; Peed, L.; Shanks, O.C. | 2010 | *Bacteroides thetaiotamicron* | Geographic location is not distinguished between samples |  |
| Method for isolation of Bacteroides bacteriophage host strains suitable for tracking sources of fecal pollution in water. | Payan A; Ebdon J; Taylor H; Gantzer C; Ottoson J; Papageorgiou GT; Blanch AR; Lucena F; Jofre J; Muniesa M | 2005 | *Bacteroides thetaiotamicron* | Geographic location is not distinguished between samples |  |
| Performance of PCR-based assays targeting Bacteroidales genetic markers of human fecal pollution in sewage and fecal samples | Shanks, O.C.; White, K.; Kelty, C.A.; Sivaganesan, M.; Blannon, J.; Meckes, M.; Varma, M.; Haugland, R.A. | 2010 | *Bacteroides thetaiotamicron* | Geographic location is not distinguished between samples |  |
| Novel Bacteroides host strains for detection of human- and animal-specific bacteriophages in water | Wicki, M.; Auckenthaler, A.; Felleisen, R.; Tanner, M.; Baumgartner, A. | 2011 | *Bacteroides thetaiotamicron* | Geographic location is not distinguished between samples |  |
| Novel crAssphage marker genes ascertain sewage pollution in a recreational lake receiving urban stormwater runoff. | Ahmed W; Payyappat S; Cassidy M; Besley C; Power K | 2018 | CrAssphage | Geographic location is not distinguished between samples |  |
| Integrating Metagenomic and Bayesian Analyses to Evaluate the Performance and Confidence of CrAssphage as an Indicator for Tracking Human Sewage Contamination in China. | Chen H; Liu C; Li Y; Teng Y | 2021 | CrAssphage | Geographic location is not distinguished between samples |  |
| Quantitative CrAssphage PCR Assays for Human Fecal Pollution  Measurement | Stachler, E.; Kelty, C.; Sivaganesan, M.; Li, X.; Bibby, K.; Shanks, O.C. | 2017 | CrAssphage, Bacteroides HF183 | Geographic location is not distinguished between samples |  |
| Performance of host-associated genetic markers for microbial source tracking in China | Zhang, Y.; Wu, R.; Lin, K.; Wang, Y.; Lu, J. | 2020 | CrAssphage, Bacteroides HF183 | Geographic location is not distinguished between samples |  |
| Host Specificity and Sensitivity of Established and Novel Sewage-Associated Marker Genes in Human and Nonhuman Fecal Samples. | Ahmed W; Gyawali P; Feng S; McLellan SL | 2019 | Human Polyomavirus, CrAssphage, Human Adenovirus, *Bacteroides HF183* | Geographic location is not distinguished between samples |  |
| Distributions of fecal markers in wastewater from different climatic zones for human fecal pollution tracking in Australian surface waters | Ahmed, W.; Sidhu, J.P.S.; Smith, K.; Beale, D.J.; Gyawali, P.; Tozea, S. | 2016 | Human Adenovirus, *Bacteroides HF183* | Geographic location is not distinguished between samples |  |
| Detection and quantitation of infectious human adenoviruses and JC polyomaviruses in water by immunofluorescence assay | Calgua, B.; Barardi, C.R.M.; Bofill-Mas, S.; Rodriguez-Manzano, J.; Girones, R. | 2011 | Human Adenovirus, *Enterococcus* | Geographic location is not distinguished between samples |  |
| Torque teno virus occurrence and relationship to bacterial and viral indicators in feces, wastewaters, and waters in the United States | Plummer, J.D.; Long, S.C.; Liu, Z.; Charest, A.A. | 2014 | Human Adenovirus | Geographic location is not distinguished between samples |  |
| Ecological and Technical Mechanisms for Cross-Reaction of Human Fecal Indicators with Animal Hosts. | Feng S; Ahmed W; McLellan SL | 2020 | *Bacteroides HF183* | Geographic location is not distinguished between samples |  |
| Host-Associated Bacteroides 16S rDNA-Based Markers for Source Tracking of Fecal Pollution in Laguna Lake, Philippines. | Malajacan GT; Nacario MAG; Obusan MCM; Rivera WL | 2023 | *Bacteroides HF183* | Geographic location is not distinguished between samples |  |
| The Use of Ribosomal RNA as a Microbial Source Tracking Target Highlights the Assay Host-Specificity Requirement in Water Quality Assessments. | Rytkönen A; Tiwari A; Hokajärvi AM; Uusheimo S; Vepsäläinen A; Tulonen T; Pitkänen T | 2021 | *Bacteroides HF183* | Geographic location is not distinguished between samples |  |
| Quantification of Microbial Source Tracking and Pathogenic Bacterial Markers in Water and Sediments of Tiaoxi River (Taihu Watershed). | Vadde KK; McCarthy AJ; Rong R; Sekar R | 2019 | *Bacteroides HF183* | Geographic location is not distinguished between samples |  |
| A novel droplet digital PCR human mtDNA assay for fecal source tracking. | Zhu K; Suttner B; Pickering A; Konstantinidis KT; Brown J | 2020 | *Bacteroides HF183* | Geographic location is not distinguished between samples |  |

**Table S3. 2:** List of all studies included within the systematic review the markers they explored and the geographic context.

| **Title** | **Author** | **Year** | **Country** | **Continent** | **Development** | **Climate Zone** | **Climate** | **Marker** |
| --- | --- | --- | --- | --- | --- | --- | --- | --- |
| A duplex PCR assay for the simultaneous quantification of Bacteroides HF183 and crAssphage CPQ_056 marker genes in untreated sewage and stormwater. | Ahmed W; Payyappat S; Cassidy M; Besley C | 2019 | Australia | Oceania | HIC | Arid | BSh | CrAssphage, *Bacteroides HF183* |
| Applicability of crAssphage, pepper mild mottle virus, and tobacco mosaic virus as indicators of reduction of enteric viruses during wastewater treatment | Tandukar, Sarmila; Sherchan, Samendra P; Haramoto, Eiji | 2010 | United States | North America | HIC | Temperate | Cfa | CrAssphage |
| Application of crAssphage, F-RNA phage and pepper mild mottle virus as indicators of human faecal and norovirus contamination in shellfish. | Gyawali P; Devane M; Scholes P; Hewitt J | 2021 | New Zealand | Oceania | HIC | Temperate | Cfb | CrAssphage |
| Application of Faecalibacterium 16S rDNA genetic marker for accurate identification of duck faeces. | Sun D; Duan C; Shang Y; Ma Y; Tan L; Zhai J; Gao X; Guo J; Wang G | 2016 | China | Asia | LMIC | Temperate | Cfa | *Faecalibacterium* |
| Assessing the faecal source sensitivity and specificity of ruminant and human genetic microbial source tracking markers in the central Ethiopian highlands. | Linke RB; Kebede G; Mushi D; Lakew A; Hayes DS; Graf W; Farnleitner AH | 2021 | Ethiopia | Africa | *LMIC* | Arid | BSh | *Bacteroides HF183* |
| Assessment of Human Health Risks in Tropical Environmental Waters with Microbial Source Tracking Markers. | Goh SG; Liang L; Gin KYH | 2021 | Singapore | Asia | HIC | Tropical | Af | *Methanobrevibacter smithii, Bacteroides thetaiotamicron* |
| Bacteroidales markers for microbial source tracking in Southeast Asia | Nshimyimana, JP; Cruz, MC; Thompson, RJ; Wuertz, S | 2017 | Singapore | Asia | HIC | Tropical | Af | *Bacteroides HF183, Bacteroides thetaiotamicron* |
| Bacteroides spp. and traditional fecal indicator bacteria in water quality assessment – An integrated approach for hydric resources management in urban centers | Teixeira, P.; Dias, D.; Costa, S.; Brown, B.; Silva,  S.; Valério, E. | 2020 | Portugal | Europe | HIC | Temperate | Csa | *Bacteroides HF183* |
| Comparative fate of CrAssphage with culturable and molecular fecal pollution indicators during activated sludge wastewater treatment | Wu, Zhenyu; Greaves, Justin; Arp, Lillian; Stone, Daniel; Bibby, Kyle | 2020 | United States | North America | HIC | Cold | Dfa | CrAssphage |
| Comparative microbial source tracking methods for identification of fecal contamination sources at Sunnyside Beach in the Toronto region area of concern | Staley, Z.R.; Edge, T.A. | 2016 | Canada | North America | HIC | Cold | Dfc | *Bacteroides HF183* |
| Comparison of molecular markers to detect fresh sewage in environmental waters | Ahmed, W.; Goonetilleke, A.; Powell, D.; Chauhan, K.; Gardner, T. | 2009 | Australia | Oceania | HIC | Arid | BSh | *Human Polyomavirus, Human Adenovirus, Enterococcus, Bacteroides HF183* |
| Comparison of the host specificities of two Bacteroidales quantitative PCR assays used for tracking human fecal contamination | Van De Werfhorst, L.C.; Sercu, B.; Holden, P.A. | 2011 | United States | North America | HIC | Temperate | Csa | *Bacteroides HF183* |
| Comparison of the performance of different microbial source tracking markers among European and North African regions | Yahya, M.; Blanch, A.R.; Meijer, W.G.; Antoniou, K.; Hmaied, F.; Ballesté, E. | 2017 | Tunisia, Spain, Cyprus, Ireland | Africa, Europe | LMIC, HIC | Arid, Temperate | BSh, Csa, Cfb | *Bifidobacterium, Bacteroides HF183* |
| *Consistency in the host specificity and host sensitivity of the Bacteroides HF183 marker for sewage pollution tracking* | Ahmed, W.; Masters, N.; Toze, S. | 2012 | Australia | Oceania | HIC | Arid | BSh | *Bacteroides HF183* |
| crAssphage as a human molecular marker to evaluate temporal and spatial variability in faecal contamination of urban marine bathing waters. | Sala-Comorera L; Reynolds LJ; Martin NA; Pascual-Benito M; Stephens JH; Nolan TM; Gitto A; O'Hare GMP; O'Sullivan JJ; García-Aljaro C; Meijer WG | 2021 | Ireland | Europe | HIC | Temperate | Cfb | CrAssphage |
| CrAssphage as a Potential Human Sewage Marker for Microbial Source Tracking in Southeast Asia | Kongprajug, A.; Mongkolsuk, S.; Sirikanchana, K. | 2019 | Thailand | Asia | LMIC | Tropical | Aw | CrAssphage, *Bacteroides HF183* |
| Critical evaluation of CrAssphage as a molecular marker for human-derived wastewater contamination in the aquatic environment | Farkas, Kata; Adriaenssens, Evelien M; Walker, David I; McDonald, James E; Malham, Shelagh K; Jones, Davey L | 2019 | United Kingdom | Europe | HIC | Temperate | Cfb | CrAssphage |
| Design and evaluation of Bacteroides DNA probes for the specific detection of human fecal pollution | Kreader, Carol A | 1995 | United States | North America | HIC | Cold | Dfa | *Bacteroides thetaiotamicron* |
| *Detection and quantification of the human-specific HF183 Bacteroides 16S rRNA genetic marker with real-time PCR for assessment of human faecal pollution in freshwater* | Seurinck, S.; Defoirdt, T.; Verstraete, W.; Siciliano, S.D. | 2005 | Belgium | Europe | HIC | Temperate | Cfb | *Bacteroides HF183* |
| Detection and source identification of faecal pollution in non-sewered catchment by means of host-specific molecular markers | Ahmed, W.; Powell, D.; Goonetilleke, A.; Gardner, T. | 2008 | Australia | Oceania | HIC | Arid | Bsh | *Enterococcus* |
| Detection of BK, JC, WU, or KI polyomaviruses in faecal, urine, blood, cerebrospinal fluid and respiratory samples | Bialasiewicz, Seweryn; Whiley, David M; Lambert, Stephen B; Nissen, Michael D; Sloots, Theo P | 2009 | Australia | Oceania | HIC | Arid | Bsh | *Human Polyomavirus* |
| Detection of genetic markers of fecal indicator bacteria in Lake Michigan and determination of their relationship to Escherichia coli densities using standard microbiological methods | Bower, Patricia A; Scopel, Caitlin O; Jensen, Erika T; Depas, Morgan M; McLellan, Sandra L | 2005 | United States | North America | HIC | Cold | Dfb | *Bacteroides HF183* |
| Detection of human-derived fecal pollution in environmental waters by use of a PCR-based human polyomavirus assay. | McQuaig SM; Scott TM; Harwood VJ; Farrah SR; Lukasik JO | 2006 | United States | North America | HIC | Temperate | Cfa | *Human Polyomavirus* |
| Detection of the nifH gene of Methanobrevibacter smithii: A potential tool to identify sewage pollution in recreational waters | Ufnar, J.A.; Wang, S.Y.; Christiansen, J.M.; Yampara-Iquise, H.; Carson, C.A.; Ellender, R.D. | 2006 | United States | North America | HIC | Temperate | Cfa | *Methanobrevibacter smithii* |
| Determination of crAssphage in water samples and applicability for tracking human faecal pollution | García‐Aljaro, Cristina; Ballesté, Elisenda; Muniesa, Maite; Jofre, Juan | 2017 | Spain | Europe | HIC | Temperate | Cfb | CrAssphage |
| Developing a novel Bifidobacterium phage quantitative polymerase chain reaction-based assay for tracking untreated wastewater. | Li X; Ahmed W; Wu Z; Xia Y | 2022 | China, Australia | Asia, Oceania | LMIC, HIC | Temperate | Cwa | *Bifidobacterium, Bacteroides HF183* |
| Development of a Luminex assay for the simultaneous detection of human enteric viruses in sewage and river water | Hamza, I.A.; Jurzik, L.; Wilhelm, M. | 2014 | Germany | Europe | HIC | Temperate | Cfb | *Human Polyomavirus, Human Adenovirus* |
| Development of a quantitative PCR assay for the quantitation of bovine polyomavirus as a microbial source-tracking tool | Hundesa, Ayalkibet; Bofill-Mas, Silvia; de Motes, Carlos Maluquer; Rodriguez-Manzano, Jesus; Bach, Alex; Casas, Maribel; Girones, Rosina | 2010 | Spain | Europe | HIC | Temperate | Cfb | *Human Polyomavirus* |
| Development of microbial and chemical MST tools to identify the origin of the faecal pollution in bathing and shellfish harvesting waters in France. | Gourmelon M; Caprais MP; Mieszkin S; Marti R; Wéry N; Jardé E; Derrien M; Jadas-Hécart A; Communal PY; Jaffrezic A; Pourcher AM | 2010 | France | Europe | HIC | Temperate | Cfb | *Bifidobacterium, Bacteroides HF183* |
| Development of new host-specific Bacteroides qPCRs for the identification of fecal contamination sources in water. | Gómez-Doñate M; Casanovas-Massana A; Muniesa M; Blanch AR | 2016 | Spain | Europe | HIC | Temperate | Cfb | *Bacteroides HF183* |
| Distribution and diversity of the enterococcal surface protein (esp) gene in animal hosts and the Pacific coast environment. | Layton BA; Walters SP; Boehm AB | 2009 | United States | North America | HIC | Temperate | Csa | *Enterococcus* |
| Documenting the epidemiologic patterns of polyomaviruses in human populations by studying their presence in urban sewage | Bofill-Mas, Sílvia; Pina, Sonia; Girones, Rosina | 2000 | Spain, Sweden, France, South Africa | Europe, Africa | HIC, LMIC | Temperate, Cold | Cfb, Cwb, Dfc | *Human Polyomavirus* |
| Dynamics of crAssphage as a human source tracking marker in potentially faecally polluted environments | Ballesté, E; Pascual-Benito, Miriam; Martín-Díaz, Julia; Blanch, AR; Lucena, F; Muniesa, M; Jofre, J; García-Aljaro, Cristina | 2019 | Spain | Europe | HIC | Temperate | Cfb | CrAssphage |
| Effect of Quantitative Polymerase Chain Reaction Data Analysis Using Sample Amplification Efficiency on Microbial Source Tracking Assay Performance and Source Attribution. | Kongprajug A; Chyerochana N; Mongkolsuk S; Sirikanchana K | 2020 | Thailand | Asia | LMIC | Tropical | Aw | *Bacteroides HF183* |
| Effectiveness of two wastewater disinfection strategies for the removal of fecal indicator bacteria, bacteriophage, and enteric viral pathogens concentrated using dead-end hollow fiber ultrafiltration (D-HFUF) | Korajkic, A.; Kelleher, J.; Shanks, O.C.; Herrmann, M.P.; McMinn, B.R. | 2022 | United States | North America | HIC | Cold | Dfa | *Human Polyomavirus* |
| Estimation of pig fecal contamination in a river catchment by real-time PCR using two pig-specific Bacteroidales 16S rRNA genetic markers. | Mieszkin S; Furet JP; Corthier G; Gourmelon M | 2009 | France | Europe | HIC | Temperate | Cfb | *Bacteroides HF183* |
| Evaluating sewage-associated JCV and BKV polyomaviruses for sourcing human fecal pollution in a coastal river in Southeast Queensland, Australia | Ahmed, W.; Wan, C.; Goonetilleke, A.; Gardner, T. | 2010 | Australia | Oceania | HIC | Arid | BSh | *Human Polyomavirus* |
| Evaluation of Bacteroides markers for the detection of human faecal pollution | Ahmed, W.; Stewart, J.; Powell, D.; Gardner, T. | 2008 | Australia | Oceania | HIC | Arid | BSh | *Bacteroides HF183* |
| Evaluation of crAssphage as a human-specific microbial source-tracking marker in the Republic of Korea. | Nam SJ; Hu WS; Koo OK | 2022 | South Korea | Asia | HIC | Cold | Dwa | CrAssphage |
| Evaluation of five microbial and four mitochondrial DNA markers for tracking human and pig fecal pollution in freshwater. | He X; Liu P; Zheng G; Chen H; Shi W; Cui Y; Ren H; Zhang XX | 2016 | China | Asia | LMIC | Temperate | Cfa | *Bacteroides HF183* |
| Evaluation of host-specific Bacteroidales 16S rRNA gene markers as a complementary tool for detecting fecal pollution in a prairie watershed. | Fremaux B; Gritzfeld J; Boa T; Yost CK | 2009 | Canada | North America | HIC | Cold | Dfb | *Bacteroides HF183* |
| Evaluation of Human- and Animal-Specific Viral Markers and Application of CrAssphage, Pepper Mild Mottle Virus, and Tobacco Mosaic Virus as Potential Fecal Pollution Markers to River Water in Japan. | Malla B; Makise K; Nakaya K; Mochizuki T; Yamada T; Haramoto E | 2019 | Japan | Asia | HIC | Cold | Dfb | CrAssphage |
| Evaluation of multiple sewage-associated Bacteroides PCR markers for sewage pollution tracking | Ahmed, W.; Goonetilleke, A.; Powell, D.; Gardner, T. | 2009 | Australia | Oceania | HIC | Arid | BSh | *Bacteroides HF183* |
| Evaluation of rapid methods and novel indicators for assessing microbiological beach water quality | Griffith, J.F.; Cao, Y.; McGee, C.D.; Weisberg, S.B. | 2009 | United States | North America | HIC | Temperate | Csa | *Methanobrevibacter smithii, Bacteroides thetaiotamicron, Human Polyomavirus, Enterococcus, Human Adenovirus* |
| Evaluation of the host specificity of Bacteroides thetaiotamicron alpha-1-6, mannanase gene as a sewage marker | Aslan, A.; Rose, J.B. | 2013 | United States | North America | HIC | Cold | Dfb | *Bacteroides thetaiotamicron, Bacteroides HF183* |
| Evaluation of the nifH gene marker of methanobrevibacter smithii for the detection of sewage pollution in environmental waters in southeast Queensland, Australia | Ahmed, W.; Sidhu, J.P.S.; Toze, S. | 2012 | Australia | Oceania | HIC | Arid | BSh | *Methanobrevibacter smithii* |
| Evaluation of the novel crAssphage marker for sewage pollution tracking in storm drain outfalls in Tampa, Florida. | Ahmed W; Lobos A; Senkbeil J; Peraud J; Gallard J; Harwood VJ | 2018 | Australia | Oceania | HIC | Temperate | Cfa | CrAssphage |
| Evaluation of two library-independent microbial source tracking methods to identify sources of fecal contamination in French estuaries. | Gourmelon M; Caprais MP; Ségura R; Le Mennec C; Lozach S; Piriou JY; Rincé A | 2007 | France | Europe | HIC | Temperate | Cfb | *Bacteroides HF183* |
| Evidence of viral dissemination and seasonality in a Mediterranean river catchment: Implications for water pollution management | Rusiñol, M.; Fernandez-Cassi, X.; Timoneda, N.; Carratalà, A.; Abril, J.F.; Silvera, C.; Figueras, M.J.; Gelati, E.; Rodó, X.; Kay, D.; Wyn-Jones, P.; Bofill-Mas, S.; Girones, R. | 2015 | Spain | Europe | HIC | Temperate | Cfb | *Human Adenovirus* |
| Fecal pollution source tracking in waters intended for human supply based on archaeal and bacterial genetic markers | Bianco, K.; Barreto, C.; Oliveira, S.S.; Pinto, L.H.; Albano, R.M.; Miranda, C.C.; Clementino, M.M. | 2015 | Brazil | South America | LMIC | Tropical | Aw | Methanobrevibacter smithii |
| Frequent detection of polyomaviruses in stool samples from hospitalized children | Vanchiere, John A; Nicome, Roger K; Greer, Jewel M; Demmler, Gail J; Butel, Janet S | 2005 | United States | North America | HIC | Temperate | Cfa | *Human Polyomavirus* |
| Frogs host faecal bacteria typically associated with humans | Gibb, K.; Schobben, X.; Christian, K. | 2017 | Australia | Oceania | HIC | Arid | BSh | B*acteroides thetaiotamicron, Enterococcus* |
| Highly Specific Sewage-Derived Bacteroides Quantitative PCR Assays Target Sewage-Polluted Waters. | Feng S; McLellan SL | 2019 | United States | North America | HIC | Cold | Dfb | *Bacteroides HF183* |
| Human and bovine adenoviruses for the detection of source-specific fecal pollution in coastal waters in Australia | Ahmed, W.; Goonetilleke, A.; Gardner, T. | 2010 | Australia | Oceania | HIC | Arid | BSh | *Human Adenovirus* |
| Human-associated Bacteroides spp. and human polyomaviruses as microbial source tracking markers in Hawaii | Kirs, M.; Caffaro-Filho, R.A.; Wong, M.; Harwood, V.J.; Moravcik, P.; Fujioka, R.S. | 2016 | United States | North America | HIC | Temperate | Cfb | *Bacteroides HF183, Human Polyomavirus* |
| Identification of human and animal adenoviruses and polyomaviruses for determination of sources of fecal contamination in the environment. | Hundesa A; Maluquer de Motes C; Bofill-Mas S; Albinana-Gimenez N; Girones R | 2006 | Spain | Europe | HIC | Temperate | Cfb | *Human Adenovirus* |
| Identifying human and livestock sources of fecal contamination in Kenya with host-specific Bacteroidales assays. | Jenkins MW; Tiwari S; Lorente M; Gichaba CM; Wuertz S | 2009 | Kenya | Africa | LMIC | Temperate | Cfb | *Bacteroides HF183* |
| Implementation and integration of microbial source tracking in a river watershed monitoring plan | Ballesté, E.; Demeter, K.; Masterson, B.; Timoneda, N.; Sala-Comorera, L.; Meijer, W.G. | 2020 | Ireland | Europe | HIC | Temperate | Cfb | *Bacteroides HF183* |
| Improvement of crAssphage detection/quantification method and its extensive application for food safety | Lee, S.-Y.; Yang, J.; Lee, J.-H. | 2023 | South Korea | Asia | HIC | Cold | Dwa | CrAssphage |
| Incidence of the enterococcal surface protein (esp) gene in human and animal fecal sources | Whitman, Richard L; Przybyla-Kelly, Katarzyna; Shively, Dawn A; Byappanahalli,  Muruleedhara N | 2007 | United States | North America | HIC | Cold | Dfa | *Enterococcus* |
| Integrated Multivariate Analysis with Nondetects for the Development of Human Sewage Source-Tracking Tools Using Bacteriophages of Enterococcus faecalis. | Wangkahad B; Mongkolsuk S; Sirikanchana K | 2017 | Thailand | Asia | LMIC | Tropical | Aw | *Enterococcus* |
| Microbial indicators and molecular markers used to differentiate the source of faecal pollution in the Bogotá River (Colombia) | Sánchez-Alfonso, A.C.; Venegas, C.; Díez, H.; Méndez, J.; Blanch, A.R.; Jofre, J.; Campos, C. | 2020 | Colombia | South America | LMIC | Tropical | Aw | *Bifidobacterium* |
| Microbial source markers assessment in the Bogotá River basin (Colombia). | Venegas C; Diez H; Blanch AR; Jofre J; Campos C | 2015 | Colombia | South America | LMIC | Tropical | Aw | *Bifidobacterium* |
| Microbial source tracking in shellfish harvesting waters in the Gulf of Nicoya, Costa Rica. | Symonds EM; Young S; Verbyla ME; McQuaig-Ulrich SM; Ross E; Jiménez JA; Harwood VJ; Breitbart M | 2017 | Costa Rica | North America | LMIC | Tropical | Aw | *Human Polyomavirus, Bacteroides HF183* |
| Microbial source tracking of untreated human wastewater and animal scats in urbanized estuarine waters | Ahmed, W.; Payyappat, S.; Cassidy, M.; Harrison, N.; Besley, C. | 2023 | Australia | Oceania | HIC | Arid | BSh | *Methanobrevibacter smithii, Human Polyomavirus,* CrAssphage*, Human Adenovirus, Bacteroides HF183* |
| Molecular Identification of Human Adenovirus Isolated from Different Wastewater  Treatment Plants in Riyadh, Saudi Arabia: Surveillance and Meteorological Impacts | Maniah, K.; Nour, I.; Hanif, A.; Yassin, M.T.; Alkathiri, A.; Al-Ashkar, I.; Eifan, S. | 2023 | Saudi Arabia | Asia | HIC | Arid | BWh | *Human Adenovirus* |
| Molecular indicators used in the development of predictive models for microbial source tracking. | Ballesté E; Bonjoch X; Belanche LA; Blanch AR | 2010 | Spain | Europe | HIC | Temperate | Cfb | *Bifidobacterium, Enterococcus, Bacteroides HF183* |
| Multiple approaches to microbial source tracking in tropical northern Australia | Neave, M.; Luter, H.; Padovan, A.; Townsend, S.; Schobben, X.; Gibb, K. | 2014 | Australia | Oceania | HIC | Arid | BSh | *Enterococcus* |
| Multiplex PCR with 16S rRNA gene-targeted primers of Bifidobacterium spp. to identify sources of fecal pollution | Bonjoch, X.; Ballesté, E.; Blanch, A.R. | 2004 | Spain | Europe | HIC | Temperate | Cfb | *Bifidobacterium catenulatum, Bifidobacterium* |
| New methods for the concentration of viruses from urban sewage using quantitative PCR | Calgua, B.; Rodriguez-Manzano, J.; Hundesa, A.; Suñen, E.; Calvo, M.; Bofill-Mas, S.; Girones, R. | 2013 | Spain | Europe | HIC | Temperate | Cfb | *Human Adenovirus* |
| New molecular quantitative PCR assay for detection of host-specific Bifidobacteriaceae suitable for microbial source tracking. | Gómez-Doñate M; Ballesté E; Muniesa M; Blanch AR | 2012 | Spain | Europe | HIC | Temperate | Cfb | *Bifidobacterium* |
| Occurrence of bacteriophages infecting Bacteroides host strains (ARABA 84 and GB-124) in fecal samples of human and animal origin | Diston, D.; Wicki, M. | 2015 | Switzerland | Europe | HIC | Temperate | Cfb | *Bacteroides thetaiotamicron* |
| PCR data and comparative performance of Bacteroidales microbial source tracking genetic markers | Somnark, P.; Chyerochana, N.; Kongprajug, A.; Mongkolsuk, S.; Sirikanchana, K. | 2018 | China | Asia | LMIC | Tropical | Aw | *Bacteroides HF183* |
| Performance Evaluation of Human-Specific Viral Markers and Application of Pepper Mild Mottle Virus and CrAssphage to Environmental Water Samples as Fecal Pollution Markers in the Kathmandu Valley, Nepal | Malla, B.; Ghaju Shrestha, R.; Tandukar, S.; Sherchand, J.B.; Haramoto, E. | 2019 | Nepal | Asia | LMIC | Cold | Dwc | *Human Polyomavirus, Human Adenovirus,* CrAssphage |
| Performance of forty-one microbial source tracking methods: a twenty-seven lab evaluation study | Boehm, Alexandria B; Van De Werfhorst, Laurie C; Griffith, John F; Holden, Patricia A; Jay, Jenny A; Shanks, Orin C; Wang, Dan; Weisberg, Stephen B | 2013 | United States | North America | HIC | Temperate | Csa | *BsteriF1, HumM2, Methanobrevibacter smithii, Bacteroides thetaiotamicron, Bacteroides HF183* |
| Performance of human fecal anaerobe-associated PCR-based assays in a multi-laboratory method evaluation study | Layton, B.A.; Cao, Y.; Ebentier, D.L.; Hanley, K.; Ballesté, E.; Brandão, J.; Byappanahalli, M.; Converse, R.; Farnleitner, A.H.; Gentry-Shields, J.; Gidley, M.L.; Gourmelon, M.; Lee, C.S.; Lee, J.; Lozach, S.; Madi, T.; Meijer, W.G.; Noble, R.; Peed, L.; Reischer, G.H.; Rodrigues, R.; Rose, J.B.; Schriewer, A.; Sinigalliano, C.; Srinivasan, S.; Stewart, J.; Van De Werfhorst, L.C.; Wang, D.; Whitman, R.; Wuertz, S.; Jay, J.; Holden, P.A.; Boehm, A.B.; Shanks, O.; Griffith, J.F. | 2013 | United States | North America | HIC | Temperate | Csa | *HumM2,* Bacteroides stercoris F1*, Methanobrevibacter smithii, Bacteroides thetaiotamicron, Bacteroides HF183* |
| Performance of two quantitative PCR methods for microbial source tracking of human sewage and implications for microbial risk assessment in recreational waters. | Staley C; Gordon KV; Schoen ME; Harwood VJ | 2012 | United States | North America | HIC | Temperate | Cfa | *Human Polyomavirus, Bacteroides HF183* |
| Performance of viral and bacterial genetic markers for sewage pollution tracking in tropical Thailand. | Sangkaew W; Kongprajug A; Chyerochana N; Ahmed W; Rattanakul S; Denpetkul T; Mongkolsuk S; Sirikanchana K | 2021 | Thailand | Asia | LMIC | Tropical | Aw | Human Polyomavirus, CrAssphage |
| Polyomavirus shedding in the stool of healthy adults | Vanchiere, John A; Abudayyeh, Suhaib; Copeland, Christina M; Lu, Lee B; Graham, David Y; Butel, Janet S | 2009 | United States | North America | HIC | Temperate | Cfa | *Human Polyomavirus* |
| Probabilistic analysis showing that a combination of Bacteroides and Methanobrevibacter source tracking markers is effective for identifying waters contaminated by human fecal pollution. | Johnston C; Byappanahalli MN; Gibson JM; Ufnar JA; Whitman RL; Stewart JR | 2013 | United States | North America | HIC | Cold | Dfa | *Methanobrevibacter smithii, Bacteroides HF183, Enterococcus* |
| Quantification and stability of human adenoviruses and polyomavirus JCPyV in wastewater matrices | Bofill-Mas, Silvia; Albinana-Gimenez, Nestor; Clemente-Casares, Pilar; Hundesa, Ayalkibet; Rodriguez-Manzano, Jesus; Allard, Annika; Calvo, Miquel; Girones, Rosina | 2006 | Spain | Europe | HIC | Temperate | Cfb | *Human Polyomavirus, Human Adenovirus* |
| Quantitative detection of human- and canine-associated Bacteroides genetic markers from an urban coastal lagoon. | Yasar SA; Mills TJT; Uluturk ZI; Ruszczyk JMS; LeBard RJ; Neilan BA | 2021 | Australia | Oceania | HIC | Arid | BSh | *Bacteroides HF183* |
| Quantitative identification of fecal water pollution sources by TaqMan real-time PCR assays using Bacteroidales 16S rRNA genetic markers. | Lee DY; Weir SC; Lee H; Trevors JT | 2010 | Canada | North America | HIC | Cold | Dfc | *BacHuman* |
| Quantitative risk assessment of norovirus and adenovirus for the use of reclaimed water to irrigate lettuce in Catalonia | Gonzales-Gustavson, E.; Rusiñol, M.; Medema, G.; Calvo, M.; Girones, R. | 2019 | Spain | Europe | HIC | Temperate | Cfb | *Human Adenovirus* |
| Rapid assessment of viral water quality using a novel recombinase polymerase amplification test for human adenovirus | Rames, E.K.; Macdonald, J. | 2019 | Australia | Oceania | HIC | Arid | BSh | *Human Adenovirus* |
| Sensitive detection of human adenovirus from small volume of primary wastewater samples by quantitative PCR | Sidhu, J.P.S.; Ahmed, W.; Toze, S. | 2013 | Australia | Oceania | HIC | Arid | BSh | *Human Adenovirus* |
| Simultaneous Detection of Selected Enteric Viruses in Water Samples by Multiplex Quantitative PCR | Lee, D.-Y.; Leung, K.T.; Lee, H.; Habash, M.B. | 2016 | Canada | North America | HIC | Cold | Dfc | *Human Adenovirus* |
| Sorbitol-fermenting bifidobacteria are indicators of very recent human faecal pollution in streams and groundwater habitats in urban tropical lowlands | Mushi, D.; Byamukama, D.; Kivaisi, A.K.; Mach, R.L.; Farnleitner, A.H. | 2010 | Tanzania | Africa | LMIC | Tropical | Aw | *Bifidobacterium* |
| Source tracking faecal contamination in an urbanised and a rural waterway in the Nelson-Tasman region, New Zealand | Kirs, M.; Harwood, V.J.; Fidler, A.E.; Gillespie, P.A.; Fyfe, W.R.; Blackwood, A.D.; Cornelisen, C.D | 2011 | New Zealand | Oceania | HIC | Temperate | Cfb | *Methanobrevibacter smithii, Human Polyomavirus, Enterococcus* |
| Specificity and sensitivity evaluation of novel and existing Bacteroidales and Bifidobacteria-specific PCR assays on feces and sewage samples and their application for microbial source tracking in Ireland | Dorai-Raj, S.; Grady, J.O.; Colleran, E. | 2009 | Ireland | Europe | HIC | Temperate | Cfb | *Bacteroides HF183, Bifidobacterium catenulatum, Bifidobacterium* |
| Specificity of a Bacteroides thetaiotaomicron marker for human feces | Carson, C.A.; Christiansen, J.M.; Yampara-Iquise, H.; Benson, V.W.; Baffaut, C.; Davis, J.V.; Broz, R.R.; Kurtz, W.B.; Rogers, W.M.; Fales, W.H. | 2005 | United States | North America | HIC | Temperate | Cfa | *Bacteroides thetaiotamicron, Bacteroides HF183* |
| The detection of Bifidobacterium adolescentis by colony hybridization as an indicator of human faecal pollution | Lynch, PA; Gilpin, BJ; Sinton, LW; Savill, MG | 2002 | New Zealand | Oceania | HIC | Temperate | Cfb | *Bifidobacterium* |
| Tracking the primary sources of fecal pollution in a tropical watershed in a one-year study. | Toledo-Hernandez C; Ryu H; Gonzalez-Nieves J; Huertas E; Toranzos GA; Santo Domingo JW | 2013 | Puerto Rico | North America | HIC | Tropical | Am | *Bacteroides HF183* |
| Use of a Bacteroides thetaiotaomicron‐specific α‐1‐6, mannanase quantitative PCR to detect human faecal pollution in water | Yampara‐Iquise, H; Zheng, G; Jones, JE; Carson, C Andrew | 2008 | United States | North America | HIC | Temperate | Cfa | *Bacteroides thetaiotamicron* |
| Use of Bifidobacterium dentium as an indicator of the origin of fecal water pollution | Nebra, Yolanda; Bonjoch, Xavier; Blanch, Anicet R | 2003 | Spain | Europe | HIC | Temperate | Cfb | *Bifidobacterium* |
| Use of viral pathogens and indicators to differentiate between human and non-human fecal contamination in a microbial source tracking comparison study. | Noble RT; Allen SM; Blackwood AD; Chu W; Jiang SC; Lovelace GL; Sobsey MD; Stewart JR; Wait DA | 2003 | United States | North America | HIC | Temperate | Csa | *Human Adenovirus* |
| Validation and field testing of library-independent microbial source tracking methods in the Gulf of Mexico | Harwood, V.J.; Brownell, M.; Wang, S.; Lepo, J.; Ellender, R.D.; Ajidahun, A.; Hellein, K.N.; Kennedy, E.; Ye, X.; Flood, C. | 2009 | United States | North America | HIC | Temperate | Cfa | *Human Polyomavirus, Methanobrevibacter smithii* |
| Validation of Bacteroidales quantitative PCR assays targeting human and animal fecal contamination in the public and domestic domains in India. | Odagiri M; Schriewer A; Hanley K; Wuertz S; Misra PR; Panigrahi P; Jenkins MW | 2015 | India | Asia | LMIC | Tropical | Aw | *HumM2, Bacteroides HF183* |
| Validation of host-specific Bacteriodales 16S rRNA genes as markers to determine the origin of faecal pollution in Atlantic Rim countries of the European Union | Gawler, A.H.; Beecher, J.E.; Brandão, J.; Carroll, N.M.; Falcão, L.; Gourmelon, M.; Masterson, B.; Nunes, B.; Porter, J.; Rincé, A.; Rodrigues, R.; Thorp, M.; Martin Walters, J.; Meijer, W.G. | 2007 | France, Ireland, Portugal, United Kingdom | Europe | HIC | Temperate | Cfb, Csa | *Bacteroides HF183* |
| Validation of host-specific Bacteroidales quantitative PCR assays and their application to microbial source tracking of drinking water sources in the Kathmandu Valley, Nepal | Malla, B.; Ghaju Shrestha, R.; Tandukar, S.; Bhandari, D.; Inoue, D.; Sei, K.; Tanaka, Y.; Sherchand, J.B.; Haramoto, E. | 2018 | Nepal | Asia | LMIC | Cold | Dwc | *Bacteroides HF183* |
| Validation of microbial source tracking markers for the attribution of fecal contamination in indoor-household environments of the Peruvian Amazon. | Schiaffino F; Pisanic N; Colston JM; Rengifo D; Paredes Olortegui M; Shapiama V; Peñataro Yori P; Heaney CD; Davis MF; Kosek MN | 2020 | Peru | South America | LMIC | Tropical | Af | *Bacteroides HF183* |
| Variably improved microbial source tracking with digital droplet PCR. | Nshimyimana JP; Cruz MC; Wuertz S; Thompson JR | 2019 | Singapore | Asia | HIC | Tropical | Af | *Bacteroides thetaiotamicron* |
| Viral multiplex quantitative PCR assays for tracking sources of fecal contamination | Wolf, S.; Hewitt, J.; Greening, G.E. | 2010 | New Zealand | Oceania | HIC | Temperate | Cfb | *Human Adenovirus* |

# **Supplementary Material 4.0 – Systematic Review and Meta-Analysis Analysis Results**

**Figure S4. 1:** Bar chart showing the Sensitivity (pink), Specificity (Blue) and Accuracy (Purple) of the BacHuman human specific faecal marker across all geographic groupings with a dotted red line indicating the 80% performance threshold set out by Boehm et al.^1^ With the number of samples used to calculate this percentage shown in the bar themselves, and the error bars representing the 95% upper and lower confidence interval of each performance statistic. Created in Microsoft Office Excel 2019.


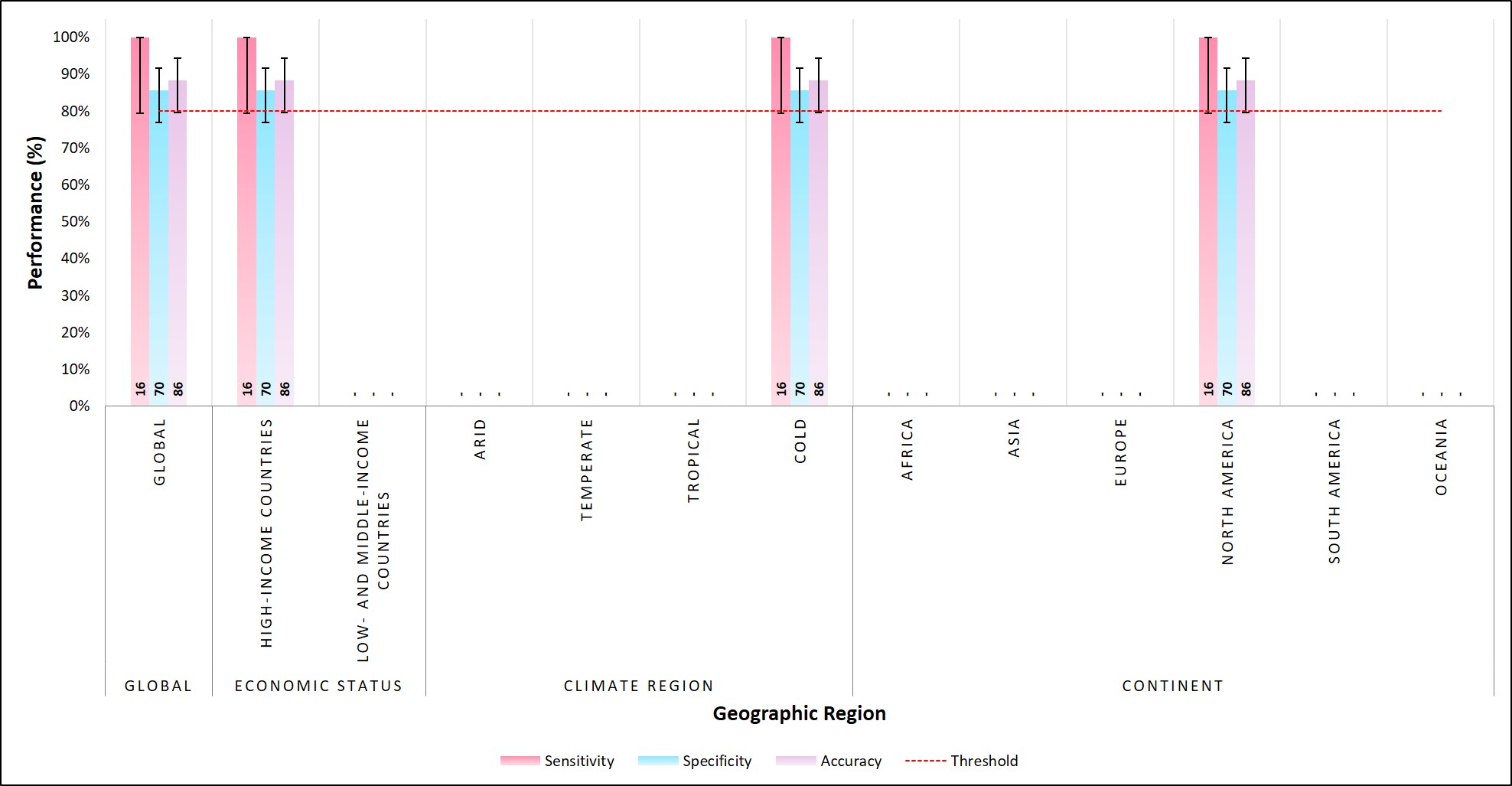


**Figure S4. 2**: Bar chart showing the Sensitivity (pink), Specificity (Blue) and Accuracy (Purple) of the Bacteroides HF183 human specific faecal marker across all geographic groupings with a dotted red line indicating the 80% performance threshold set out by Boehm et al.^1^ With the number of samples used to calculate this percentage shown in the bar themselves, and the error bars representing the 95% upper and lower confidence interval of each performance statistic. Created in Microsoft Office Excel 2019.


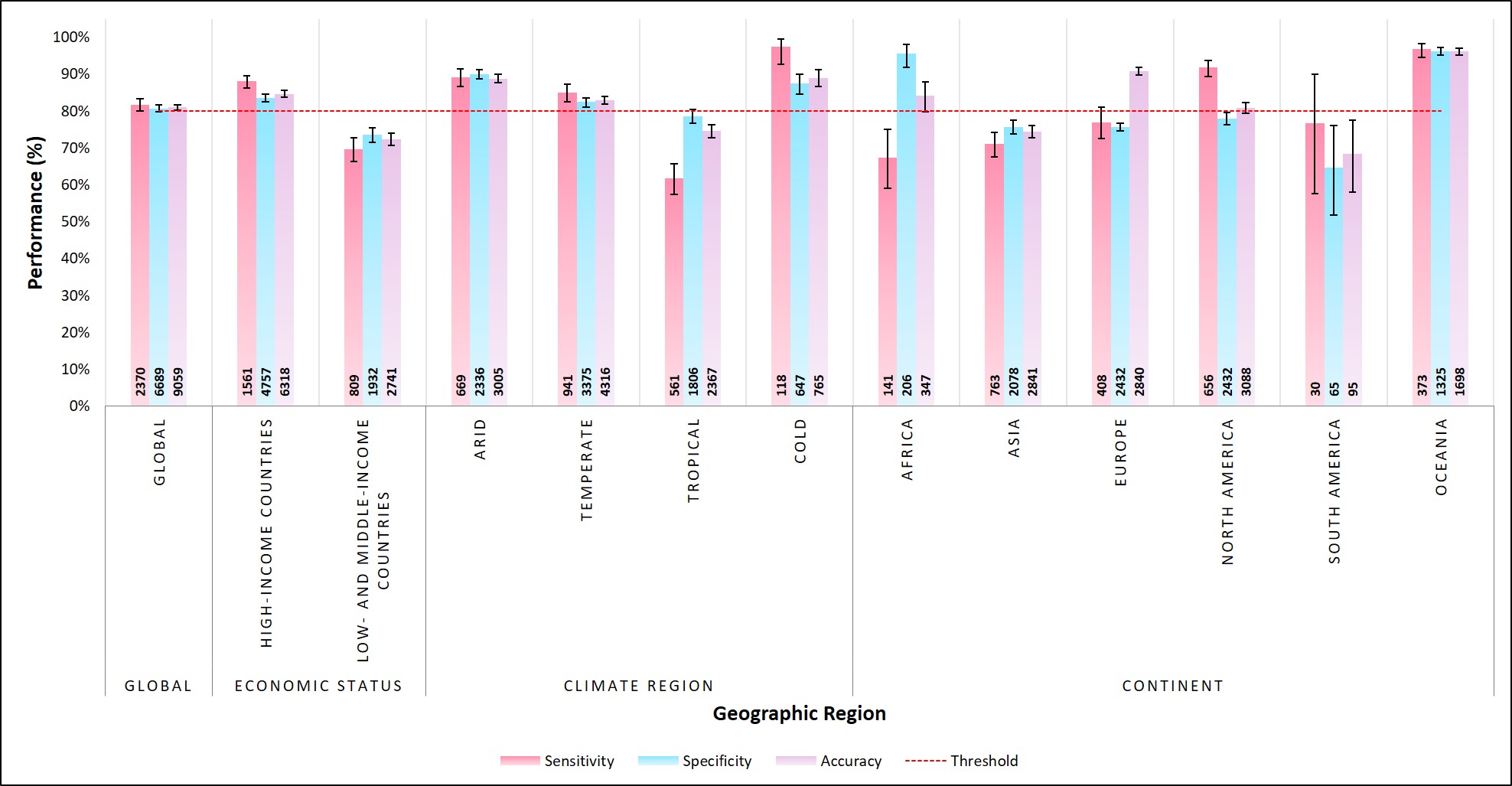


**Figure S4.3**: Bar chart showing the Sensitivity (pink), Specificity (Blue) and Accuracy (Purple) of the Bacteroides Thetaiotamicron human specific faecal marker across all geographic groupings with a dotted red line indicating the 80% performance threshold set out by Boehm et al.^1^ With the number of samples used to calculate this percentage shown in the bar themselves, and the error bars representing the 95% upper and lower confidence interval of each performance statistic. Created in Microsoft Office Excel 2019.


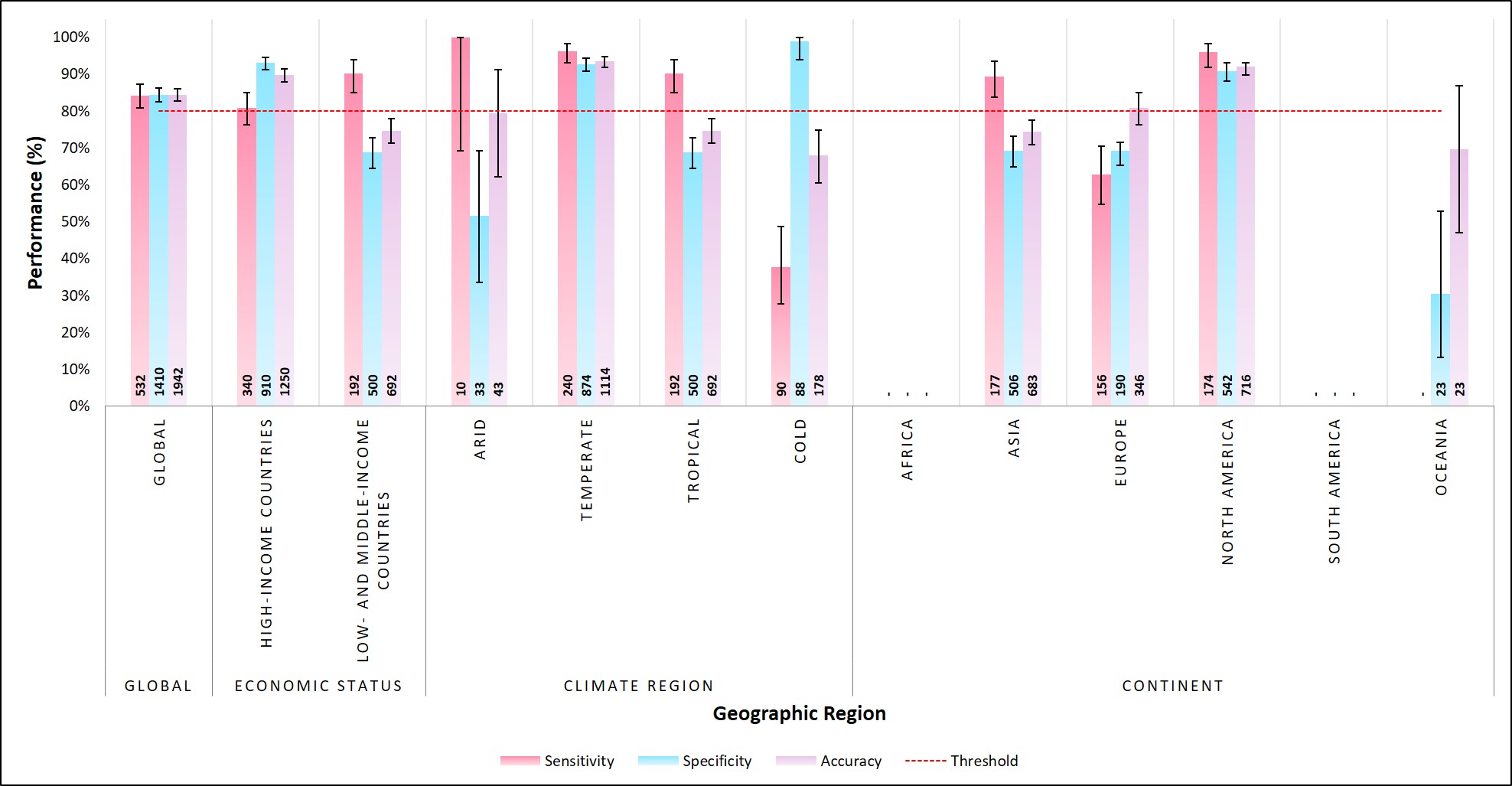


**Figure S4.4:** Bar chart showing the Sensitivity (pink), Specificity (Blue) and Accuracy (Purple) of the Bifidobacterium human specific faecal marker across all geographic groupings with a dotted red line indicating the 80% performance threshold set out by Boehm et al.^1^. With the number of samples used to calculate this percentage shown in the bar themselves, and the error bars representing the 95% upper and lower confidence interval of each performance statistic. Created in Microsoft Office Excel 2019.


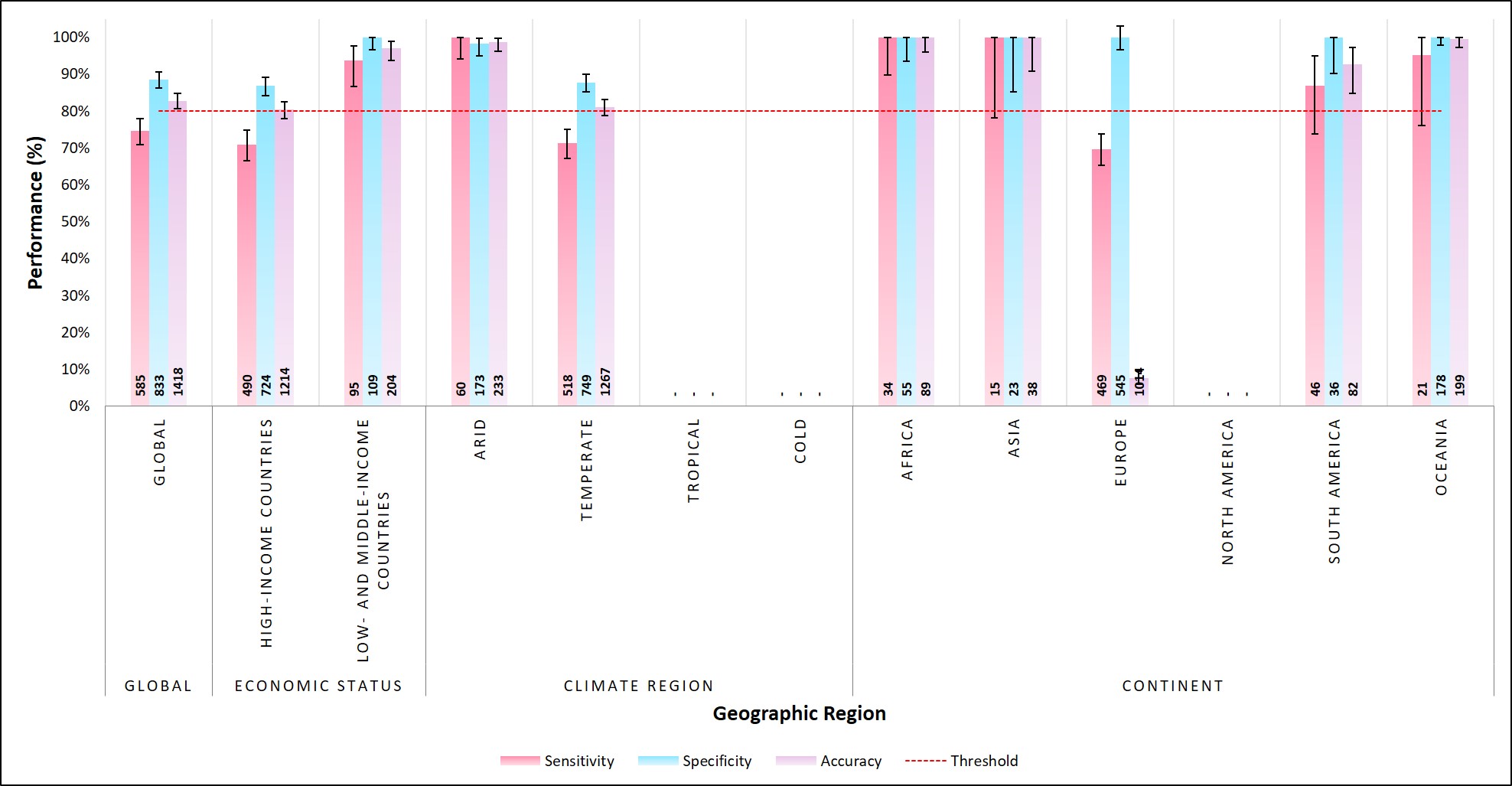


**Figure S4.5**: Bar chart showing the Sensitivity (pink), Specificity (Blue) and Accuracy (Purple) of the Bifidobacterium Catenulatum human specific faecal marker across all geographic groupings with a dotted red line indicating the 80% performance threshold set out by Boehm et al.^1^ With the number of samples used to calculate this percentage shown in the bar themselves, and the error bars representing the 95% upper and lower confidence interval of each performance statistic. Created in Microsoft Office Excel 2019.


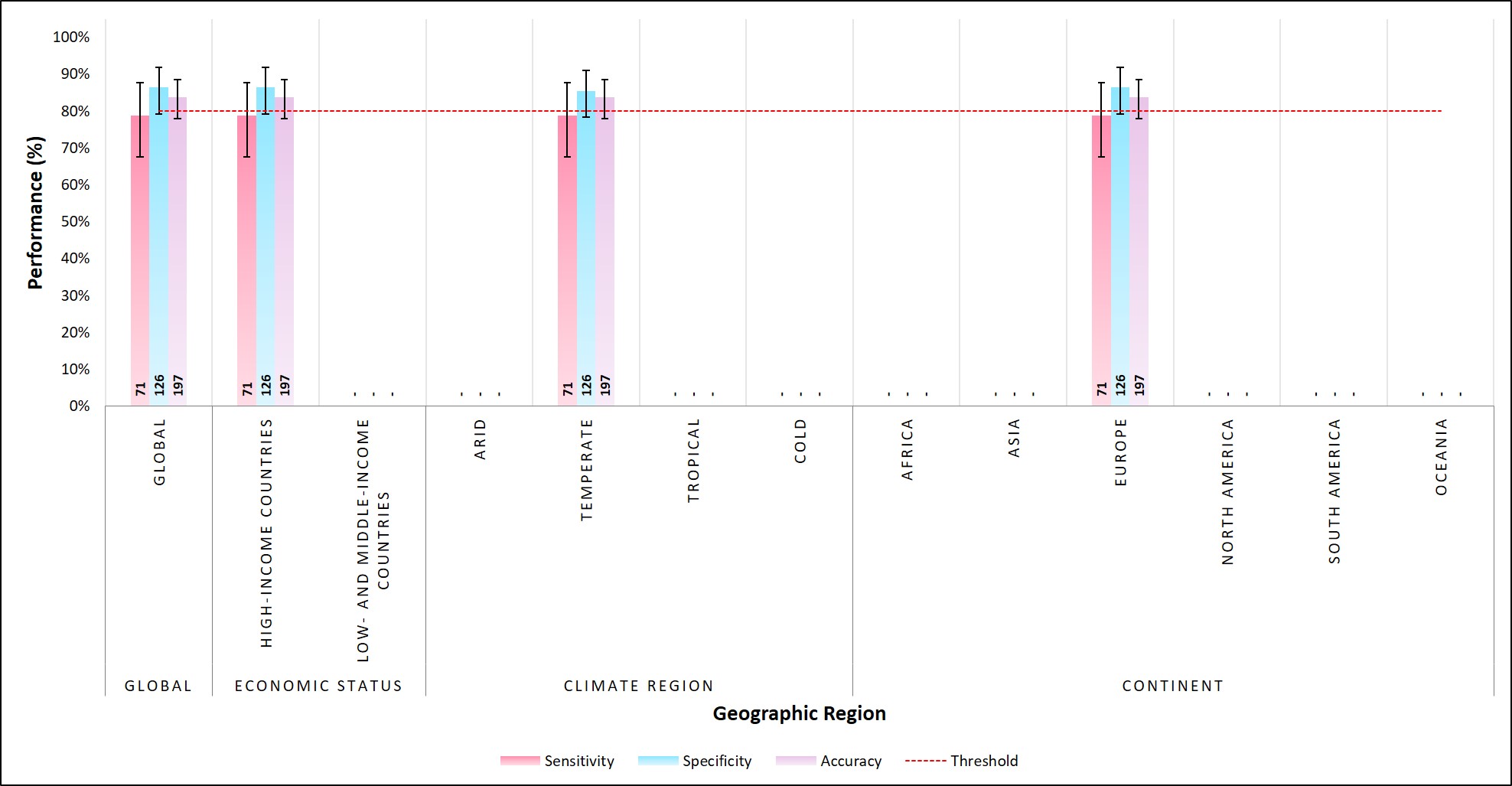


**Figure S4.6:** Bar chart showing the Sensitivity (pink), Specificity (Blue) and Accuracy (Purple) of the Bacteroides Stercoris F1 human specific faecal marker across all geographic groupings with a dotted red line indicating the 80% performance threshold set out by Boehm et al.^1^ With the number of samples used to calculate this percentage shown in the bar themselves, and the error bars representing the 95% upper and lower confidence interval of each performance statistic. Created in Microsoft Office Excel 2019.


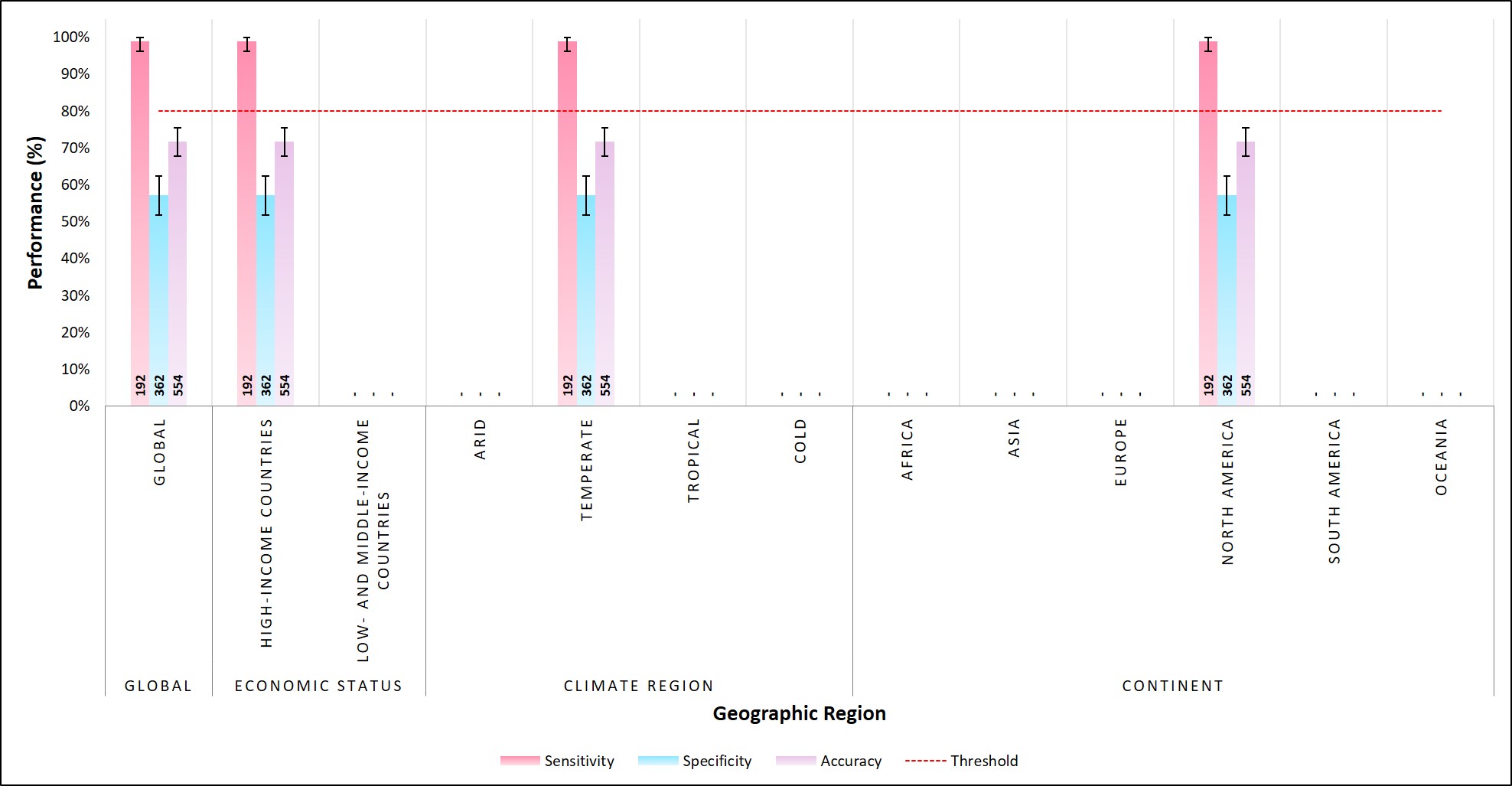


**Figure S4.7**: Bar chart showing the Sensitivity (pink), Specificity (Blue) and Accuracy (Purple) of the CrAssphage human specific faecal marker across all geographic groupings with a dotted red line indicating the 80% performance threshold set out by Boehm et al.^1^ With the number of samples used to calculate this percentage shown in the bar themselves, and the error bars representing the 95% upper and lower confidence interval of each performance statistic. Created in Microsoft Office Excel 2019.


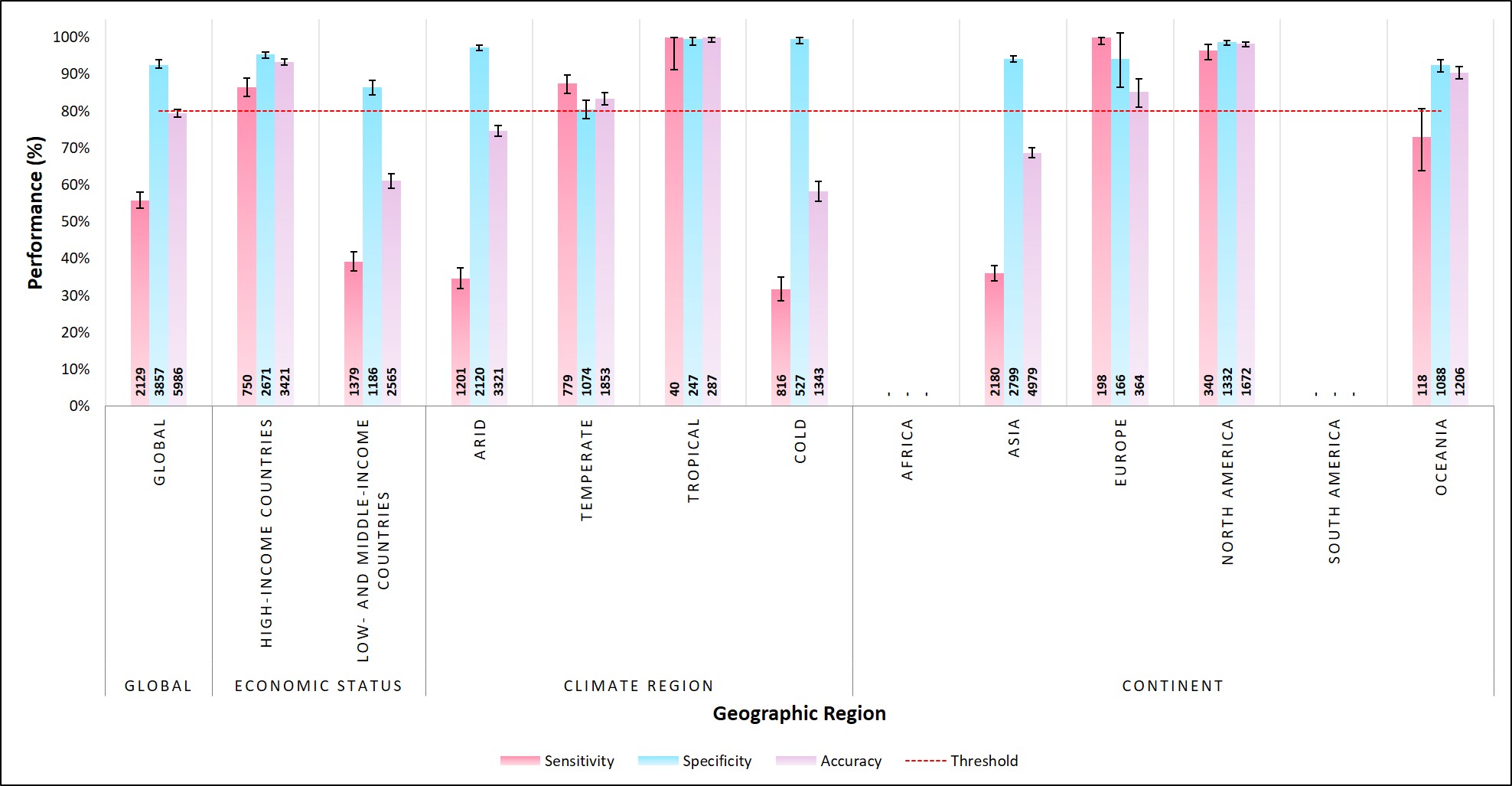


**Figure S4.8:** Bar chart showing the Sensitivity (pink), Specificity (Blue) and Accuracy (Purple) of the Enterococcus human specific faecal marker across all geographic groupings with a dotted red line indicating the 80% performance threshold set out by Boehm et al.^1^ With the number of samples used to calculate this percentage shown in the bar themselves, and the error bars representing the 95% upper and lower confidence interval of each performance statistic. Created in Microsoft Office Excel 2019.


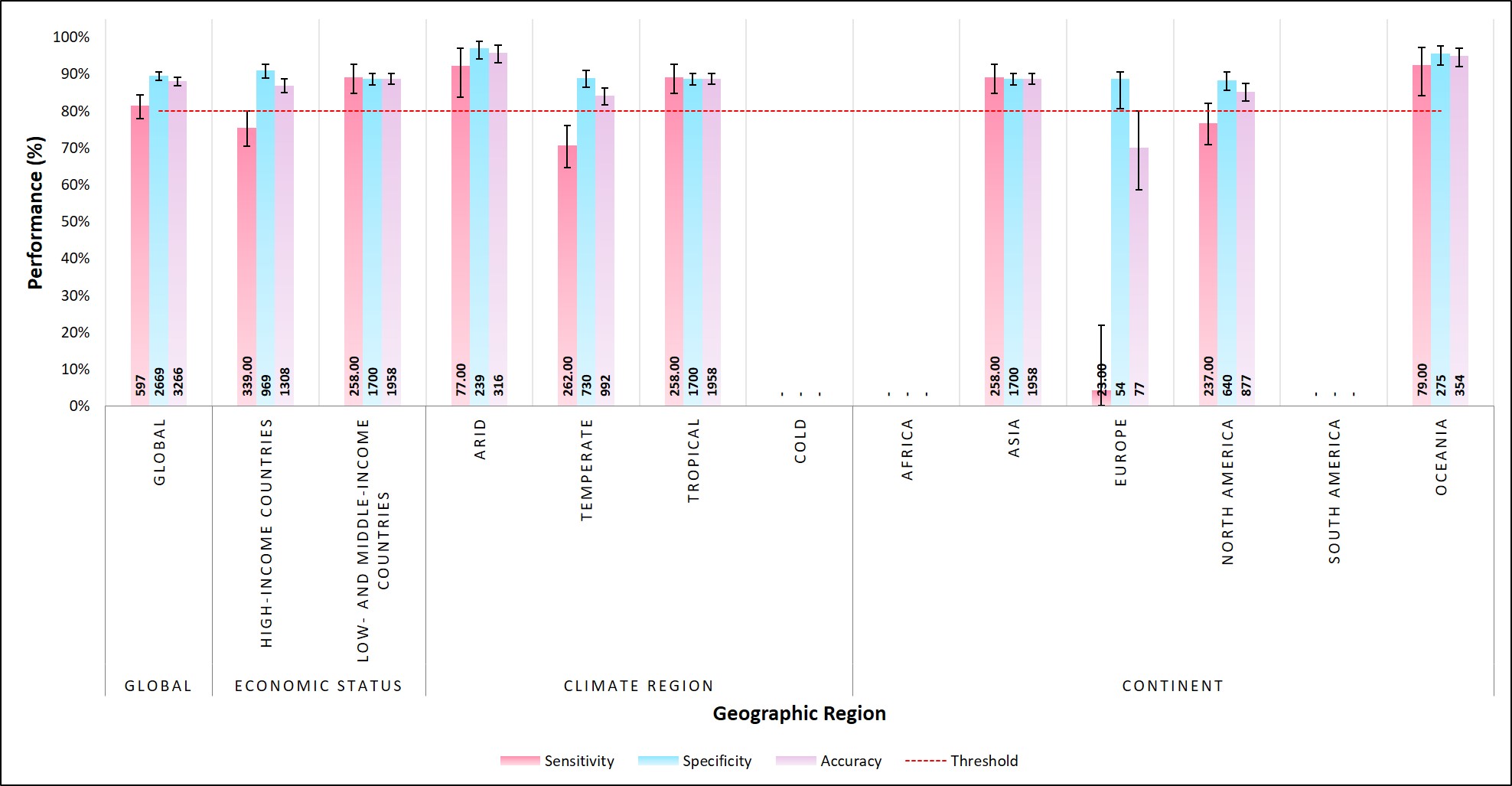


**Figure S4.9**: Bar chart showing the Sensitivity (pink), Specificity (Blue) and Accuracy (Purple) of the Faecalibacterium human specific faecal marker across all geographic groupings with a dotted red line indicating the 80% performance threshold set out by Boehm et al.^1^ With the number of samples used to calculate this percentage shown in the bar themselves, and the error bars representing the 95% upper and lower confidence interval of each performance statistic. Created in Microsoft Office Excel 2019.


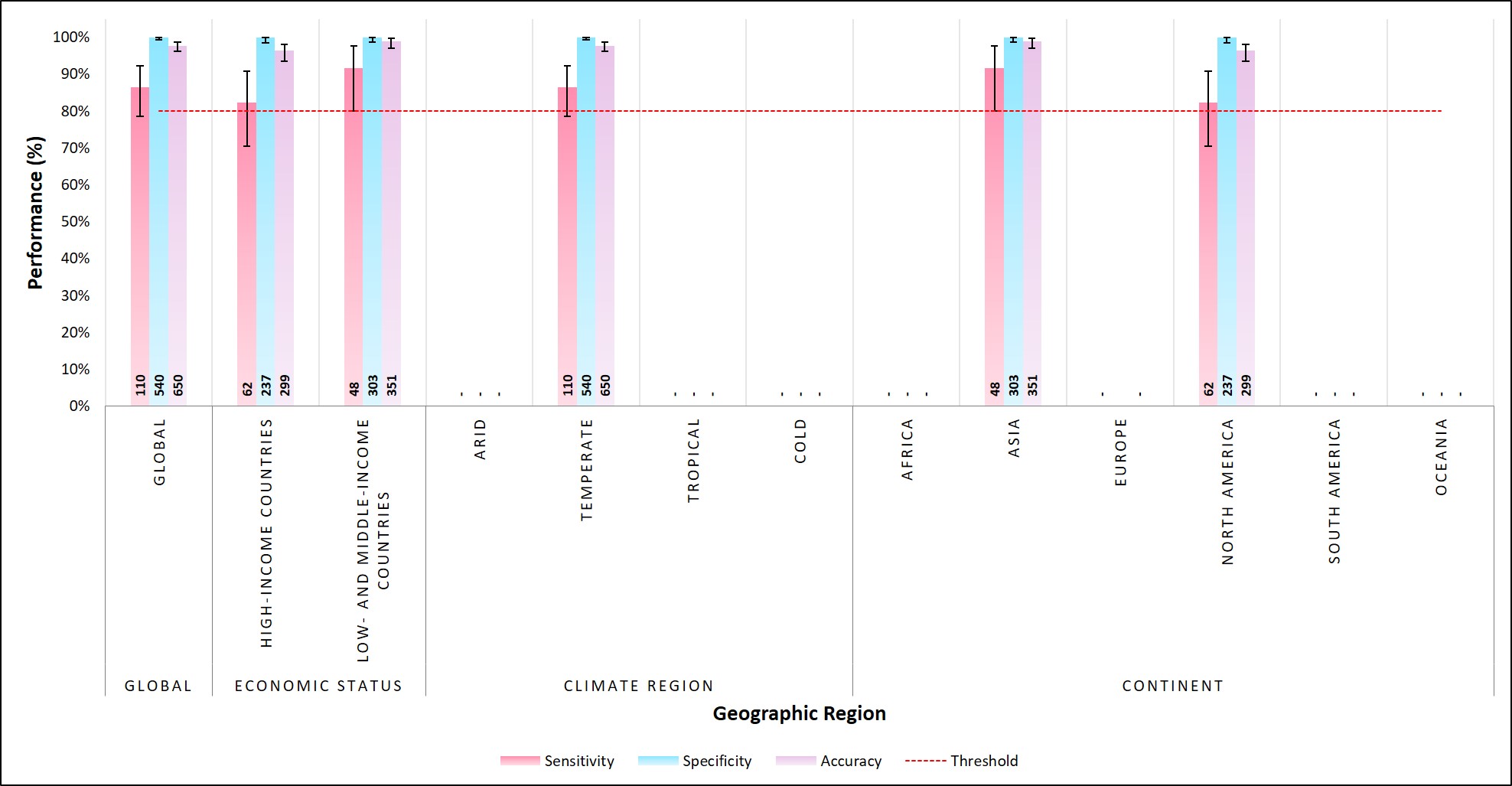


**Figure S4.10:** Bar chart showing the Sensitivity (pink), Specificity (Blue) and Accuracy (Purple) of the Human Adenovirus human specific faecal marker across all geographic groupings with a dotted red line indicating the 80% performance threshold set out by Boehm et al.^1^ With the number of samples used to calculate this percentage shown in the bar themselves, and the error bars representing the 95% upper and lower confidence interval of each performance statistic. Created in Microsoft Office Excel 2019.


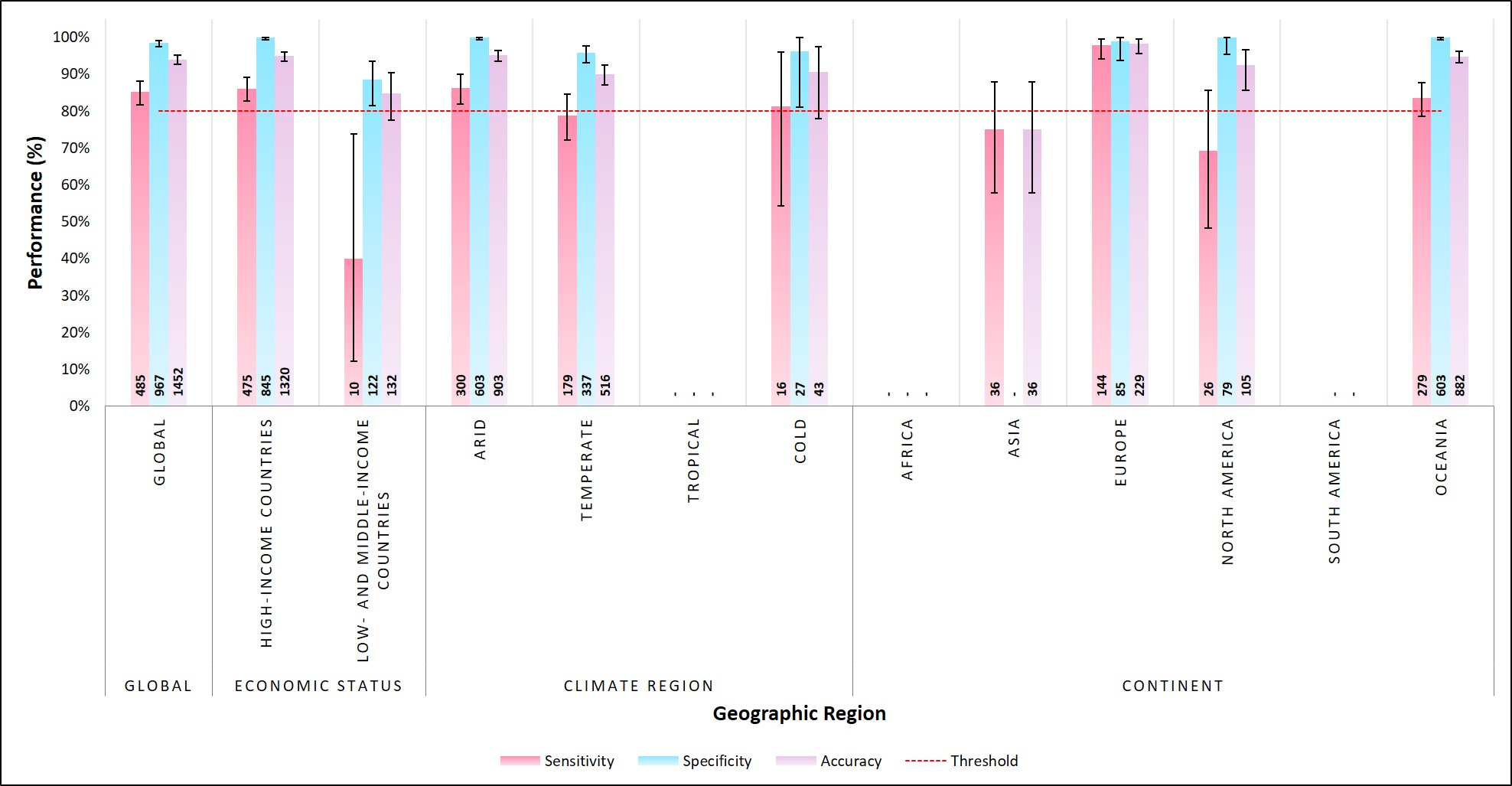


**Figure S4.11**: Bar chart showing the Sensitivity (pink), Specificity (Blue) and Accuracy (Purple) of the Human Polyomavirus human specific faecal marker across all geographic groupings with a dotted red line indicating the 80% performance threshold set out by Boehm et al.^1^ With the number of samples used to calculate this percentage shown in the bar themselves, and the error bars representing the 95% upper and lower confidence interval of each performance statistic. Created in Microsoft Office Excel 2019.


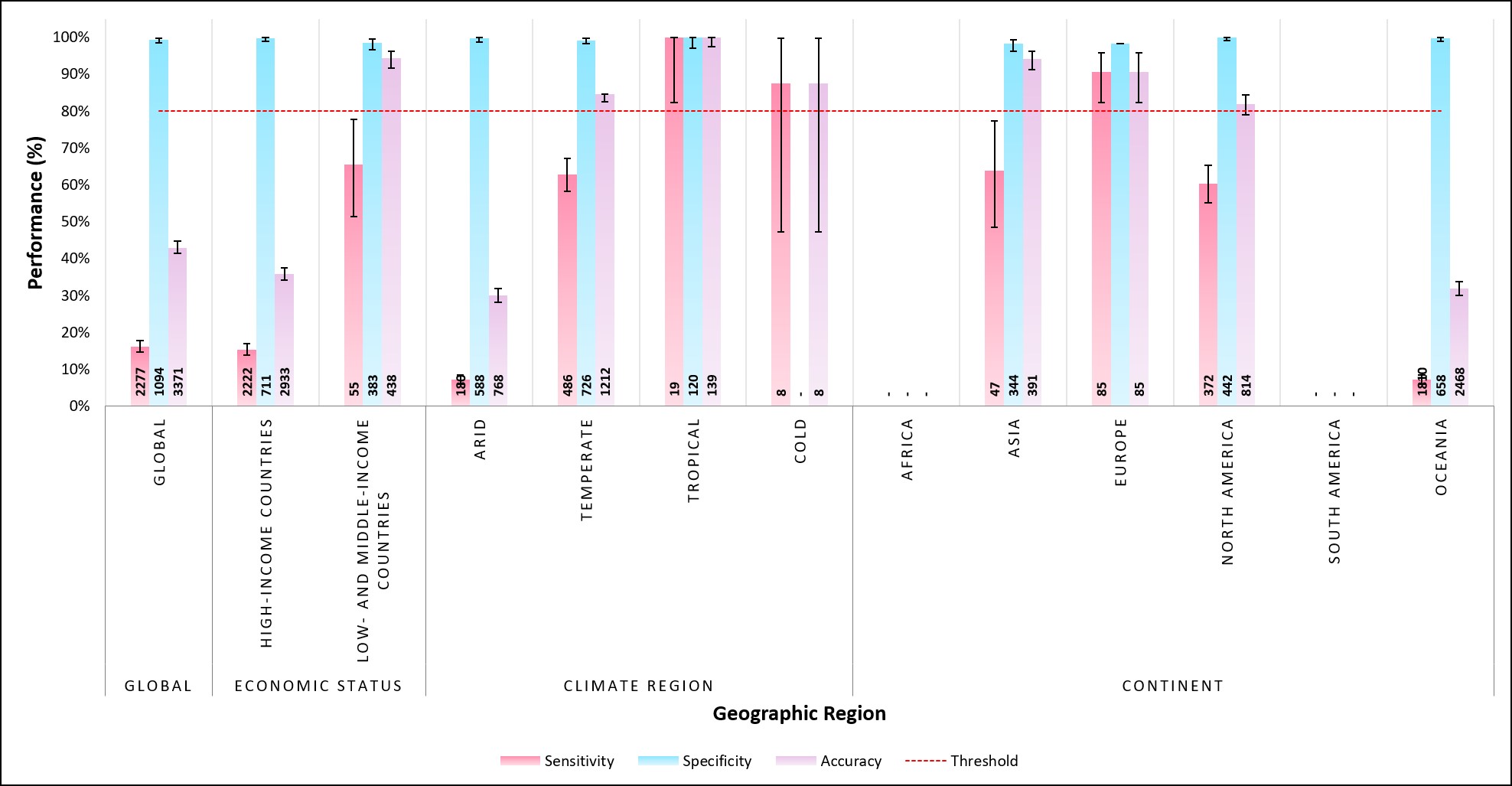


**Figure S4.12**: Bar chart showing the Sensitivity (pink), Specificity (Blue) and Accuracy (Purple) of the HumM2 human specific faecal marker across all geographic groupings with a dotted red line indicating the 80% performance threshold set out by Boehm et al.^1^ With the number of samples used to calculate this percentage shown in the bar themselves, and the error bars representing the 95% upper and lower confidence interval of each performance statistic. Created in Microsoft Office Excel 2019.


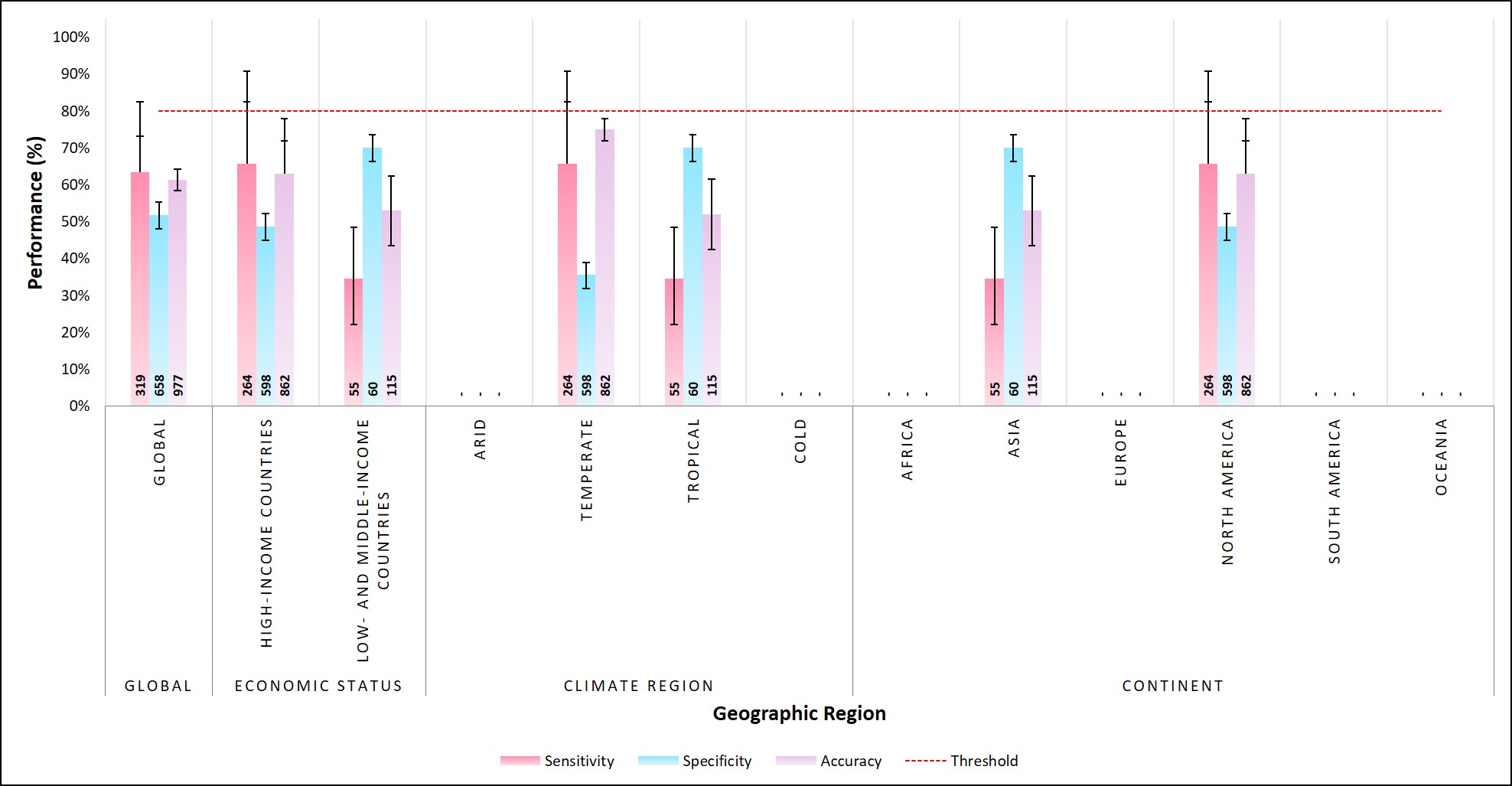


**Figure S4.13**: Bar chart showing the Sensitivity (pink), Specificity (Blue) and Accuracy (Purple) of the Methanobrevibacter Smithii human specific faecal marker across all geographic groupings with a dotted red line indicating the 80% performance threshold set out by Boehm et al.^1^ With the number of samples used to calculate this percentage shown in the bar themselves, and the error bars representing the 95% upper and lower confidence interval of each performance statistic. Created in Microsoft Office Excel 2019.


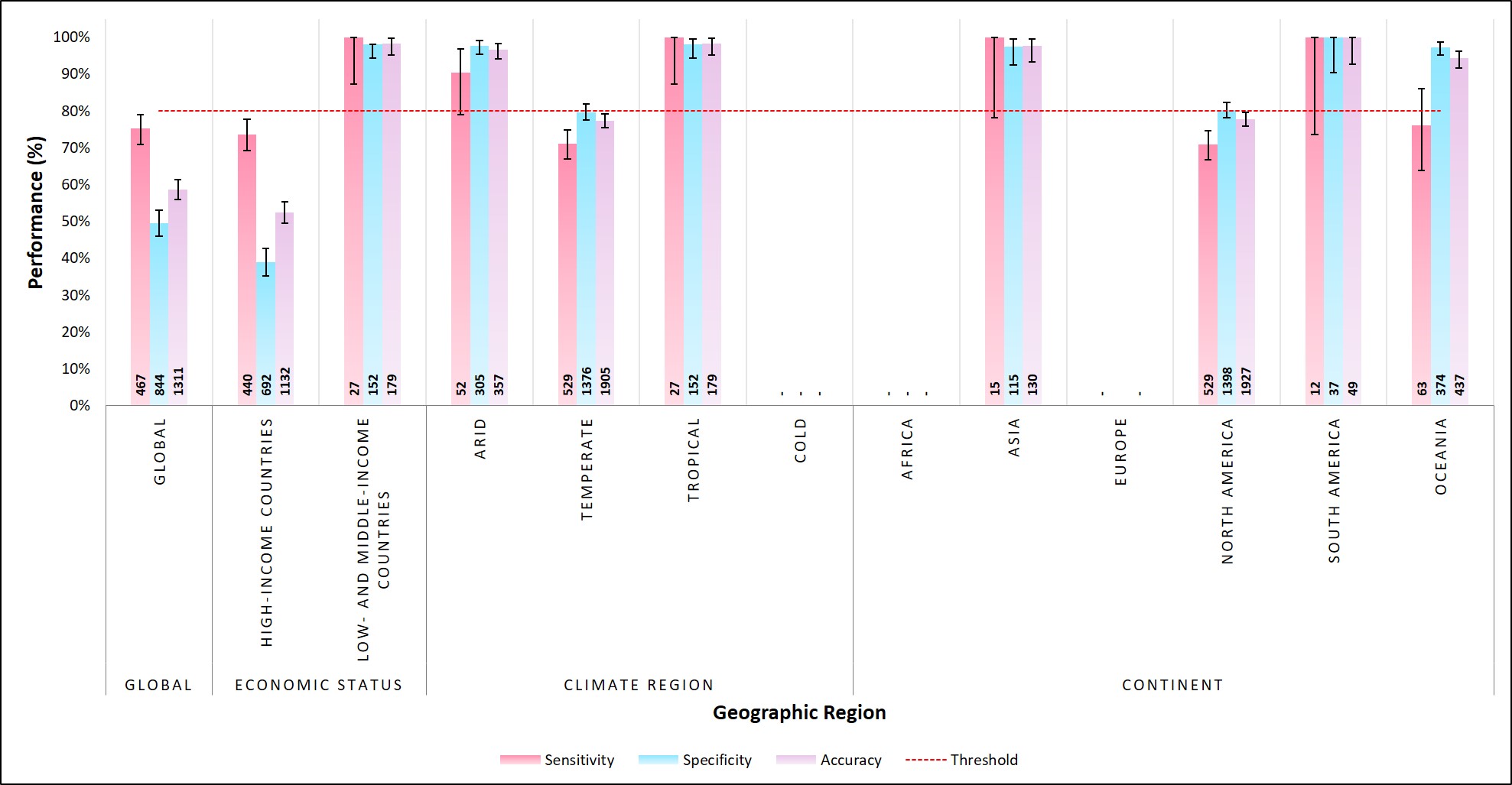


**Table S4.1:** Correlation matrix of overall climate zones vs sub climate zones, using agricolae; PB-Perfect from RStudio Version 4.3.0.

|  | **Arid** | **Temperate** | **Tropical** | **Cold** | **Af** | **Am** | **Aw** | **BSh** | **BWh** | **Cfa** | **Cfb** | **Csa** | **Cwa** | **Cwb** | **Dfa** | **Dfb** | **Dfc** | **Dwa** | **Dwc** |
| --- | --- | --- | --- | --- | --- | --- | --- | --- | --- | --- | --- | --- | --- | --- | --- | --- | --- | --- | --- |
| **Arid** | 1.00 | 0.33 | 0.60 | 0.41 | 0.27 | 0.28 | 0.67 | 1.00 | 0.23 | 0.13 | 0.65 | 0.29 | 0.40 | -0.19 | 0.37 | 0.32 | 0.04 | 0.25 | 0.57 |
| **Temperate** |  | 1.00 | 0.21 | -0.20 | 0.05 | 0.04 | 0.18 | 0.33 | 0.12 | 0.44 | 0.44 | 0.17 | 0.04 | 0.03 | 0.14 | 0.26 | -0.39 | 0.20 | 0.38 |
| **Tropical** |  |  | 1.00 | 0.37 | 0.41 | 0.17 | 0.89 | 0.60 | -0.28 | 0.41 | 0.49 | 0.35 | 0.32 | 0.23 | 0.68 | 0.37 | -0.14 | 0.28 | 0.16 |
| **Cold** |  |  |  | 1.00 | 0.46 | 0.25 | 0.23 | 0.41 | 0.09 | 0.33 | 0.36 | 0.03 | -0.15 | 0.18 | 0.57 | 0.35 | 0.47 | 0.14 | 0.38 |
| **Af** |  |  |  |  | 1.00 | 0.50 | 0.17 | 0.27 | -0.13 | 0.48 | 0.16 | 0.34 | 0.14 | -0.13 | 0.60 | 0.41 | 0.09 | -0.14 | 0.05 |
| **Am** |  |  |  |  |  | 1.00 | 0.16 | 0.28 | -0.07 | 0.13 | 0.22 | 0.23 | 0.49 | -0.07 | 0.31 | 0.54 | 0.51 | -0.07 | 0.41 |
| **Aw** |  |  |  |  |  |  | 1.00 | 0.66 | -0.24 | 0.22 | 0.49 | 0.24 | 0.34 | 0.25 | 0.49 | 0.19 | -0.08 | 0.31 | 0.22 |
| **BSh** |  |  |  |  |  |  |  | 1.00 | 0.25 | 0.12 | 0.65 | 0.29 | 0.39 | -0.19 | 0.36 | 0.31 | 0.04 | 0.25 | 0.57 |
| **BWh** |  |  |  |  |  |  |  |  | 1.00 | -0.21 | 0.15 | 0.02 | -0.09 | -0.05 | -0.19 | -0.12 | 0.42 | -0.06 | 0.30 |
| **Cfa** |  |  |  |  |  |  |  |  |  | 1.00 | 0.22 | -0.21 | -0.19 | 0.15 | 0.50 | 0.52 | -0.10 | 0.33 | 0.29 |
| **Cfb** |  |  |  |  |  |  |  |  |  |  | 1.00 | 0.000 | 0.27 | 0.17 | 0.44 | 0.41 | 0.09 | 0.26 | 0.47 |
| **Csa** |  |  |  |  |  |  |  |  |  |  |  | 1.00 | 0.42 | 0.13 | 0.21 | -0.04 | 0.01 | -0.34 | -0.06 |
| **Cwa** |  |  |  |  |  |  |  |  |  |  |  |  | 1.00 | -0.09 | 0.00 | 0.15 | 0.12 | -0.10 | 0.02 |
| **Cwb** |  |  |  |  |  |  |  |  |  |  |  |  |  | 1.00 | 0.33 | -0.12 | 0.37 | -0.06 | -0.08 |
| **Dfa** |  |  |  |  |  |  |  |  |  |  |  |  |  |  | 1.00 | 0.51 | 0.10 | 0.07 | 0.11 |
| **Dfb** |  |  |  |  |  |  |  |  |  |  |  |  |  |  |  | 1.00 | 0.14 | 0.48 | 0.51 |
| **Dfc** |  |  |  |  |  |  |  |  |  |  |  |  |  |  |  |  | 1.00 | -0.15 | 0.30 |
| **Dwa** |  |  |  |  |  |  |  |  |  |  |  |  |  |  |  |  |  | 1.00 | 0.37 |
| **Dwc** |  |  |  |  |  |  |  |  |  |  |  |  |  |  |  |  |  |  | 1.00 |
| Significance codes: <0.001 (green), <0.01 (yellow), <0.05 (red) | | | | | | | | | | | | | | | | | | | |
| Source: PCA - Data analysis | | | | | | | | | | | | | | | | | | | |

Table S4.2: Matrix of human specific MST markers with sensitivity and specificity performance metrics above the 80% threshold.^1^ With those with a lower confidence interval below the 80% threshold illustrated with * for sensitivity, ** for specificity and *** for both. No marker validated in Location is indicated with NMVL.

|  | **High Income Countries** | **Low- and Middle-Income Countries** | **Arid** | **Temperate** | **Tropical** | **Cold** | **Africa** | **Asia** | **Europe** | **North America** | **South America** | **Oceania** |
| --- | --- | --- | --- | --- | --- | --- | --- | --- | --- | --- | --- | --- |
| **High Income Country** | *BacHuman****  *Bacteroides HF183*  Human Adenovirus |  | *Bacteroides HF183*  *Bifidobacterium**  *CrAssphage**  *Enterococcus*  *Human Adenovirus*  *Methanobrevibacter smithii** | *CrAssphage***  *Human Adenovirus* | **NMVL** | *BacHuman****  *Bacteroides HF183** | **NMVL** | **NMVL** | Human Adenovirus | *BacHuman****  *Bacteroides thetaiotamicron*  CrAssphage | **NMVL** | *Bacteroides HF183*  *Bifidobacterium**  CrAssphage  *Enterococcus*  Human Adenovirus |
| **Low- and Middle-Income Countries** |  | *Bifidobacterium*  CrAssphage  *Enterococcus*  *Faecalibacterium Methanobrevibacter smithii** | **NMVL** | *Bifidobacterium*  *Faecalibacterium* | *Bifidobacterium*  *CrAssphage*  *Enterococcus*  *Human Polyomavirus* Methanobrevibacter smithii** | **NMVL** | *Bifidobacterium* | *Bifidobacterium**  CrAssphage  *Enterococcus*  *Faecalibacterium*  Human Polyomavirus* | **NMVL** | **NMVL** | *Bifidobacterium**  *Methanobrevibacter smithii** | ***NMVL*** |
| **Arid** |  |  | *Bacteroides HF183*  *Bifidobacterium*  CrAssphage*  *Enterococcus*  Human Adenovirus  *Methanobrevibacter smithii** |  |  |  | **NMVL** | **NMVL** | **NMVL** | **NMVL** | **NMVL** | *Bacteroides HF183*  *Bifidobacterium**  CrAssphage*  *Enterococcus*  Human Adenovirus *Methanobrevibacter smithii** |
| **Temperate** |  |  |  | CrAssphage  *Faecalibacterium*  Human Adenovirus* |  |  | *Bifidobacterium**** | *Bacteroides HF183**  *Bifidobacterium**  *Faecalibacterium* | Human Adenovirus | *Bacteroides thetaiotamicron*  CrAssphage | **NMVL** | *Bifidobacterium**  CrAssphage***  *Enterococcus**** |
| **Tropical** |  |  |  |  | *Bifidobacterium*  CrAssphage  *Enterococcus*  Human Polyomavirus*  *Methanobrevibacter smithii** |  | *Bifidobacterium* | CrAssphage  *Enterococcus*  Human Polyomavirus* | **NMVL** | **NMVL** | *Bifidobacterium*  *Methanobrevibacter smithii** | **NMVL** |
| **Cold** |  |  |  |  |  | *BacHuman****  *Bacteroides HF183* | **NMVL** | **NMVL** | **NMVL** | *BacHuman****  *Bacteroides HF183** | **NMVL** | **NMVL** |
| **Africa** |  |  |  |  |  |  | *Bifidobacterium* |  |  |  |  |  |
| **Asia** |  |  |  |  |  |  |  | *Bifidobacterium**  *Enterococcus*  *Faecalibacterium*  *Methanobrevibacter smithii** |  |  |  |  |
| **Europe** |  |  |  |  |  |  |  |  | CrAssphage  Human Adenovirus |  |  |  |
| **North America** |  |  |  |  |  |  |  |  |  | *BacHuman****  *Bacteroides thetaiotamicron*  CrAssphage |  |  |
| **South America** |  |  |  |  |  |  |  |  |  |  | Bifidobacterium*  Methanobrevibacter smithii* |  |
| **Oceania** |  |  |  |  |  |  |  |  |  |  |  | *Bacteroides HF183*  *Bifidobacterium**  CrAssphage  *Enterococcus*  Human Adenovirus |

# References

1. Boehm AB, Van De Werfhorst LC, Griffith JF, et al. Performance of forty-one microbial source tracking methods: a twenty-seven lab evaluation study. *Water Res* 2013; **47**:6812–28.
